# Phaeoviral Infections Are Present in *Macrocystis*, *Ecklonia* and *Undaria* (Laminariales) and Are Influenced by Wave Exposure in Ectocarpales

**DOI:** 10.3390/v10080410

**Published:** 2018-08-05

**Authors:** Dean A. McKeown, Joanna L. Schroeder, Kim Stevens, Akira F. Peters, Claudio A. Sáez, Jihae Park, Mark D. Rothman, John J. Bolton, Murray T. Brown, Declan C. Schroeder

**Affiliations:** 1 Marine Biological Association of the UK, Citadel Hill, Plymouth, Devon PL1 2PB, UK; deanandrewmckeown@gmail.com (D.A.M.); Joanna.schroeder.uk@gmail.com (J.L.S.); kimberleee@talk21.com (K.S.); 2 School of Biological and Marine Sciences, University of Plymouth, Plymouth, Devon PL4 8AA, UK; M.T.Brown@plymouth.ac.uk; 3 Bezhin Rosko, 40 Rue des Pêcheurs, F-29250 Santec, France; akirapeters@gmail.com; 4 Laboratory of Aquatic Environmental Research, Centre of Advanced Studies, University of Playa Ancha, Viña del Mar 581782, Chile; claudio.saez@upla.cl; 5 Lab of Plant Growth Analysis, Ghent University Global Campus, 119, Songdomunwha-ro, Yeonsu-gu, Incheon 21985, Korea; jihae.park@ghent.ac.kr; 6 Department of Agriculture, Forestry and Fisheries, Private bag X2, Vlaeberg 8018, South Africa; Mark.Rothman@uct.ac.za; 7 Department of Biological Sciences and Marine Research Institute, University of Cape Town, Cape Town 7701, South Africa; john.bolton@uct.ac.za; 8 School of Biological Sciences, University of Reading, Reading RG6 6LA, UK; 9 Veterinary Population Medicine, 225 Veterinary Medical Center, 1365 Gortner Avenue, St Paul, MN 55108, USA

**Keywords:** phaeovirus, *Phycodnaviridae*, Ectocarpales, kelp, NCLDV, prevalence, phylogeny, MCP, latency

## Abstract

Two sister orders of the brown macroalgae (class Phaeophyceae), the morphologically complex Laminariales (commonly referred to as kelp) and the morphologically simple Ectocarpales are natural hosts for the dsDNA phaeoviruses (family *Phycodnaviridae*) that persist as proviruses in the genomes of their hosts. We have previously shown that the major capsid protein (MCP) and DNA polymerase concatenated gene phylogeny splits phaeoviruses into two subgroups, A and B (both infecting Ectocarpales), while MCP-based phylogeny suggests that the kelp phaeoviruses form a distinct third subgroup C. Here we used MCP to better understand the host range of phaeoviruses by screening a further 96 and 909 samples representing 11 and 3 species of kelp and Ectocarpales, respectively. Sporophyte kelp samples were collected from their various natural coastal habitats spanning five continents: Africa, Asia, Australia, Europe, and South America. Our phylogenetic analyses showed that while most of the kelp phaeoviruses, including one from *Macrocystis* *pyrifera*, belonged to the previously designated subgroup C, new lineages of *Phaeovirus* in 3 kelp species, *Ecklonia maxima*, *Ecklonia radiata*, *Undaria pinnatifida*, grouped instead with subgroup A. In addition, we observed a prevalence of 26% and 63% in kelp and Ectocarpales, respectively. Although not common, multiple phaeoviral infections per individual were observed, with the Ectocarpales having both intra- and inter-subgroup phaeoviral infections. Only intra-subgroup phaeoviral infections were observed in kelp. Furthermore, prevalence of phaeoviral infections within the Ectocarpales is also linked to their exposure to waves. We conclude that phaeoviral infection is a widely occurring phenomenon in both lineages, and that phaeoviruses have diversified with their hosts at least since the divergence of the Laminariales and Ectocarpales.

## 1. Introduction

The brown algae (class Phaeophyceae, Stramenopila) are mostly marine macroalgae which belong in the Stramenopila Alveolata Rhizaria (SAR) clade [1] and have evolved complex multicellularity independently from terrestrial plants, red and green algae, animals, and fungi [2]. The independent nature of brown algal evolution has given rise to unique photosynthetic and metabolic features [3]. Two notable brown algal orders, the Ectocarpales and Laminariales (kelp), that share a common branch in multiple gene-based phylogenies, diverged from an Ectocarpales-Laminariales ancestor during the lower Cretaceous period circa 90.5 Ma [4]. The Ectocarpales species *Ectocarpus siliculosus* is an important model organism to investigate mechanisms in cellular and developmental biology, and comparative genomics [2,5].

Globally distributed, both orders are important primary producers, although there is more information about the ecological and economic significance of kelp. Ectocarpales are typically small, fouling, and short-lived macroalgae, while kelp are large, perennial macroalgae which form forests that dominate temperate and subpolar rocky coastlines, from the lower intertidal to the subtidal zones [6,7,8], and occurring in the tropics where sea temperatures are cool enough [9]. Kelp form some of the most productive and structurally complex ecosystems in the world [10,11], which in turn support diverse marine communities [12,13,14,15,16,17,18], influence water movement and coastal erosion rates [19,20,21], export carbon to ecosystems ranging from land to the deep sea [22,23], contribute to marine sediment carbon stores [24], transport climate-altering iodine between the seawater and the atmosphere [25,26,27], and support microbial nitrogen fixation [28,29]. Furthermore, kelp ecosystems provide socioeconomic benefits including fisheries, tourism, coastal protection and environmental remediation [30,31], and cultural heritage [32,33,34]. Humans are increasingly relying on cultivated kelp species for products including food, fertilizer, and industrial chemicals [35] and more recently as sources of renewable bioenergy, and medical applications [36,37,38]. As a result, global kelp aquaculture production has increased 2.3 times since 2000 [39]. Currently, 86% of kelp are cultured in China, mostly the species *Saccharina japonica* and *Undaria pinnatifida* [39]. However, kelp aquaculture is expanding globally [35], such as with *Saccharina* and *Laminaria* spp. in Europe [40,41] and *Macrocystis pyrifera* in Chile [42].

Kelp ecosystems and aquaculture are increasingly coming under threat from human impacts [8,43]. The main threats identified arise from global climate change, chemical and biological pollution, overgrazing, overexploitation, and coastal development, [6,8,44]. It is widely accepted that climate change may alter interactions between macroalgae and pathogens, leading to novel and more virulent disease [45,46,47]. This may heighten the threat of disease to macroalgal aquaculture, which is already experiencing losses to other cultivated species from a range of little understood diseases [48,49].

In general, the viruses of macroalgae are poorly understood [50,51], with the exception of Ectocarpus siliculosus virus 1, genus *Phaeovirus* in the family *Phycodnaviridae* [52,53]. Nine viruses are currently assigned to the genus *Phaeovirus* [54]. The phaeoviruses host range include multiple species within the Ectocarpales and kelp lineages [52,55], but the biology and ecology of kelp phaeoviruses is largely unknown. Phaeoviruses employ a unique latent infection strategy, which begins with the virus infecting the wall-less, free-swimming reproductive algal cells (spores and gametes). The phaeoviral genome is then integrated into the host genome [56]. As the host develops into a mature macroalga, every cell inherits a copy of the phaeoviral genome via mitosis [57,58]. The genome remains latent except in the host reproductive organs (sporangia and gametangia), which become filled with virus particles when the virus is induced [59,60]. In addition to infection by virus particles, phaeoviruses are vertically transmitted by inheritance of the latent phaeoviral genome.

Phaeoviruses are the only known members of the family *Phycodnaviridae* capable of infecting multiple host families [61,62,63]. Other features of phaeoviruses include multiple infections of an individual host [50] and many genes which are unusual for a virus such as polysaccharide metabolism and potassium ion channels [52,64]. The three sequenced phaeoviral genomes have the largest size range of all identified *Phycodnaviridae* and highly divergent genes and structures (EsV-1, 336 kb genome; [52], *Feldmannia irregularis* virus 1, FirrV-1 192 kb genome; [65], *Feldmannia* species virus 158, FsV-158, 155 kb; [53]). Based on concatenated phylogeny of DNA polymerase and major capsid protein (MCP), Ectocarpales phaeoviruses are split into 2 subgroups: subgroup A consisting of one virus genotype, which infects *Ectocarpus*, *Pylaiella*, *Myriotrichia*, and *Hincksia*, and subgroup B, which consists of multiple viral genotypes and infects only *Feldmannia*. The genomes of subgroup B are smaller (from 240–336 kb in A to 155–220 kb in B) and they have lost a DNA proofreading gene, allowing the subgroup B phaeoviruses to exploit a more acute infection strategy, whereas subgroup A viruses have retained a more persistent strategy [66]. The *Phaeovirus* subgroup C has been defined based solely on the MCP found in the kelp species *Laminaria digitata* (Hudson) J.V. Lamoroux, *Laminaria hyperborea* (Gunnerus) Foslie, and *Saccharina latissima* (Linnaeus) C.E. Lane, C. Mayes, Druehl & G.W. Saunders) [55].

Using PCR, 40–100% of *Ectocarpus* individuals have been shown to be infected by phaeoviruses [67,68], and 35% of kelp individuals collected from European waters are infected by phaeoviruses [55]. The only other reports of viruses in kelp are virus-like particles in *Ecklonia radiata* [69], phaeoviral MCPs integrated in the genome of *Saccharina japonica* [70,71], and a viral metagenome from *Ecklonia radiata* [72].

To improve our understanding of viruses in the biology and ecology of kelp, a key first step is to investigate the geographical and host range of kelp phaeoviruses. Moreover, understanding the interactions between phaeoviruses, their Ectocarpales hosts, and the environment is also lacking. To address both issues, we screened kelp and Ectocarpales samples from Africa, Asia, Australia, Europe, and South America. We present a summary of the broad prevalence of phaeoviruses in these 2 major orders of brown algae including previous phaeoviral PCR screen data [55,67,68]. We also describe the interactions between phaeoviral infections, Ectocarpales host species, and wave exposure, and finally the phylogeny of novel phaeoviral MCPs found in the kelp species *E. maxima*, *E. radiata*, *M. pyrifera*, and *U. pinnatifida*.

## 2. Materials and Methods

### 2.1. Sampling and DNA Extraction

**Figure 1:**
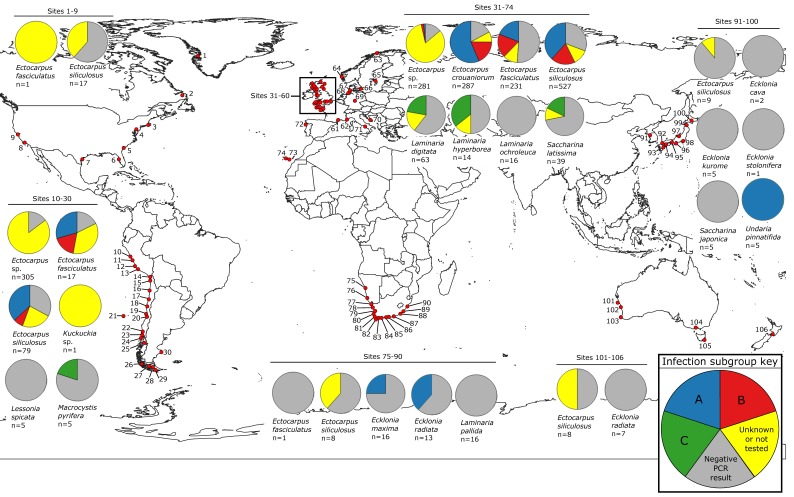
World map of phaeoviral infection prevalence in kelps and Ectocarpales. Red points are sites. Sample sizes are labelled per species (*n*) and pie charts show viral prevalence and subgroup per species at given site range. See Figure S1 for map of sites 31–60. See Table S1 for site key and full sample details.

Epiphyte-free, clean meristematic tissue was cut from kelp sporophytes (diploid) and stored in silica gel. 10–20 mg dry weight of sporophyte material was frozen in liquid nitrogen and homogenized with pestle and mortar. This was followed by DNA extraction with either a NucleoSpin^®^ Plant II (Machery-Nagel, Düren, Germany) kit or a combination of the CTAB and SDS methods [73]. The DNA samples provided [74,75] were extracted using this CTAB-SDS method. Once sufficient biomass of the cultured *Ectocarpus* spp. was obtained [76], DNA was extracted with the NucleoSpin^®^ Plant II (Machery-Nagel) kit. The species sampled comprised of 909 (3 species) of Ectocarpales from 39 sites (3 countries; Figure 1; Figure S1): *Ectocarpus crouaniorum* Thuret in Le Jolis (from high- to mid-intertidal), *Ectocarpus siliculosus* (Dillwyn, Virginia) Lyngbye (from mid-intertidal to subtidal), and *Ectocarpus fasciculatus* Harvey (from low intertidal to subtidal; [77,78]), and 96 (11 species) of Laminariales (kelp) from 26 sites (8 countries; Figure 1): *Ecklonia cava* Kjellman, *Ecklonia kurome* Okamura, *Ecklonia maxima* (Osbeck) Papenfuss, *Ecklonia radiata* (C. Agardh) J. Agardh, *Ecklonia stolonifera* Okamura, *Laminaria ochroleuca* Bachelot de la Pylaie, *Laminaria pallida* Greville, *Lessonia spicata* (Suhr) Santelices, *Macrocystis pyrifera* (Linnaeus) C. Agardh, *Saccharina japonica* (Areschoug) C.E. Lane, C. Mayes, Druehl & G.W. Saunders, and *Undaria pinnatifida* (Harvey, IL, USA) Suringar.

### 2.2. Map of Phaeoviral Prevalence

The map and pie charts (Figure 1, Table S2; Figure S1; Table S1) were constructed using QGIS 3.0.0 [79] and visualized using Inkscape 0.92 [80]. Figure 1 includes this study’s kelp and Ectocarpales data. and also PCR screen data from previous studies comprised of 116 kelp samples from Europe (i.e., 63 *Laminaria digitata*, 14 *Laminaria hyperborea*, 39 *Saccharina latissima*; [55]), 97 Ectocarpales isolates from a broad range of coasts (*Ectocarpus siliculosus*, *Ectocarpus fasciculatus*; [67]), and a further 570 isolates of *Ectocarpus* spp. from the North Atlantic and South Pacific ([68]; Figure 1, Table S2; Figure S1, Table S1).

### 2.3. PCR, Sequencing, Real-Time PCR and High Resolution Melt (HRM) Analysis

The MCP primers used were designed based on a consensus of EsV-1, FirrV-1, FsV-158, and the *E. siliculosus* genome provirus (Figure S2; [66]). The MCP primer sequences were: forward primer CVGCGTACTGGGTGAACGC and reverse primer AGTACTTGTTGAACCAGAACGG. All PCRs were performed using Promega Gotaq^®^ Flexi DNA polymerase kit according to the manufacturer’s instructions (Promega, Madison, WI, USA), with the addition of 1 µL of 0.8 mg/mL bovine serum albumin (BSA) per 25 µL reaction. PCR conditions were as follows: Initial extension of 95 °C for 5 min, then 40 cycles of 95 °C for 1 min (step 1), 55 °C for 30 s (step 2), and 72 °C for 30 sec (step 3), and a final extension of 72 °C for 10 min. All PCR products were Sanger sequenced by Source Bioscience (Nottingham, UK, accessions in Table S1). Real-Time PCR (qPCR) and High Resolution Melt (HRM; [81]) point analysis were carried out using the SensiMix™ HRM kit (Bioline, London, UK) on Ectocarpales samples (Table S1). The raw melting temperatures were calibrated by applying the correction factor from reference clones [66] and genomic DNA. Each corrected melting temperature was assigned to a viral subgroup using posterior group probabilities. The protocol was used according to the manufacturer’s instructions, but decreasing the volumes accordingly to a final mix volume of 10 μL for each reaction, using 0.4 μL 10 μM primers and 0.7 μL 50 mM MgCl_2_ per reaction. Reactions were set up with a CAA1200™ automated liquid handling robot (Corbett Life Science, Sydney, Australia). PCR and melt conditions consisted of a 10 min initial denaturation and enzyme activation step at 95 °C, followed by 40 cycles of 15 s at 95 °C, 10 s at 55 °C and 10 s at 72 °C. To obtain the melting curve, the temperature was ramped from 75 °C to 90 °C, increasing in 0.1 °C per step, with a 90 s wait for pre-melt on step one and 5 s for each subsequent step. Reactions were removed from the cycling conditions in the exponential phase of amplification to allow more reliable calculations of the melt temperature. Peaks lower than the negative controls were considered false negatives. 

### 2.4. Association Analyses

Various association analyses (Table 1) were carried out to determine whether any relationships existed between viral infection rate, viral subgroup, species and degree of wave exposure as defined by Akira Peters (pers. comms.). Sites with enclosed bays were defined as wave-sheltered; sites outside enclosed bays were defined as wave-exposed. Only Ectocarpales were included in these analyses, as the kelp sample size was too small. A Chi-squared (χ^2^) test was used to determine whether the number of infections was the same between exposed and sheltered environments. The χ^2^ test was calculated as: $x^{2}=\sum \frac{{\left(\mathrm{observed}-\mathrm{expected}\right)}^{2}}{\mathrm{expected}}$. A logistic regression model was used for the dichotomous outcome of infected (1) or not infected (0). In the analysis to compare the rates of group A and B infection in the infected isolates, the response variable was 1 if a group virus was present, and 0 otherwise. The logistic analysis was calculated as: $log^{e}\mathrm{Odds}\;\mathrm{of}\;\mathrm{infection}=log^{e}\left(\frac{P\;\left(\mathrm{Infection}\right)}{1-P\;\left(\mathrm{Infection}\right)}\right)$, as a linear function of the dependent variables. Since the probability (p) takes a value between 0 and 1, the log of the odds takes values between –infinity and infinity. This allowed this quantity to be modelled as a linear function of the dependent variables. As a result, probabilities (*p*) for different variables are not compared, but rather the odds are via computation of the odds ratio:$\;\mathrm{Odds}\;\mathrm{ratio}\;\mathrm{Group}1:\mathrm{Group}2=\frac{\mathrm{Odds}\;\mathrm{in}\;\mathrm{Group}1}{\mathrm{Odds}\;\mathrm{in}\;\mathrm{Group}\;2}$. The hypothesis test to determine whether the odds of infection are different between two groups tested whether the odds ratio is significantly different from 1. The Cochran-Mantel-Haenszel test was used to determine whether viral infection rate differed between wave-exposed and -sheltered shore environments within each host species. A common odds ratio for wave-exposed and -sheltered shore environments was fitted for each host species group and permutations were used to determine whether this odds ratio is significantly different from 1. The p-value of the test is estimated to be the proportion of permutated odds ratio statistics that exceeded the observed odds ratio. The Mantel-Haenszel (exact) test was carried out on wave exposure versus presence/absence of virus infection to estimate the common odds ratio of the probability of virus infection occurring in wave-exposed or -sheltered environments, and whether this ratio was independent of host species.

### 2.5. Phylogenetic Analysis and Tree Construction

**Figure 2:**
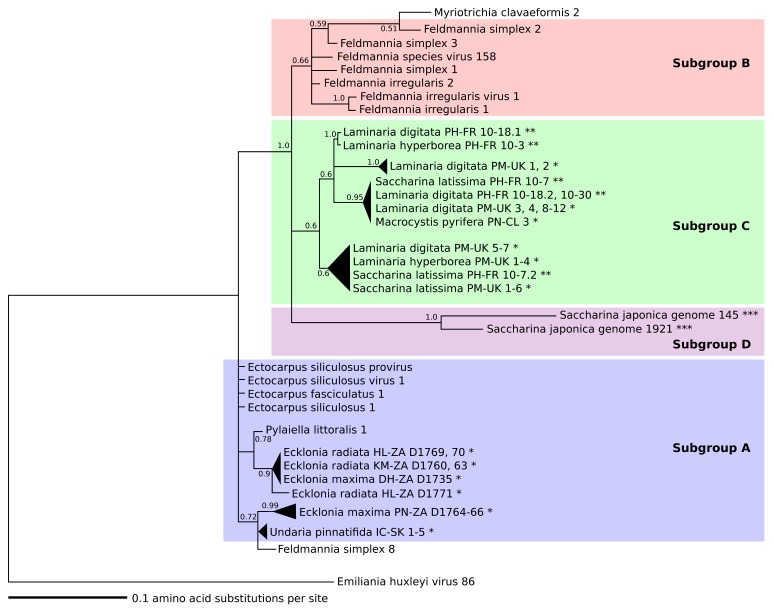
Phylogeny of partial *Phaeovirus* MCP amplified by PCR from Ectocarpales and kelps. Subgroups A (**blue**) and B (**red**) are labelled as previously defined [66], subgroup C (**green**) by [55], and subgroup D (**purple**). Scale units are the number of amino acid substitutions per site. Triangles are collapsed branches. Node values are Bayesian inference proportions. Root is the out-group Emiliania huxleyi virus 86. Kelp life history stages are labelled sporophyte (*), gametophyte (**), kelp gamete (***). Country codes; Chile (CL), France (FR), South Korea (SK), United Kingdom (UK), South Africa (ZA). Sites codes; De Hoop (DH), Hluleka (HL), Incheon (IC), Kei Mouth (KM), Perharidy (PH), Piedras Negras (PN), Plymouth (PM), Port Nolloth (PN). See Figure S2 (*S. japonica*) and Table S1 for accession numbers and sample details, and Figure 3 for alignment.

**Figure 3:**
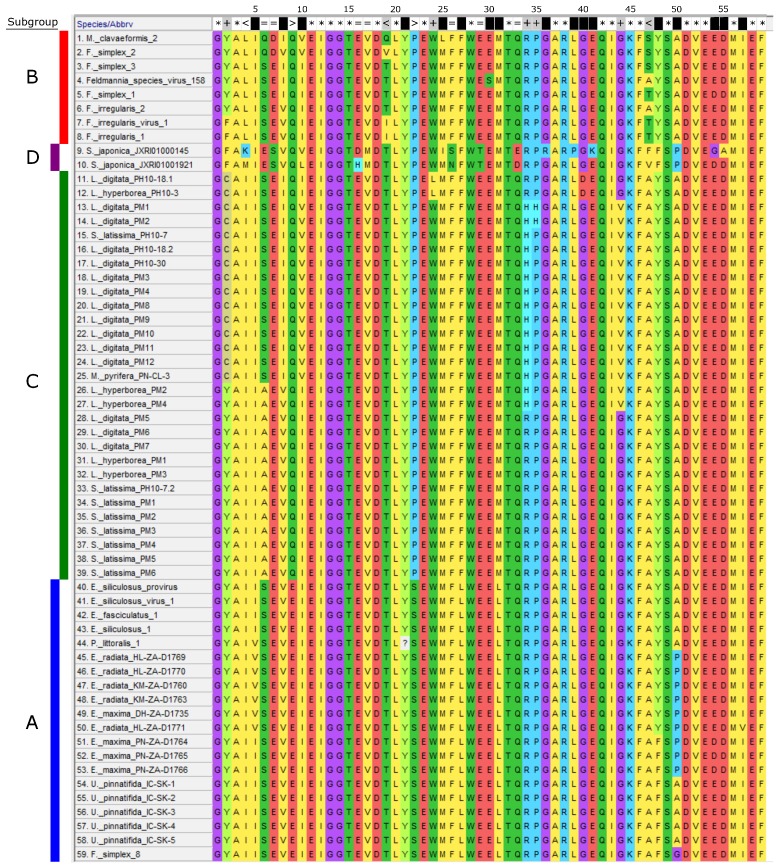
Multiple amino acid sequence alignment of *Phaeovirus* MCP fragments used in phylogenetic analysis. Colours represent the amino acids as labelled. This alignment was the basis of Figure 2. Sites conserved across all subgroups are labelled (*). Sites conserved within subgroups are labelled at the top for subgroups B (<), A (>), D (=), C (+) and level of conservation within subgroup; none (**black**), partial (**grey**), 100% (**white**).

**Figure 4:**
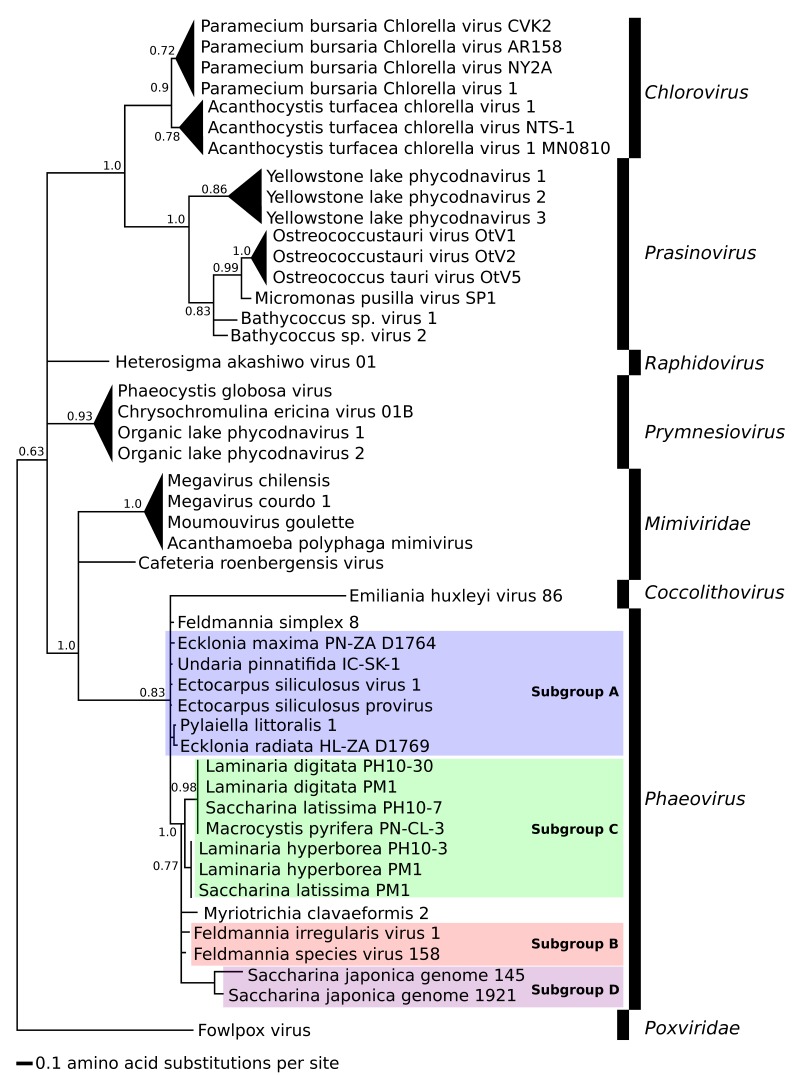
Phylogeny of partial *Phaeovirus* MCP amplified by PCR from kelps. These sequences were aligned with other *Phycodnaviridae* (*Coccolithovirus*, *Phaeovirus*, *Raphidovirus*, *Prymnesiovirus*, *Prasinovirus*, *Chlorovirus*) and *Mimiviridae*. Subgroups A (**blue**) and B (**red**) are labelled as previously defined [66], subgroup C (**green**) as by [55], and subgroup D (**purple**). Scale units are the number of amino acid substitutions per site. Triangles are collapsed branches. Node values are Bayesian inference proportions. Root is the out-group Fowlpox virus. See Figure S2 (*S. japonica*) and Tables S1 and S3 for accession numbers and sample details.

For phylogenetic analysis we used the protein sequences translated from the *mcp* gene fragments amplified from kelp and Ectocarpales (Figure 2 and Figure 3) and from the *mcp* genes of known *Phycodnaviridae* and *Mimiviridae* (Figure 4, see Tables S1 and S3 for all accession numbers). Only the conserved MCP region aligned with the MCP fragment found in kelp and Ectocarpales was used to construct Figure 4. The additional sequences in Figure 4 were obtained using the GenBank blastp algorithm and selecting the sequence with the highest homology to phaeoviral MCP within each available genome of *Phycodnaviridae* and *Mimiviridae*. All translated amino acid sequences were aligned using MUSCLE using MEGA7 [82]. Bayesian inference analysis was performed using MrBayes v3.2 [83], stopping the analysis once the number of generations was over 300,000 and once the posterior probabilities no longer changed with each generation. Trees were visualized using Inkscape 0.92 [80] and Dendroscope 3 [84] and rooted using MCP from the poxvirus *Fowlpox virus* (*Poxviridae*). The *Phaeovirus* MCPs reported in the genome of S. *japonica* [70] were compared to MCPs from other kelp species. *Saccharina japonica* MCPs were found with the GenBank blastn algorithm searching the *S. japonica* genome using *MCP* genes from EsV-1, FsV-158, and FirrV-1. MCP ORFs were identified from the *S. japonica* genome scaffolds using Artemis [85]. MCPs from phaeoviral genomes were aligned with MCPs from *S. japonica* and the MCP primers (Figure S2) to examine their homology.

## 3. Results

### 3.1. Phaeoviral Prevalence in the Laminariales

PCR detected a phaeoviral *mcp* gene fragment in 4 of 11 kelp species tested. This amplified *mcp* gene fragment was 181 bp to 214 bp. There was a positive result in 15.6% of the kelp sporophytes studied (15 out of 96; Figure 1, Table S2, Table S1). Phaeoviral MCP was found in 25% of *E. maxima* (4 out of 16, South Africa), 25% of *E. radiata* (5 out of 20, South Africa), 20% of *M. pyrifera* (1 out of 5, Chile), and 100% of *U. pinnatifida* (5 out of 5, South Korea). Phaeoviral MCP was not found in *E. cava* (out of 2, Japan), *E. kurome* (out of 5, Japan), *E. stolonifera* (out of 1, Japan), *L. ochroleuca* (out of 16, UK and Portugal), *L. pallida* (out of 16, South Africa, Namibia), *L. spicata* (out of 5, Chile), and *S. japonica* (out of 5, South Korea). Including previously published data, the overall phaeoviral infection rate of kelp was 26% (56 out of 212 individual sporophytes).

### 3.2. Phylogeny of Phaeoviruses Including Novel Kelp MCPs

Subgroup B viruses were grouped together, but with low support (0.66; Figure 2). In a previous study, concatenated MCP and DNA polymerase phylogeny placed *M. clavaeformis* 2 in subgroup A [66], but this study’s analysis placed it in subgroup B (Figure 2). Ectocarpales subgroup A viruses were closely related, but not within a supported node (Figure 2). Phaeoviral MCP from *U. pinnatifida*, *E. maxima*, and *E. radiata* fell within subgroup A (0.72 and 0.78; Figure 2). *P. littoralis* 1 was most similar to the kelp subgroup A viruses (0.78; Figure 2). *F. simplex* 8 was previously placed by concatenated MCP and DNA polymerase phylogeny as an intermediate between subgroups A and B [66], but this study’s analysis placed it with the subgroup A kelp phaeoviruses (0.72; Figure 2). MCP from *L. digitata*, *L. hyperborea*, *S. latissima*, and *M. pyrifera* were assigned to subgroup C with low support (0.6) and were more closely related to subgroup B than A (1.0; Figure 2). MCPs from the genome of *S. japonica* were divergent from subgroups B and C, and were defined as subgroup D (1.0; Figure 2). Out of 59 amino acids, the subgroup A was distinguished from subgroups B, C, and D by 2 amino acids (100% conserved sites 9 and 22; Figure 3). Subgroup B had 3 amino acids different from the other subgroups (100% conserved site 4; partially conserved sites 19 and 47; Figure 3). Subgroup C was distinguished from the other subgroups by 5 amino acids (partially conserved sites 2, 24, 34, 35, and 44; Figure 3). Subgroup D was the most divergent, with 7 amino acids different from the other subgroups (100% conserved sites 6, 7, 16, 17, 26, 29, 33; Figure 3).

### 3.3. Phylogeny of Phaeoviruses Including MCP of Other Phycodnaviruses and Mimiviruses

Phylogeny based on the MCP region orthologous to the conserved 59 amino acid MCP fragment found in kelp could distinguish *Phycodnaviridae* genera and *Mimiviridae* (Figure 4). All phaeoviral MCPs fall within the *Phaeovirus* genus, including the MCPs from *S. japonica*. Most phycodnavirus members are grouped together into their previously defined genera with high support (0.9 *Chlorovirus*, 1.0 *Prasinovirus*, 0.93 *Prymnesiovirus*), and members of *Mimiviridae* are grouped together (1.0) except *Cafeteria roenbergensis virus*. The other exception was the grouping of *Coccolithovirus* and *Phaeovirus* together (0.83), but with large evolutionary distance between these 2 genera.

### 3.4. Phaeoviral MCP from S. japonica

Both JXRI01001921 and JXRI01000145 MCPs were included in this study’s phylogeny (Figure 2 and Figure 4) as they contained the conserved MCP region found in Ectocarpales and kelp (Figure 3, Figure S2). The JXRI01000271 MCP was too short to be included in the phylogenetic analysis. The 3 phaeoviral MCP orthologs in the *S. japonica* genome had distinct structures. The JXRI01001921 MCP was a 458 amino acid ORF, with an MCP primer binding site which mismatched our primers by 3 bases (forward primer) and 2 bases (reverse primer). The MCP primer binding sites of EsV-1, FirrV-1, and FsV-158 matched every base of both primers (Figure S2). JXRI01000145 MCP was a 439 amino acid non-ORF containing 7 stop codons, with an MCP primer binding site which mismatched our primers by 2 bases (forward primer) and 2 bases (reverse primer; Figure S2). JXRI01000271 MCP was 47 amino acids within a 263 amino acid ORF, with an MCP primer binding site which mismatched our primers by 4 bases (forward primer) and did not contain the reverse primer binding site.

### 3.5. Phaeoviral Prevalence in Ectocarpales 

HRM qPCR screening found the MCP phaeoviral DNA fragment in all 3 Ectocarpales species tested (Figure 1, Table S2, Table 1, Figure S1, Table S1). A positive result was found in 63% of this study’s *Ectocarpus* isolates (186 out of 236 in *E. crouaniorum*, 298 out of 466 in *E. siliculosus*, and 89 out of 207 in *E. fasciculatus*). Comparison of the overall infection numbers between all Ectocarpales species from wave-sheltered and those from wave-exposed environments showed that Ectocarpales from wave-sheltered environments had 3.52 times lower than expected infection rates compared to the wave-exposed environments (with 95% confidence interval 2.25–5.59). In addition, Cochran-Mantel-Haenzel exact test yielded a *p*-value = 2.641 × 10^−9^, thus rejecting the probability that viral infection and wave exposure are not associated. Using a Chi-squared test of homogeneity, we rejected the null hypothesis of equal distribution of Ectocarpales species between wave-sheltered and -exposed sites (*p* = 3.57 × 10^−45^).

With the following exceptions, all infection types (subgroup A only, subgroup B only, and both subgroups per individual) were equally likely at wave-exposed or -sheltered sites (Table 1(a–c)). Increased likelihood of infection was observed in: subgroup A infection of *E. crouaniorum* at wave-sheltered sites (Table 1(b)) and subgroup B infection of *E. fasciculatus* at wave-exposed shores (Table 1(c)). A Cochran-Mantel-Haenszel test for homogeneity across wave-sheltered and -exposed sites with respect to species based on Table 1 led to rejection of the null hypothesis (*p* = 8.583 × 10^−11^), i.e., both species and wave exposure influenced the type of virus infection.

The data of virus positive isolates from Table 1 was reduced to a binary form with those only infected by subgroup A viruses represented by 0 and those infected by subgroup B, or both A and B, as 1. Fitting a logistic regression model to this binary data showed that the odds of subgroup B infection were 2.25 times more likely (*p* = 0.00632, 95% confidence interval 1.26–4.04) in infected isolates of *E. fasciculatus* than in either *E. crouaniorum* or *E. siliculosus*. We also found the odds of multiple viral infections in Ectocarpales in sheltered environments was 2.53 times that of wave-exposed environments (logistic regression odds ratio estimate was 2.53 with 95% Confidence intervals 1.53–4.80 and *p*-value = 0.000296). Including previously published data (680 individuals), the overall phaeoviral infection rate of Ectocarpales was 71% (1125 out of 1589 individuals).

## 4. Discussion

Including data from our previous study [55], 26% of kelp individuals were positive for phaeoviral MCP. Novel phaeoviral MCPs were found in 4 species of kelp (Figure 1, Table S2; *M. pyrifera*, *E. maxima*, *E. radiata*, and *U. pinnatifida*). Including *S. japonica* [70], *L. digitata*, *L. hyperborea*, and *S. latissima* [55], this expands the *Phaeovirus* host range to 8 kelp species in 5 genera and includes the most species-rich genera of the Laminariales, which contain 44% of all kelp species (63 out of 143 Laminariales species; *Laminaria*, *Saccharina*, *Ecklonia* [86]). It is, therefore, reasonable to expect phaeoviral infection to be present throughout the entire kelp order. Basal kelp taxa such as the family Chordaceae and *Aureophycus aleuticus* [4] should be assessed to test whether phaeoviral infection is present throughout the Laminariales. Furthermore, kelp phaeoviruses are geographically widespread, being present in kelp species from Europe (UK, France), South America (Chile), Asia (South Korea), and Africa (South Africa). Kelp phaeoviral subgroups are likewise geographically widespread, with subgroup C being present in Europe and South America (Figure 1) and subgroup A being present in Africa and Asia (Figure 1).

The subgroup A and B viruses (Figure 2) were not grouped as previously defined [66] and showed the MCP fragment alone to be an unreliable marker for assigning viral subgroups. Phylogenetic analysis including other core viral genes would more reliably reflect the evolutionary relationships of kelp phaeoviruses. Compared to equivalent MCP regions from members of *Phycodnaviridae* and *Mimiviridae*, the MCP fragment from kelp (Figure 3) showed mostly appropriate phylogeny of NCLDVs [87,88], with good support for the assignment of these kelp viruses to *Phaeovirus* (Figure 4).

We hypothesize that *Laminaria*, *Saccharina*, and *Macrocystis* phaeoviruses (subgroup C) have smaller genomes and broader host range (as they are close to subgroup B) than *Undaria* and *Ecklonia* viruses (subgroup A). Subgroup C may be a viral lineage with a host range of at least 3 kelp genera (*Saccharina*, *Laminaria*, and *Macrocystis*; Figure 2). Subgroup D shares a *Saccharina* host range with subgroup C (Figure 2), suggesting divergent phaeoviruses infecting closely related host species [89]. The extent to which phaeoviruses co-diverge with their kelp hosts is unclear, but could reveal novel understanding of viral evolution, especially regarding the shifts between horizontal (transmission via virus particles) and vertical (transmission via genome integration; may have greater degree co-divergence with host). However, it is worth noting that phylogeny based on multiple core NCLDV genes would more reliably represent the evolutionary relationships of kelp phaeoviruses, but first kelp *Phaeovirus* genomes sequences must be acquired.

These kelp MCPs are only a hint of *Phaeovirus* prevalence and diversity, as the negative MCP PCR results may be due to divergent phaeoviruses with low affinity for our MCP primers, which may help explain the lower infection rate of 26% in kelp versus 63% in Ectocarpales (Figure 1). For example: the absence of MCP in the *S. japonica* samples may have been false negatives, as our primers would not have amplified the phaeoviral MCPs in the *S. japonica* genome (Figure S2; [70,71]). The presence of MCP in the *S. japonica* genome [70], in addition to apparent Mendelian inheritance of phaeoviral MCP in kelp gametophytes [54], suggests kelp phaeoviruses employ a latent infection strategy involving provirus integration into the host genome.

The 63% prevalence of *Phaeovirus* infection in Ectocarpales is within the range of previous approximations of 40 to 100% (Figure 1, Table S2; [67,68]). Ectocarpales from sheltered environments had 3.52 times lower infection rates than those from wave-exposed environments (Table 1; 95% confidence interval 2.25–5.59, Cochran-Mantel-Haenzel *p*-value = 2.641× 10^−9^), which supported the association between viral infection and wave exposure. Among infected Ectocarpales, subgroup B infection is 2.25 times more likely in *E. fasciculatus* than in either *E. crouaniorum* or *E. siliculosus* (logistic regression model; *p*-value = 0.00632, 95% confidence interval 1.26–4.04). This correlates with the subtidal niche of *E. fasciculatus*. We also found that multiple viral infections in Ectocarpales were 2.53 times more likely in wave-sheltered environments compared to wave-exposed environments (logistic regression odds ratio estimate was 2.53 with 95% confidence intervals 1.53–4.80 and *p*-value = 0.000296). We conclude that environmental conditions (wave-exposed or -sheltered) had a strong association with the odds of both single and multiple phaeoviral infections. If the relationship between viral infection and wave exposure is true in general for brown algae, then the location of kelp aquaculture or forests may strongly influence the rate of viral infection.

The brown algae belong to the SAR clade [1], which may be the most diverse of the major eukaryotic lineages [90]. However, brown algae are the only members of the SAR clade which have evolved complex multicellularity [2]. Since phaeoviruses are related to phycodnaviruses which infect unicellular eukaryotes, it follows that comparative genomics of novel phaeoviruses could reveal how phycodnaviruses have adapted to infect multicellular hosts. Furthermore, the widespread and latent phaeoviruses could offer a unique system for exploring the deeper evolutionary relationships of virus and host, as integrated viral sequences (endogenous viral elements; EVEs) which evolve at the rate of the host can be compared to exogenous viruses [91]. For example; to test whether phaeoviral EVE ages correlate with the proposed timing of the diversification of the 4 derived Laminariales families in the North Pacific [92], or how the dynamics of expansion and reduction in phaeoviral EVEs over long evolutionary timescales compare to hypotheses regarding NCLDV genome evolution [93].

Human impacts on kelp ecosystems [6] and aquaculture [43] are expected to threaten the ecological and economic roles of kelp [8,94]. These threats include climate change, pollution, overexploitation, and overgrazing leading to barren grounds [6,8,44,95]. In the future of aquaculture, macroalgae are expected to have reduced performance in warmer, more acidic oceans [43] and experience losses from a range of eukaryotic and bacterial pathogens [45,46,47,48,49,96]. Viruses however, are largely absent from our understanding of macroalgal ecology and performance [50]. We have shown evidence of phaeoviral infection in 5 kelp genera of major ecological and economic importance (*Saccharina*, *Laminaria*, *Macrocystis*, *Undaria*, and *Ecklonia*) and the impact of phaeoviral infection on these genera should be further investigated. This is especially pertinent since cultivation facilitates disease via reduced genetic diversity of domesticated organisms, high stock density, crop to wild disease spread, and favoring horizontal over vertical viral transmission [48,49,96,97].

Another unknown is the role of viruses in the recent range shifts of kelp. These shifts include the spread of the invasive kelp *U. pinnatifida* [98], the pole ward shifts of European kelp involving the displacement of cold water kelp by warm water kelp [99], or the eastward spread of *E. maxima* in South Africa [92]. The outcome of these shifts is impoverished biodiversity and ecosystem services. In terrestrial plants, reduced viral infection can facilitate range shifts [100] and changes in viral infection can favor invasive over native plants [101]. Whether viruses play similar roles in the range shifts of kelp remains to be explored. Since Ectocarpales phaeoviruses are temperature sensitive [60,102] and can reduce rates of host photosynthesis and respiration [102], it should be determined whether phaeoviral infection impacts the biogeographical distributions of kelp.

## 5. Conclusions

In Ectocarpales brown algae, phaeoviral infection is influenced by wave exposure, with implications for the placement of macroalgal aquaculture facilities. We expand the phaeoviral host range to a total of eight kelp species including the most species-rich genera and their geographical range to five continents. These novel MCPs from kelp may represent new members of the genus *Phaeovirus*. Phaeoviral infections may be present in the entire kelp order, a group of ecologically and economically important marine macroalgae. However, we lack the molecular tools to thoroughly study the diversity and evolutionary relationships of kelp phaeoviruses. The impacts of viral infections are a major knowledge gap for kelp conservation and cultivation, despite the potential for viruses to alter the success of kelp under climate change and aquaculture scenarios. The widespread detection of phaeoviruses within species of the Laminariales and the influence of wave exposure on phaeoviral infection with species of the Ectocarpales emphasizes the potential for phaeoviruses to have profound influences on the interactions between humans, environment, and brown macroalgae.

## Figures and Tables

**Table 1:** A three-way contingency table showing the viral subgroups sampled from wave-exposed and -sheltered sites divided by Ectocarpales species. Species: (a) *E. siliculosus*, (b) *E. crouaniorum*, and (c) *E. fasciculatus.* Numbers in parentheses represent the expected values if the viral subgroups were distributed evenly between wave-exposed and -sheltered environments for each algal host species.

Infection Type	Sheltered	Exposed	Total Number of Isolates
(a) *Ectocarpus siliculosus*			
None	169 (162.0)	5 (12)	174
Subgroup A only	139 (147.1)	19 (10.9)	158
Subgroup B only	12 (11.2)	0 (0.8)	12
Both	72 (71.7)	5 (5.3)	77
Total number of isolates	392	29	421
(b) *Ectocarpus crouaniorum*			
None	43 (27.3)	7 (22.7)	50
Subgroup A only	46 (63.9)	71 (53.1)	117
Subgroup B only	4 (3.8)	3 (3.2)	7
Both	25 (23.0)	17 (19.0)	42
Total number of isolates	118	98	216
(c) *Ectocarpus fasciculatus*			
None	69 (58.8)	46 (56.2)	115
Subgroup A only	13 (14.3)	15 (13.7)	28
Subgroup B only	1 (9.7)	18 (9.3)	19
Both	9 (9.2)	9 (8.8)	18
Total number of isolates	92	88	180

## Supplementary Materials

The following are available online at http://www.mdpi.com/1999-4915/10/8/410/s1. Figure S1: Map of collection sites 31–60 of kelp and Ectocarpales infected with phaeoviruses, Figure S2: Multiple nucleotide sequence alignment of phaeoviral MCP primers, phaeoviral MCP, and *S. japonica* MCP, Table S1: Data of phaeoviral infections of Ectocarpales and kelp, Table S2: Summary of phaeoviral infections detected with PCR in kelp sporophytes, kelp gametophytes, and Ectocarpales, and Table S3: GenBank accession numbers of capsid protein sequences from other *Phycodnaviridae* and *Mimiviridae*.
