# Supplementary material for: Phaeoviral Infections Are Present in Macrocystis, Ecklonia and Undaria (Laminariales) and Are Influenced by Wave Exposure in Ectocarpales
Source: Viruses. 2018 Aug 5;10(8):410. doi: 10.3390/v10080410 (PMC6116031; doi:10.3390/v10080410)
Supplement: Supplementary file 1 [file viruses-10-00410-s001.pdf]

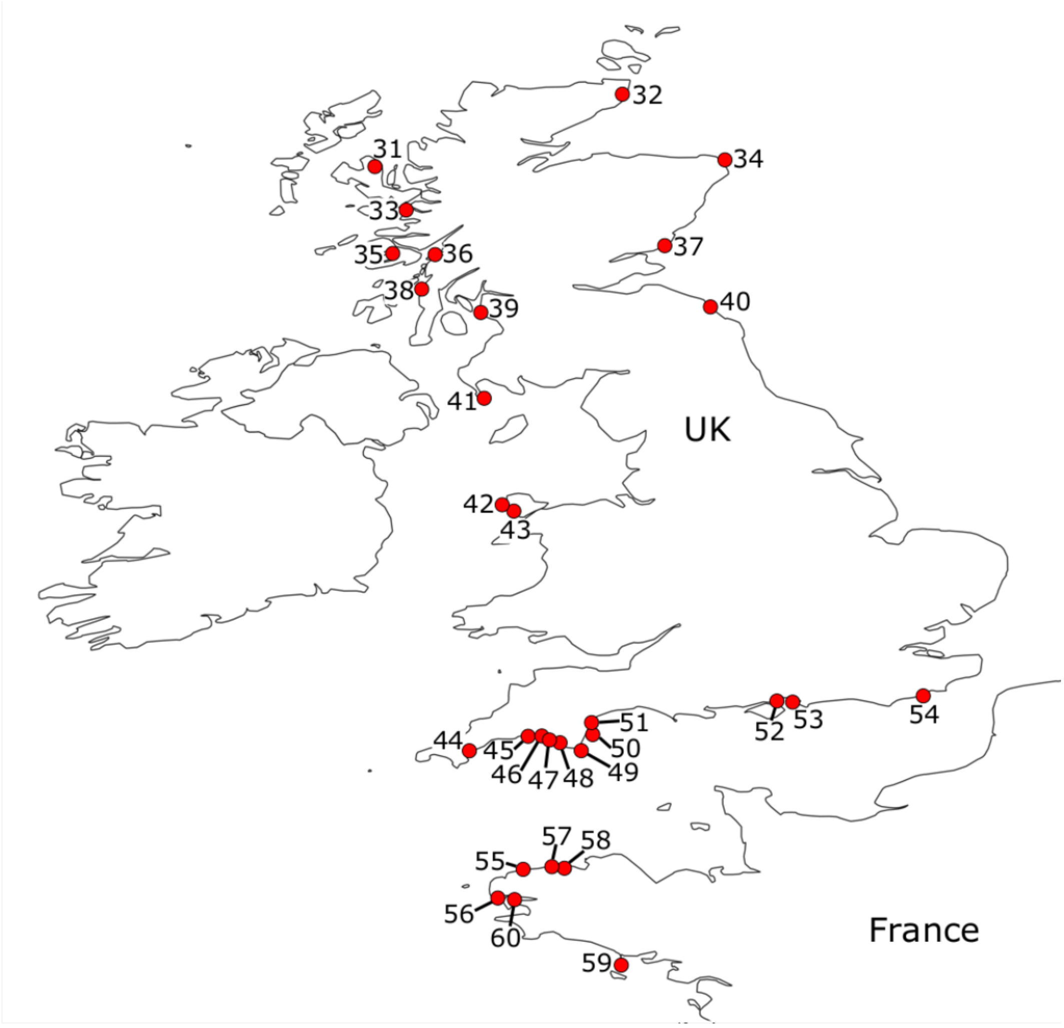

Figure S1. Map of collection sites 31-60 of Laminariales and Ectocarpales infected with phaeoviruses. Red points are sites. Sample size=n. See Table S1 for full sample details. Site key: 31 Isle of Skye, 32 Wick, 33 Mallaig, 34 Rattray Head, 35 Isle of Mull, 36 Oban, 37 Arbroath, 38 Taynish, 39 Portencross, 40 Berwick, 41 Mull of Galloway, 42 Trearddur, 43 Rhosneigr, 44 Restronguet Point, 45 Whitsand Bay, 46 Plymouth, 47 Wembury, 48 Mothecombe, 49 Start Point, 50 Brixham, 51 Torquay, 52 Gosport, 53 Hayling Island, 54 Pett, France: 55. Traezh Hir, 56 Perharidy, 57 Roscoff, 58 Terenez, 59 Quiberon, 60 Caro.

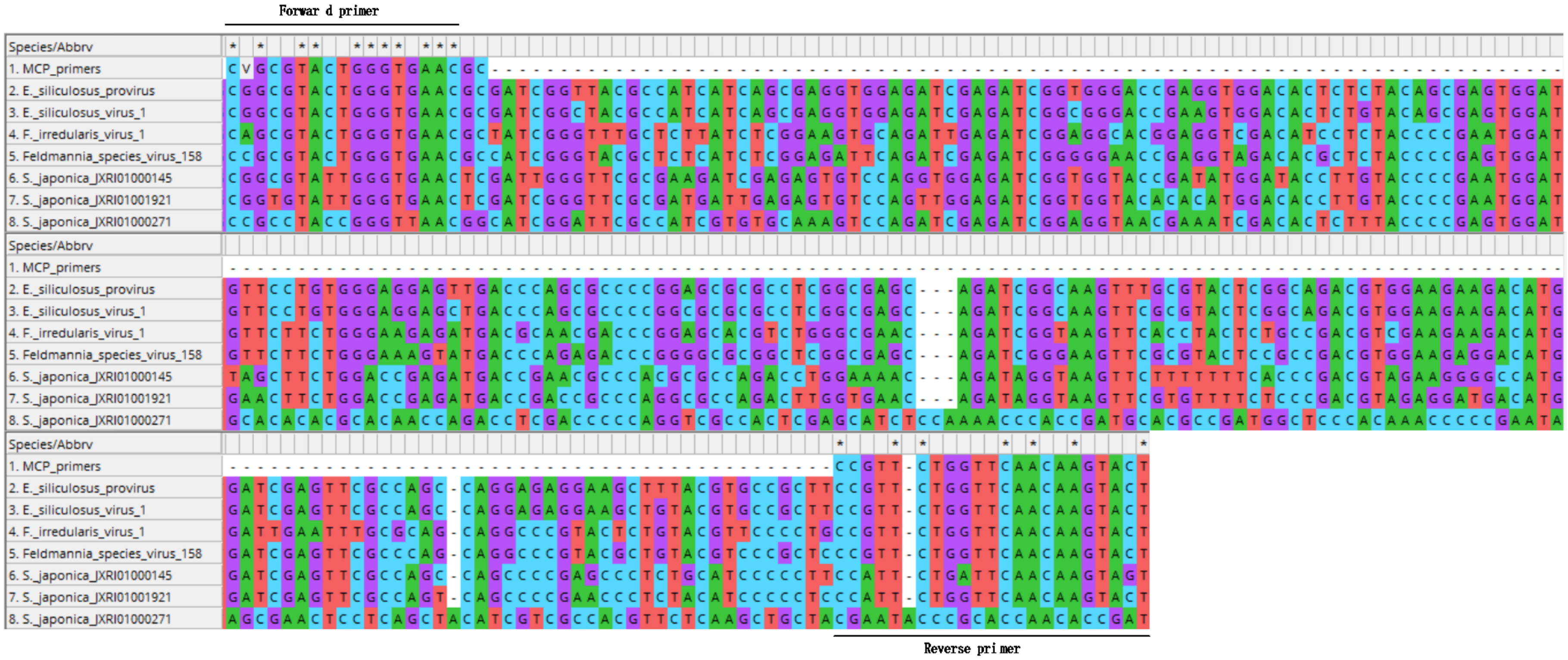

Figure S2. Multiple nucleotide sequence alignment of phaeoviral MCP primers, phaeoviral MCP, and *S. japonica* MCP. Region between the primers is the partial MCP sequence used in phylogenetic analysis (Figure 2). Conserved sites are indicated by asterisks.

Table S1. Data of phaeoviral infections of Ectocarpales and Laminariales. Includes data from this study, McKeown et al. (2017), Müller et al. (2000), and Sengco et al. (1996). Unknown=phaeoviral infection of unknown subgroup, None = no virus infection, A = subgroup A, B = subgroup B, C = subgroup C, * = unknown subgroup. Multiple subgroups are indicated in samples with multiple infections. All infections detected by PCR or qPCR and accession numbers given for sequenced PCR products. MCP = major capsid protein, Gp-1 = viral capsid protein gp1, - = not tested, - = negative result, + = positive result. NA = not available, S = sporophyte, G = gametophyte, qPCR = real time quantitative PCR. Site key: 1 Greenland. Canada: 2 Newfoundland. USA: 3 Penikese, 4 Wilmington, 5 Beaufort, 6 Tampa, 7 Port Aransas, 8 Santa Barbara, 9 San Francisco. Peru: 10 Peru, 11 Ancón, 12 Mednieta, 13 Marcona. Chile: 14 Arica, 15 Pisagua, 16 Antofagasta, 17 Bahía Inglesa, 18 Coquimbo, 19 Quintay, 20 Piedras negras, 21 Juan Fernandez, 22 Valdivia, 23 Puerto Montt, 24 Maullin, 25 Puyuguapi, 26 South Chile, 27 Magellan Straits, 28 Tierra del Fuego. Argentina: 29 Ushuaia, 30 Puerto Deseado. UK: 31 Isle of Skye, 32 Wick, 33 Mallaig, 34 Rattray Head, 35 Isle of Mull, 36 Oban, 37 Arbroath, 38 Taynish, 39 Portencross, 40 Berwick, 41 Mull of Galloway, 42 Trearddur, 43 Rhosneigr, 44 Restronguet Point, 45 Whitsand Bay, 46 Plymouth, 47 Wembury, 48 Mothecombe, 49 Start Point, 50 Brixham, 51 Torquay, 52 Gosport, 53 Hayling Island, 54 Pett, 55. France: Traezh Hir, 56 Perharidy, 57 Roscoff, 58 Terenez, 59 Quiberon, 60 Caro, 61 Bouzigues, 62 Villefranche. Norway: 63 Tromso, 64 Bergen. Sweden: 65 Baltic Sea. Denmark: 66 Copenhagen. Germany: 67 Sylt, 68 Helgoland, 69 River Werra. Croatia: 70 Split. Italy: 71 Naples. Portugal: 72 Amorosa. Spain: 73 Canary Islands, 74 Gran Canaria. Namibia: 75 Swakopmund, 76 Lüderitz. South Africa: 77 Port Nolloth, 78 Hondeklip Bay, 79 Doring Bay, 80 Jacob's Bay, 81 Cape Town, 82 Oudekraal, 83 Stanford's Cove, 84 De Hoop, 85 Buffels Bay, 86 Nature's Valley, 87 Port Elizabeth, 88 Kei Mouth, 89 Hluleka, 90 Durban. South Korea: 91 Incheon, 92. Japan: Yamaguchi, 93 Hatozu, 94 Ehime, 95 Kobe, 96 Goza-Shirahama, 97 Itoigawa, 98 Zushi, 99 Oma, 100 Hokkaido. Australia: 101 Abrolhos, 102 Jurien Bay, 103 Hamelin Bay, 104 Victoria, 105 Fortescue. 106 New Zealand.

Species	Life cycle phase	Source, collector	Sample code	Date	Exposure	Virus subgroup	PCR result, accession
Ecklonia cava	S	96, K. Kogame	EcavGS-JP-D1744	NA	NA	None	MCP -
Ecklonia cava	S	98, S. Uwai	EcavZU-JP-D1751	NA	NA	None	MCP -
Ecklonia kurome	S	93, S. Uwai	EkurHZ-JP-D1774	NA	NA	None	MCP -
Ecklonia kurome	S	93, S. Uwai	EkurHZ-JP-D1775	NA	NA	None	MCP -
Ecklonia kurome	S	93, S. Uwai	EkurHZ-JP-D1776	NA	NA	None	MCP -
Ecklonia kurome	S	97, M. Okaji	EkurIG-JP- D1747	NA	NA	None	MCP -
Ecklonia kurome	S	97, M. Okaji	EkurIG-JP-D1748	NA	NA	None	MCP -
Ecklonia maxima	S	84, R. Anderson	EmaxDH-ZA-D1730	NA	NA	None	MCP -
Ecklonia maxima	S	84, R. Anderson	EmaxDH-ZA-D1731	NA	NA	None	MCP -
Ecklonia maxima	S	84, R. Anderson	EmaxDH-ZA-D1732	NA	NA	None	MCP -
Ecklonia maxima	S	84, R. Anderson	EmaxDH-ZA-D1733	NA	NA	None	MCP -
Ecklonia maxima	S	84, R. Anderson	EmaxDH-ZA-D1734	NA	NA	None	MCP -
Ecklonia maxima	S	84, R. Anderson	EmaxDH-ZA-D1735	NA	NA	Unknown	MCP +, MG967362
Ecklonia maxima	S	84, R. Anderson	EmaxDH-ZA-D1738	NA	NA	None	MCP -
Ecklonia maxima	S	79, M. Rothman	EmaxDB-ZA-D1728	NA	NA	None	MCP -
Ecklonia maxima	S	79, M. Rothman	EmaxDB-ZA-D1729	NA	NA	None	MCP -
Ecklonia maxima	S	80, M. Rothman	EmaxJB-ZA-D1757	NA	NA	None	MCP -
Ecklonia maxima	S	82, M. Rothman	EmaxOK-ZA-D1772	NA	NA	None	MCP -
Ecklonia maxima	S	82, M. Rothman	EmaxOK-ZA-D1773	NA	NA	None	MCP -
Ecklonia maxima	S	77, M. Rothman	EmaxPN-ZA-D1764	NA	NA	Unknown	MCP +, MG967368
Ecklonia maxima	S	77, M. Rothman	EmaxPN-ZA-D1765	NA	NA	Unknown	MCP +, MG967369
Ecklonia maxima	S	77, M. Rothman	EmaxPN-ZA-D1766	NA	NA	Unknown	MCP +, MG967370
Ecklonia maxima	S	83, M. Rothman	EmaxSC-ZA-D1767	NA	NA	None	MCP -
Ecklonia radiata	S	101, M. Mohring	EradAB-AU-D1723	NA	NA	None	MCP -
Ecklonia radiata	S	101, M. Mohring	EradAB-AU-D1724	NA	NA	None	MCP -
Ecklonia radiata	S	101, M. Mohring	EradAB-AU-D1725	NA	NA	None	MCP -
Ecklonia radiata	S	105, M. Mohring	EradFS-AU-D1739	NA	NA	None	MCP -
Ecklonia radiata	S	103, M. Mohring	EradHA-AU-D1740	NA	NA	None	MCP -
Ecklonia radiata	S	103, M. Mohring	EradHA-AU-D1741	NA	NA	None	MCP -
Ecklonia radiata	S	102, D. Kemp	EradJU-AU-D1743	NA	NA	None	MCP -
Ecklonia radiata	S	76, A. Plos	EradLU-NA-D1753	NA	NA	None	MCP -
Ecklonia radiata	S	76, A. Plos	EradLU-NA-D1754	NA	NA	None	MCP -
Ecklonia radiata	S	85, D. Kemp	EradBB-ZA-D1726	NA	NA	None	MCP -
Ecklonia radiata	S	85, R. Anderson	EradBB-ZA-D1727	NA	NA	None	MCP -
Ecklonia radiata	S	84, C. Boothroyd	EradDH-ZA-D1736	NA	NA	None	MCP -
Ecklonia radiata	S	84, C. Boothroyd	EradDH-ZA-D1737	NA	NA	None	MCP -
Ecklonia radiata	S	89, C. Boothroyd	EradHL-ZA-D1769	NA	NA	Unknown	MCP +, MG967363
Ecklonia radiata	S	89, C. Boothroyd	EradHL-ZA-D1770	NA	NA	Unknown	MCP +, MG967364
Ecklonia radiata	S	89, C. Boothroyd	EradHL-ZA-D1771	NA	NA	Unknown	MCP +, MG967365
Ecklonia radiata	S	88, D. Kemp	EradKM-ZA-D1759	NA	NA	None	MCP -
Ecklonia radiata	S	88, D. Kemp	EradKM-ZA-D1760	NA	NA	Unknown	MCP +, MG967367
Ecklonia radiata	S	88, D. Kemp	EradKM-ZA-D1763	NA	NA	Unknown	MCP +, MG967366
Ecklonia radiata	S	86, D. Kemp	EradNV-ZA-D1762	NA	NA	None	MCP -
Ecklonia stolonifera	S	99, S. Uwai	EstoOM-JP-D1749	NA	NA	None	MCP -
Laminaria ochroleuca	S	72, J. Franco	LochAR-PT-3	NA	NA	None	MCP -

Laminaria pallida	S	79, C. Boothroyd	LpallDB-ZA-16	NA	NA	None	MCP -
Laminaria pallida	S	79, C. Boothroyd	LpallDB-ZA-17	NA	NA	None	MCP -
Laminaria pallida	S	79, C. Boothroyd	LpallDB-ZA-18	NA	NA	None	MCP -
Laminaria pallida	S	79, C. Boothroyd	LpallDB-ZA-19	NA	NA	None	MCP -
Laminaria pallida	S	79, C. Boothroyd	LpallDB-ZA-20	NA	NA	None	MCP -
Laminaria pallida	S	78, R. Anderson	LpallHB-ZA-14	NA	NA	None	MCP -
Laminaria pallida	S	80, R. Anderson	LpallJB-ZA-37	NA	NA	None	MCP -
Laminaria pallida	S	82, C. Boothroyd	LpallOK-ZA-1	NA	NA	None	MCP -
Laminaria pallida	S	82, C. Boothroyd	LpallOK-ZA-2	NA	NA	None	MCP -
Laminaria pallida	S	82, C. Boothroyd	LpallOK-ZA-4	NA	NA	None	MCP -
Laminaria pallida	S	77, M. Rothman	LpallPN-ZA-22	NA	NA	None	MCP -
Laminaria pallida	S	77, M. Rothman	LpallPN-ZA-24	NA	NA	None	MCP -
Laminaria pallida	S	83, S. John	LpallSC-ZA-3	NA	NA	None	MCP -
Laminaria pallida	S	83, S. John	LpallSC-ZA-4	NA	NA	None	MCP -
Laminaria pallida	S	75, B. Rothman	LpallSW-NA-2	NA	NA	None	MCP -
Laminaria pallida	S	75, B. Rothman	LpallSW-NA-3	NA	NA	None	MCP -
Lessonia spicata	S	20, C. Avaria	LspicPN-CL-1	2017	NA	None	MCP -
Lessonia spicata	S	20, C. Avaria	LspicPN-CL-2	2017	NA	None	MCP -
Lessonia spicata	S	20, C. Avaria	LspicPN-CL-3	2017	NA	None	MCP -
Lessonia spicata	S	20, C. Avaria	LspicPN-CL-4	2017	NA	None	MCP -
Lessonia spicata	S	20, C. Avaria	LspicPN-CL-5	2017	NA	None	MCP -
Macrocystis pyrifera	S	20, C. Avaria	MpyrPN-CL-1	2017	NA	None	MCP -
Macrocystis pyrifera	S	20, C. Avaria	MpyrPN-CL-2	2017	NA	None	MCP -
Macrocystis pyrifera	S	20, C. Avaria	MpyrPN-CL-3	2017	NA	C	MCP +, MG967376
Macrocystis pyrifera	S	20, C. Avaria	MpyrPN-CL-4	2017	NA	None	MCP -
Macrocystis pyrifera	S	20, C. Avaria	MpyrPN-CL-5	2017	NA	None	MCP -
Saccharina japonica	S	91, J. Park	SjapIC-SK-1	2017	NA	None	MCP -
Saccharina japonica	S	91, J. Park	SjapIC-SK-2	2017	NA	None	MCP -
Saccharina japonica	S	91, J. Park	SjapIC-SK-3	2017	NA	None	MCP -
Saccharina japonica	S	91, J. Park	SjapIC-SK-4	2017	NA	None	MCP -
Saccharina japonica	S	91, J. Park	SjapIC-SK-5	2017	NA	None	MCP -
Undaria pinnatifida	S	91, J. Park	UpinIC-SK-1	2017	NA	Unknown	MCP +, MG967371
Undaria pinnatifida	S	91, J. Park	UpinIC-SK-2	2017	NA	Unknown	MCP +, MG967372
Undaria pinnatifida	S	91, J. Park	UpinIC-SK-3	2017	NA	Unknown	MCP +, MG967373
Undaria pinnatifida	S	91, J. Park	UpinIC-SK-4	2017	NA	Unknown	MCP +, MG967374
Undaria pinnatifida	S	91, J. Park	UpinIC-SK-5	2017	NA	Unknown	MCP +, MG967375
Laminaria digitata	S	46, McKeown et al	LdigPM13	2015	NA	None	MCP -
Laminaria digitata	S	46, McKeown et al	LdigPM1	2015	NA	C	MCP +, KY063727
Laminaria digitata	S	46, McKeown et al	LdigPM2	2015	NA	C	MCP +, KY063726
Laminaria digitata	S	46, McKeown et al	LdigPM3	2015	NA	C	MCP +, KY063725
Laminaria digitata	S	46, McKeown et al	LdigPM4	2015	NA	C	MCP +, KY063724
Saccharina latissima	S	46, McKeown et al	SlatPM1	2015	NA	C	MCP +, KY063711
Saccharina latissima	S	46, McKeown et al	SlatPM7	2015	NA	None	MCP -
Saccharina latissima	S	46, McKeown et al	SlatPM2	2015	NA	C	MCP +, KY063710
Saccharina latissima	S	46, McKeown et al	SlatPM8	2015	NA	None	MCP -
Saccharina latissima	S	46, McKeown et al	SlatPM9	2015	NA	None	MCP -
Laminaria digitata	S	46, McKeown et al	LdigPM5	2015	NA	C	MCP +, KY063723
Laminaria digitata	S	46, McKeown et al	LdigPM6	2015	NA	C	MCP +, KY063722
Laminaria digitata	S	46, McKeown et al	LdigPM14	2015	NA	None	MCP -
Laminaria digitata	S	46, McKeown et al	LdigPM7	2015	NA	C	MCP + , KY063721
Laminaria digitata	S	46, McKeown et al	LdigPM15	2015	NA	None	MCP -
Saccharina latissima	S	46, McKeown et al	SlatPM3	2015	NA	C	MCP +, KY063709
Saccharina latissima	S	46, McKeown et al	SlatPM4	2015	NA	C	MCP +, KY063708
Saccharina latissima	S	46, McKeown et al	SlatPM10	2015	NA	None	MCP -
Saccharina latissima	S	46, McKeown et al	SlatPM11	2015	NA	None	MCP -
Saccharina latissima	S	46, McKeown et al	SlatPM12	2015	NA	None	MCP -
Laminaria digitata	S	46, McKeown et al	LdigPM8	2015	NA	C	MCP +, KY063720
Laminaria digitata	S	46, McKeown et al	LdigPM9	2015	NA	C	MCP +, KY063719
Laminaria digitata	S	46, McKeown et al	LdigPM10	2015	NA	C	MCP +, KY063718
Laminaria digitata	S	46, McKeown et al	LdigPM11	2015	NA	C	MCP +, KY063717
Laminaria digitata	S	46, McKeown et al	LdigPM12	2015	NA	C	MCP +, KY063716
Laminaria hyperborea	S	46, McKeown et al	LhypPM1	2015	NA	C	MCP +, KY063715
Laminaria hyperborea	S	46, McKeown et al	LhypPM2	2015	NA	C	MCP +, KY063714
Laminaria hyperborea	S	46, McKeown et al	LhypPM3	2015	NA	C	MCP +, KY063713
Laminaria hyperborea	S	46, McKeown et al	LhypPM4	2015	NA	C	MCP +, KY063712
Laminaria ochroleuca	S	46, D. McKeown	LochPM1	2015	NA	None	MCP -
Laminaria ochroleuca	S	46, D. McKeown	LochPM2	2015	NA	None	MCP -
Laminaria ochroleuca	S	46, D. McKeown	LochPM3	2015	NA	None	MCP -
Laminaria ochroleuca	S	46, D. McKeown	LochPM4	2015	NA	None	MCP -
Laminaria ochroleuca	S	46, D. McKeown	LochPM5	2015	NA	None	MCP -
Laminaria ochroleuca	S	46, D. McKeown	LochPM6	2015	NA	None	MCP -
Laminaria ochroleuca	S	46, D. McKeown	LochPM7	2015	NA	None	MCP -
Laminaria ochroleuca	S	46, D. McKeown	LochPM8	2015	NA	None	MCP -
Laminaria ochroleuca	S	46, D. McKeown	LochPM9	2015	NA	None	MCP -
Laminaria ochroleuca	S	46, D. McKeown	LochPM10	2015	NA	None	MCP -
Laminaria ochroleuca	S	46, D. McKeown	LochPM11	2015	NA	None	MCP -
Laminaria ochroleuca	S	46, D. McKeown	LochPM12	2015	NA	None	MCP -
Laminaria ochroleuca	S	46, D. McKeown	LochPM13	2015	NA	None	MCP -

Laminaria ochroleuca	S	46, D. McKeown	LochPM14	2015	NA	None	MCP -
Laminaria ochroleuca	S	46, D. McKeown	LochPM15	2015	NA	None	MCP -
Saccharina latissima	S	46, McKeown et al	SlatPM5	2015	NA	C	MCP +, KY063707
Saccharina latissima	S	46, McKeown et al	SlatPM6	2015	NA	C	MCP +, KY063706
Saccharina latissima	S	46, McKeown et al	SlatPM13	2015	NA	None	MCP -
Saccharina latissima	S	46, McKeown et al	SlatPM14	2015	NA	None	MCP -
Saccharina latissima	S	46, McKeown et al	SlatPM15	2015	NA	None	MCP -
Ectocarpus crouaniorum	NA	37, AF. Peters	ARB7C	09.06.04	Exposed	A+B	MCP + qPCR
Ectocarpus crouaniorum	NA	42, AF. Peters	TRE08-15C	21.06.08	Exposed	A+B	MCP + qPCR
Ectocarpus crouaniorum	NA	42, AF. Peters	TRE08-16C	21.06.08	Exposed	A+B	MCP + qPCR
Ectocarpus crouaniorum	NA	47, AF. Peters	WEM08-10C	21.05.08	Exposed	A+B	MCP + qPCR
Ectocarpus crouaniorum	NA	40, AF. Peters	BUT08-11A	02.06.08	Exposed	A+B	MCP + qPCR
Ectocarpus crouaniorum	NA	37, AF. Peters	ARB4C	09.06.04	Exposed	A+B	MCP + qPCR
Ectocarpus crouaniorum	NA	32, AF. Peters	WIC08-25C	12.06.08	Exposed	A+B	MCP + qPCR
Ectocarpus crouaniorum	NA	31, AF. Peters	SKY08-9	14.06.08	Exposed	A*	MCP + qPCR
Ectocarpus crouaniorum	NA	42, AF. Peters	TRE08-17A	21.06.08	Exposed	A+B	MCP + qPCR
Ectocarpus crouaniorum	NA	42, AF. Peters	TRE08-4B	21.06.08	Exposed	A+B	MCP + qPCR
Ectocarpus crouaniorum	NA	42, AF. Peters	TRE08-9B	21.06.08	Exposed	A+B	MCP + qPCR
Ectocarpus crouaniorum	NA	43, AF. Peters	RHOS08-1C	20.06.08	Exposed	A+B	MCP + qPCR
Ectocarpus crouaniorum	NA	47, AF. Peters	WEM08-15A	21.05.08	Exposed	A+B	MCP + qPCR
Ectocarpus crouaniorum	NA	47, AF. Peters	WEM08-16A	21.05.08	Exposed	A+B	MCP + qPCR
Ectocarpus crouaniorum	NA	47, AF. Peters	WEM08-19A	21.05.08	Exposed	A+B	MCP + qPCR
Ectocarpus crouaniorum	NA	47, AF. Peters	WEM08-20B	21.05.08	Exposed	A*	MCP + qPCR
Ectocarpus crouaniorum	NA	47, AF. Peters	WEM08-21C	21.05.08	Exposed	A+B	MCP + qPCR
Ectocarpus crouaniorum	NA	47, AF. Peters	WEM08-7A	21.05.08	Exposed	A*	MCP + qPCR
Ectocarpus crouaniorum	NA	51, AF. Peters	TOR4A	29.06.08	Exposed	A+B	MCP + qPCR
Ectocarpus crouaniorum	NA	52, AF. Peters	GOS4c	28.05.08	Exposed	A+B	MCP + qPCR
Ectocarpus crouaniorum	NA	40, AF. Peters	BUT08-13C	02.06.08	Exposed	A	MCP + qPCR
Ectocarpus crouaniorum	NA	40, AF. Peters	BUT08-1A	01.06.08	Exposed	A	MCP + qPCR
Ectocarpus crouaniorum	NA	40, AF. Peters	BUT08-4A	01.06.08	Exposed	A	MCP + qPCR
Ectocarpus crouaniorum	NA	40, AF. Peters	BUT08-5C	01.06.08	Exposed	A	MCP + qPCR
Ectocarpus crouaniorum	NA	40, AF. Peters	BUT28A	02.06.08	Exposed	A	MCP + qPCR
Ectocarpus crouaniorum	NA	37, AF. Peters	ARB1C	09.06.04	Exposed	A	MCP + qPCR
Ectocarpus crouaniorum	NA	37, AF. Peters	ARB8A3	09.06.04	Exposed	A	MCP + qPCR
Ectocarpus crouaniorum	NA	34, AF. Peters	RAT08-5C	10.06.08	Exposed	Unknown	MCP + qPCR
Ectocarpus crouaniorum	NA	34, AF. Peters	RAT9B	11.06.08	Exposed	A	MCP + qPCR
Ectocarpus crouaniorum	NA	32, AF. Peters	WIC08-10C	12.06.08	Exposed	A	MCP + qPCR
Ectocarpus crouaniorum	NA	32, AF. Peters	WIC08-16C	12.06.08	Exposed	Unknown	MCP + qPCR
Ectocarpus crouaniorum	NA	32, AF. Peters	WIC08-17A	12.06.08	Exposed	A	MCP + qPCR
Ectocarpus crouaniorum	NA	32, AF. Peters	WIC08-18A	12.06.08	Exposed	A	MCP + qPCR
Ectocarpus crouaniorum	NA	32, AF. Peters	WIC08-19C	12.06.08	Exposed	A	MCP + qPCR
Ectocarpus crouaniorum	NA	32, AF. Peters	WIC08-21B	12.06.08	Exposed	A	MCP + qPCR

Ectocarpus crouaniorum	NA	32, AF. Peters	WIC08-22C	12.06.08	Exposed	A	MCP + qPCR
Ectocarpus crouaniorum	NA	32, AF. Peters	WIC08-23A	12.06.08	Exposed	A	MCP + qPCR
Ectocarpus crouaniorum	NA	32, AF. Peters	WIC08-24B	12.06.08	Exposed	A	MCP + qPCR
Ectocarpus crouaniorum	NA	31, AF. Peters	SKY08-10C	14.06.08	Exposed	A	MCP + qPCR
Ectocarpus crouaniorum	NA	31, AF. Peters	SKY08-11C	14.06.08	Exposed	A	MCP + qPCR
Ectocarpus crouaniorum	NA	31, AF. Peters	SKY08-1C	14.06.08	Exposed	A	MCP + qPCR
Ectocarpus crouaniorum	NA	31, AF. Peters	SKY08-4C	14.06.08	Exposed	A	MCP + qPCR
Ectocarpus crouaniorum	NA	31, AF. Peters	SKY08-5C	14.06.08	Exposed	A	MCP + qPCR
Ectocarpus crouaniorum	NA	31, AF. Peters	SKY08-8C	14.06.08	Exposed	A	MCP + qPCR
Ectocarpus crouaniorum	NA	31, AF. Peters	SKY12B	14.06.08	Exposed	A	MCP + qPCR
Ectocarpus crouaniorum	NA	33, AF. Peters	LIA08-3B	15.06.08	Exposed	A	MCP + qPCR
Ectocarpus crouaniorum	NA	33, AF. Peters	LIA2A	15.06.08	Exposed	A	MCP + qPCR
Ectocarpus crouaniorum	NA	41, AF. Peters	GAL08-13A	17.06.08	Exposed	A	MCP + qPCR
Ectocarpus crouaniorum	NA	41, AF. Peters	GAL08-14C	17.06.08	Exposed	A	MCP + qPCR
Ectocarpus crouaniorum	NA	41, AF. Peters	GAL08-16B	17.06.08	Exposed	A	MCP + qPCR
Ectocarpus crouaniorum	NA	41, AF. Peters	GAL08-17B	17.06.08	Exposed	A	MCP + qPCR
Ectocarpus crouaniorum	NA	41, AF. Peters	GAL08-1A	17.06.08	Exposed	A	MCP + qPCR
Ectocarpus crouaniorum	NA	41, AF. Peters	GAL08-21C	17.06.08	Exposed	A	MCP + qPCR
Ectocarpus crouaniorum	NA	41, AF. Peters	GAL08-23C	17.06.08	Exposed	A	MCP + qPCR
Ectocarpus crouaniorum	NA	41, AF. Peters	GAL08-24B	17.06.08	Exposed	A	MCP + qPCR
Ectocarpus crouaniorum	NA	41, AF. Peters	GAL08-26C	17.06.08	Exposed	A	MCP + qPCR
Ectocarpus crouaniorum	NA	41, AF. Peters	GAL08-27C	17.06.08	Exposed	A	MCP + qPCR
Ectocarpus crouaniorum	NA	41, AF. Peters	GAL08-29C	17.06.08	Exposed	A	MCP + qPCR
Ectocarpus crouaniorum	NA	41, AF. Peters	GAL08-2C	17.06.08	Exposed	A	MCP + qPCR
Ectocarpus crouaniorum	NA	41, AF. Peters	GAL08-3C	17.06.08	Exposed	A	MCP + qPCR
Ectocarpus crouaniorum	NA	41, AF. Peters	GAL08-5C	17.06.08	Exposed	A	MCP + qPCR
Ectocarpus crouaniorum	NA	41, AF. Peters	GAL08-6C	17.06.08	Exposed	A	MCP + qPCR
Ectocarpus crouaniorum	NA	41, AF. Peters	GAL08-7C	17.06.08	Exposed	A	MCP + qPCR
Ectocarpus crouaniorum	NA	41, AF. Peters	GAL08-8C	17.06.08	Exposed	A	MCP + qPCR
Ectocarpus crouaniorum	NA	41, AF. Peters	GAL08-9C	17.06.08	Exposed	A	MCP + qPCR
Ectocarpus crouaniorum	NA	41, AF. Peters	GAL15A	17.06.08	Exposed	Unknown	MCP + qPCR
Ectocarpus crouaniorum	NA	41, AF. Peters	GAL22	17.06.08	Exposed	A	MCP + qPCR
Ectocarpus crouaniorum	NA	41, AF. Peters	GAL28A	17.06.08	Exposed	A	MCP + qPCR
Ectocarpus crouaniorum	NA	42, AF. Peters	TRE08-10B	21.06.08	Exposed	A	MCP + qPCR
Ectocarpus crouaniorum	NA	42, AF. Peters	TRE08-18C	21.06.08	Exposed	A	MCP + qPCR
Ectocarpus crouaniorum	NA	42, AF. Peters	TRE08-3C	21.06.08	Exposed	A	MCP + qPCR
Ectocarpus crouaniorum	NA	42, AF. Peters	TRE08-5A	21.06.08	Exposed	A	MCP + qPCR
Ectocarpus crouaniorum	NA	42, AF. Peters	TRE08-6C	21.06.08	Exposed	A	MCP + qPCR
Ectocarpus crouaniorum	NA	42, AF. Peters	TRE08-7A	21.06.08	Exposed	A	MCP + qPCR

Ectocarpus crouaniorum	NA	42, AF. Peters	TRE1B	21.06.08	Exposed	A	MCP + qPCR
Ectocarpus crouaniorum	NA	42, AF. Peters	TRE2C3	21.06.08	Exposed	A	MCP + qPCR
Ectocarpus crouaniorum	NA	42, AF. Peters	TRE8B4	21.06.08	Exposed	A	MCP + qPCR
Ectocarpus crouaniorum	NA	43, AF. Peters	RHOS08-2C	20.06.08	Exposed	A	MCP + qPCR
Ectocarpus crouaniorum	NA	43, AF. Peters	RHOS08-3C	20.06.08	Exposed	A	MCP + qPCR
Ectocarpus crouaniorum	NA	43, AF. Peters	RHOS08-4B	20.06.08	Exposed	A	MCP + qPCR
Ectocarpus crouaniorum	NA	47, AF. Peters	WEM08-11C	21.05.08	Exposed	A	MCP + qPCR
Ectocarpus crouaniorum	NA	47, AF. Peters	WEM08-12C	21.05.08	Exposed	A	MCP + qPCR
Ectocarpus crouaniorum	NA	47, AF. Peters	WEM08-13A	21.05.08	Exposed	A	MCP + qPCR
Ectocarpus crouaniorum	NA	47, AF. Peters	WEM08-14B	21.05.08	Exposed	A	MCP + qPCR
Ectocarpus crouaniorum	NA	47, AF. Peters	WEM08-17B	21.05.08	Exposed	A	MCP + qPCR
Ectocarpus crouaniorum	NA	47, AF. Peters	WEM08-18A	21.05.08	Exposed	A	MCP + qPCR
Ectocarpus crouaniorum	NA	47, AF. Peters	WEM08-22A	21.05.08	Exposed	A	MCP + qPCR
Ectocarpus crouaniorum	NA	47, AF. Peters	WEM08-23C	21.05.08	Exposed	A	MCP + qPCR
Ectocarpus crouaniorum	NA	47, AF. Peters	WEM08-24B	21.05.08	Exposed	A	MCP + qPCR
Ectocarpus crouaniorum	NA	47, AF. Peters	WEM08-25C	21.05.08	Exposed	A	MCP + qPCR
Ectocarpus crouaniorum	NA	47, AF. Peters	WEM08-26A	21.05.08	Exposed	A	MCP + qPCR
Ectocarpus crouaniorum	NA	47, AF. Peters	WEM08-27C	28.06.08	Exposed	B	MCP + qPCR
Ectocarpus crouaniorum	NA	47, AF. Peters	WEM08-6C	21.05.08	Exposed	A	MCP + qPCR
Ectocarpus crouaniorum	NA	52, AF. Peters	GOS1c	28.05.08	Exposed	A	MCP + qPCR
Ectocarpus crouaniorum	NA	32, AF. Peters	WIC08-11B	12.06.08	Exposed	None	MCP - qPCR
Ectocarpus crouaniorum	NA	32, AF. Peters	WIC08-12C	12.06.08	Exposed	None	MCP - qPCR
Ectocarpus crouaniorum	NA	32, AF. Peters	WIC08-13C	12.06.08	Exposed	None	MCP - qPCR
Ectocarpus crouaniorum	NA	32, AF. Peters	WIC08-15C	12.06.08	Exposed	None	MCP - qPCR
Ectocarpus crouaniorum	NA	42, AF. Peters	TRE08-19C	21.06.08	Exposed	None	MCP - qPCR
Ectocarpus crouaniorum	NA	43, AF. Peters	RHOS08-5C	20.06.08	Exposed	None	MCP - qPCR
Ectocarpus crouaniorum	NA	43, AF. Peters	RHOS08-6A	20.06.08	Exposed	None	MCP - qPCR
Ectocarpus crouaniorum	NA	35, AF. Peters	Mul(06-4)1	23.05.06	Unknown	A	MCP + qPCR
Ectocarpus crouaniorum	NA	50, AF. Peters	TOR6B2	29.06.08	Sheltered	A+B	MCP + qPCR
Ectocarpus crouaniorum	NA	56, AF. Peters	EcPH11-14	20.01.11	Sheltered	B*	MCP + qPCR
Ectocarpus crouaniorum	NA	56, AF. Peters	EcPH11-20	20.01.11	Sheltered	A+B	MCP + qPCR
Ectocarpus crouaniorum	NA	56, AF. Peters	EcPH11-25	20.01.11	Sheltered	B*	MCP + qPCR
Ectocarpus crouaniorum	NA	56, AF. Peters	EcPH11-43	20.01.11	Sheltered	B*	MCP + qPCR
Ectocarpus crouaniorum	NA	56, AF. Peters	EcPH11-5	20.01.11	Sheltered	B*	MCP + qPCR
Ectocarpus crouaniorum	NA	56, AF. Peters	EcPH11-s#2A-31	26.01.11	Sheltered	A+B	MCP + qPCR
Ectocarpus crouaniorum	NA	56, AF. Peters	EcPH11-s#2A-42	26.01.11	Sheltered	B*	MCP + qPCR
Ectocarpus crouaniorum	NA	56, AF. Peters	EcPH11-s#2A-44	26.01.11	Sheltered	A+B	MCP + qPCR
Ectocarpus crouaniorum	NA	62, AF. Peters	VF08-3C	19.04.08	Sheltered	A+B	MCP + qPCR
Ectocarpus crouaniorum	NA	62, AF. Peters	VF08-4A	19.04.08	Sheltered	A+B	MCP + qPCR

Ectocarpus crouaniorum	NA	50, AF. Peters	TOR08-5C	29.06.08	Sheltered	A+B	MCP + qPCR
Ectocarpus crouaniorum	NA	50, AF. Peters	TOR08-7B	29.06.08	Sheltered	A+B	MCP + qPCR
Ectocarpus crouaniorum	NA	56, AF. Peters	EcPH10-35	27.05.10	Sheltered	A+B	MCP + qPCR
Ectocarpus crouaniorum	NA	56, AF. Peters	EcPH11-113	27.06.11	Sheltered	A	MCP + qPCR
Ectocarpus crouaniorum	NA	56, AF. Peters	EcPH11-16	20.01.11	Sheltered	A+B	MCP + qPCR
Ectocarpus crouaniorum	NA	56, AF. Peters	EcPH11-18	20.01.11	Sheltered	A+B	MCP + qPCR
Ectocarpus crouaniorum	NA	56, AF. Peters	EcPH11-2	20.01.11	Sheltered	B	MCP + qPCR
Ectocarpus crouaniorum	NA	56, AF. Peters	EcPH11-26	20.01.11	Sheltered	A+B	MCP + qPCR
Ectocarpus crouaniorum	NA	56, AF. Peters	EcPH11-27	20.01.11	Sheltered	A+B	MCP + qPCR
Ectocarpus crouaniorum	NA	56, AF. Peters	EcPH11-3	20.01.11	Sheltered	A+B	MCP + qPCR
Ectocarpus crouaniorum	NA	56, AF. Peters	EcPH11-31	20.01.11	Sheltered	B*	MCP + qPCR
Ectocarpus crouaniorum	NA	56, AF. Peters	EcPH11-46	20.01.11	Sheltered	A+B	MCP + qPCR
Ectocarpus crouaniorum	NA	56, AF. Peters	EcPH11-6	28.04.11	Sheltered	A+B	MCP + qPCR
Ectocarpus crouaniorum	NA	56, AF. Peters	EcPH11-s#2A-19	26.01.11	Sheltered	A+B	MCP + qPCR
Ectocarpus crouaniorum	NA	56, AF. Peters	EcPH11-s#2B-20	25.01.11	Sheltered	A*	MCP + qPCR
Ectocarpus crouaniorum	NA	56, AF. Peters	EcPH11-s#2B-22	25.01.11	Sheltered	A+B	MCP + qPCR
Ectocarpus crouaniorum	NA	56, AF. Peters	EcPH11-s#2B-30	25.01.11	Sheltered	A+B	MCP + qPCR
Ectocarpus crouaniorum	NA	56, AF. Peters	EcPH11-s#2B-47	25.01.11	Sheltered	A+B	MCP + qPCR
Ectocarpus crouaniorum	NA	56, AF. Peters	EcPH11-s#2B-7	25.01.11	Sheltered	A+B	MCP + qPCR
Ectocarpus crouaniorum	NA	56, AF. Peters	EcPH11-s#5-38	21.01.11	Sheltered	A+B	MCP + qPCR
Ectocarpus crouaniorum	NA	56, AF. Peters	EcPH11-s#5-7	21.01.11	Sheltered	A*	MCP + qPCR
Ectocarpus crouaniorum	NA	58, AF. Peters	Ec244	13.07.11	Sheltered	A+B	MCP + qPCR
Ectocarpus crouaniorum	NA	62, AF. Peters	VF08-5A	19.04.08	Sheltered	A+B	MCP + qPCR
Ectocarpus crouaniorum	NA	50, AF. Peters	TOR08-8B	29.06.08	Sheltered	A	MCP + qPCR
Ectocarpus crouaniorum	NA	53, AF. Peters	LH6b	28.05.08	Sheltered	A	MCP + qPCR
Ectocarpus crouaniorum	NA	53, AF. Peters	LH8A	28.05.08	Sheltered	Unknown	MCP + qPCR
Ectocarpus crouaniorum	NA	54, AF. Peters	HAS08-10B	30.05.08	Sheltered	A	MCP + qPCR
Ectocarpus crouaniorum	NA	54, AF. Peters	HAS08-1C	30.05.08	Sheltered	A	MCP + qPCR
Ectocarpus crouaniorum	NA	56, AF. Peters	Ec195	13.01.10	Sheltered	A	MCP + qPCR
Ectocarpus crouaniorum	NA	56, AF. Peters	EcPH10-14	14.05.10	Sheltered	A	MCP + qPCR
Ectocarpus crouaniorum	NA	56, AF. Peters	EcPH10-24	27.05.10	Sheltered	Unknown	MCP + qPCR
Ectocarpus crouaniorum	NA	56, AF. Peters	EcPH10-28	27.05.10	Sheltered	Unknown	MCP + qPCR
Ectocarpus crouaniorum	NA	56, AF. Peters	EcPH10-29	27.05.10	Sheltered	A	MCP + qPCR
Ectocarpus crouaniorum	NA	56, AF. Peters	EcPH10-31	27.05.10	Sheltered	A	MCP + qPCR
Ectocarpus crouaniorum	NA	56, AF. Peters	EcPH10-33	27.05.10	Sheltered	A	MCP + qPCR
Ectocarpus crouaniorum	NA	56, AF. Peters	EcPH10-37	27.05.10	Sheltered	A	MCP + qPCR
Ectocarpus crouaniorum	NA	56, AF. Peters	EcPH10-60	27.05.10	Sheltered	Unknown	MCP + qPCR
Ectocarpus crouaniorum	NA	56, AF. Peters	EcPH10-63	27.05.10	Sheltered	A	MCP + qPCR
Ectocarpus crouaniorum	NA	56, AF. Peters	EcPH11-1	20.01.11	Sheltered	A	MCP + qPCR

Ectocarpus crouaniorum	NA	56, AF. Peters	EcPH11-106	06.06.11	Sheltered	A	MCP + qPCR
Ectocarpus crouaniorum	NA	56, AF. Peters	EcPH11-11	20.01.11	Sheltered	A	MCP + qPCR
Ectocarpus crouaniorum	NA	56, AF. Peters	EcPH11-112	27.06.11	Sheltered	A	MCP + qPCR
Ectocarpus crouaniorum	NA	56, AF. Peters	EcPH11-114	27.06.11	Sheltered	A	MCP + qPCR
Ectocarpus crouaniorum	NA	56, AF. Peters	EcPH11-13	20.01.11	Sheltered	Unknown	MCP + qPCR
Ectocarpus crouaniorum	NA	56, AF. Peters	EcPH11-17	20.01.11	Sheltered	A	MCP + qPCR
Ectocarpus crouaniorum	NA	56, AF. Peters	EcPH11-21	20.01.11	Sheltered	A	MCP + qPCR
Ectocarpus crouaniorum	NA	56, AF. Peters	EcPH11-28	20.01.11	Sheltered	A	MCP + qPCR
Ectocarpus crouaniorum	NA	56, AF. Peters	EcPH11-37	20.01.11	Sheltered	Unknown	MCP + qPCR
Ectocarpus crouaniorum	NA	56, AF. Peters	EcPH11-38	20.01.11	Sheltered	B	MCP + qPCR
Ectocarpus crouaniorum	NA	56, AF. Peters	EcPH11-39	20.01.11	Sheltered	A	MCP + qPCR
Ectocarpus crouaniorum	NA	56, AF. Peters	EcPH11-42	20.01.11	Sheltered	A	MCP + qPCR
Ectocarpus crouaniorum	NA	56, AF. Peters	EcPH11-47	20.01.11	Sheltered	A	MCP + qPCR
Ectocarpus crouaniorum	NA	56, AF. Peters	EcPH11-7	20.01.11	Sheltered	A	MCP + qPCR
Ectocarpus crouaniorum	NA	56, AF. Peters	EcPH11-9	20.01.11	Sheltered	A	MCP + qPCR
Ectocarpus crouaniorum	NA	56, AF. Peters	EcPH11-s#2A-1	26.01.11	Sheltered	A	MCP + qPCR
Ectocarpus crouaniorum	NA	56, AF. Peters	EcPH11-s#2A-14	26.01.11	Sheltered	Unknown	MCP + qPCR
Ectocarpus crouaniorum	NA	56, AF. Peters	EcPH11-s#2A-29	26.01.11	Sheltered	A	MCP + qPCR
Ectocarpus crouaniorum	NA	56, AF. Peters	EcPH11-s#2A-46	26.01.11	Sheltered	A	MCP + qPCR
Ectocarpus crouaniorum	NA	56, AF. Peters	EcPH11-s#2B-1	25.01.11	Sheltered	A	MCP + qPCR
Ectocarpus crouaniorum	NA	56, AF. Peters	EcPH11-s#2B-10	25.01.11	Sheltered	A	MCP + qPCR
Ectocarpus crouaniorum	NA	56, AF. Peters	EcPH11-s#2B-17	25.01.11	Sheltered	Unknown	MCP + qPCR
Ectocarpus crouaniorum	NA	56, AF. Peters	EcPH11-s#2B-2	25.01.11	Sheltered	A	MCP + qPCR
Ectocarpus crouaniorum	NA	56, AF. Peters	EcPH11-s#2B-21	25.01.11	Sheltered	A	MCP + qPCR
Ectocarpus crouaniorum	NA	56, AF. Peters	EcPH11-s#2B-23	25.01.11	Sheltered	A	MCP + qPCR
Ectocarpus crouaniorum	NA	56, AF. Peters	EcPH11-s#2B-25	25.01.11	Sheltered	B	MCP + qPCR
Ectocarpus crouaniorum	NA	56, AF. Peters	EcPH11-s#2B-28	25.01.11	Sheltered	A	MCP + qPCR
Ectocarpus crouaniorum	NA	56, AF. Peters	EcPH11-s#2B-3	25.01.11	Sheltered	A	MCP + qPCR
Ectocarpus crouaniorum	NA	56, AF. Peters	EcPH11-s#2B-35	25.01.11	Sheltered	A	MCP + qPCR
Ectocarpus crouaniorum	NA	56, AF. Peters	EcPH11-s#2B-37	25.01.11	Sheltered	A	MCP + qPCR
Ectocarpus crouaniorum	NA	56, AF. Peters	EcPH11-s#2B-4	25.01.11	Sheltered	A	MCP + qPCR
Ectocarpus crouaniorum	NA	56, AF. Peters	EcPH11-s#2B-42	25.01.11	Sheltered	A	MCP + qPCR
Ectocarpus crouaniorum	NA	56, AF. Peters	EcPH11-s#2B-52	25.01.11	Sheltered	A	MCP + qPCR
Ectocarpus crouaniorum	NA	56, AF. Peters	EcPH11-s#2B-53	25.01.11	Sheltered	A	MCP + qPCR
Ectocarpus crouaniorum	NA	56, AF. Peters	EcPH11-s#2B-54	25.01.11	Sheltered	A	MCP + qPCR
Ectocarpus crouaniorum	NA	56, AF. Peters	EcPH11-s#2B-9	25.01.11	Sheltered	Unknown	MCP + qPCR
Ectocarpus crouaniorum	NA	55, AF. Peters	Ec326	27.05.06	Sheltered	B	MCP + qPCR
Ectocarpus crouaniorum	NA	55, AF. Peters	Ec329	27.05.06	Sheltered	A	MCP + qPCR
Ectocarpus crouaniorum	NA	58, AF. Peters	Ec243	13.07.11	Sheltered	A	MCP + qPCR

Ectocarpus crouaniorum	NA	62, AF. Peters	VF08-2C	19.04.08	Sheltered	A	MCP + qPCR
Ectocarpus crouaniorum	NA	56, AF. Peters	Ec334hSP	14.04.07	Sheltered	None	MCP - qPCR
Ectocarpus crouaniorum	NA	56, AF. Peters	EcPH10-13	14.05.10	Sheltered	None	MCP - qPCR
Ectocarpus crouaniorum	NA	56, AF. Peters	EcPH10-22	27.05.10	Sheltered	None	MCP - qPCR
Ectocarpus crouaniorum	NA	56, AF. Peters	EcPH10-23	27.05.10	Sheltered	None	MCP - qPCR
Ectocarpus crouaniorum	NA	56, AF. Peters	EcPH10-25	27.05.10	Sheltered	None	MCP - qPCR
Ectocarpus crouaniorum	NA	56, AF. Peters	EcPH10-32	27.05.10	Sheltered	None	MCP - qPCR
Ectocarpus crouaniorum	NA	56, AF. Peters	EcPH10-34	27.05.10	Sheltered	None	MCP - qPCR
Ectocarpus crouaniorum	NA	56, AF. Peters	EcPH10-36	27.05.10	Sheltered	None	MCP - qPCR
Ectocarpus crouaniorum	NA	56, AF. Peters	EcPH10-4	01.03.10	Sheltered	None	MCP - qPCR
Ectocarpus crouaniorum	NA	56, AF. Peters	EcPH10-45	27.05.10	Sheltered	None	MCP - qPCR
Ectocarpus crouaniorum	NA	56, AF. Peters	EcPH10-46	27.05.10	Sheltered	None	MCP - qPCR
Ectocarpus crouaniorum	NA	56, AF. Peters	EcPH10-48	27.05.10	Sheltered	None	MCP - qPCR
Ectocarpus crouaniorum	NA	56, AF. Peters	EcPH10-49	27.05.10	Sheltered	None	MCP - qPCR
Ectocarpus crouaniorum	NA	56, AF. Peters	EcPH10-50	27.05.10	Sheltered	None	MCP - qPCR
Ectocarpus crouaniorum	NA	56, AF. Peters	EcPH10-51	27.05.10	Sheltered	None	MCP - qPCR
Ectocarpus crouaniorum	NA	56, AF. Peters	EcPH10-54	27.05.10	Sheltered	None	MCP - qPCR
Ectocarpus crouaniorum	NA	56, AF. Peters	EcPH10-55	27.05.10	Sheltered	None	MCP - qPCR
Ectocarpus crouaniorum	NA	56, AF. Peters	EcPH10-57	27.05.10	Sheltered	None	MCP - qPCR
Ectocarpus crouaniorum	NA	56, AF. Peters	EcPH10-58	27.05.10	Sheltered	None	MCP - qPCR
Ectocarpus crouaniorum	NA	56, AF. Peters	EcPH10-64	27.05.10	Sheltered	None	MCP - qPCR
Ectocarpus crouaniorum	NA	56, AF. Peters	EcPH10-65	27.05.10	Sheltered	None	MCP - qPCR
Ectocarpus crouaniorum	NA	56, AF. Peters	EcPH10-70	27.05.10	Sheltered	None	MCP - qPCR
Ectocarpus crouaniorum	NA	56, AF. Peters	EcPH10-8	28.04.10	Sheltered	None	MCP - qPCR
Ectocarpus crouaniorum	NA	56, AF. Peters	EcPH11-10	20.01.11	Sheltered	None	MCP - qPCR
Ectocarpus crouaniorum	NA	56, AF. Peters	EcPH11-12	20.01.11	Sheltered	None	MCP - qPCR
Ectocarpus crouaniorum	NA	56, AF. Peters	EcPH11-33	20.01.11	Sheltered	None	MCP - qPCR
Ectocarpus crouaniorum	NA	56, AF. Peters	EcPH11-35	20.01.11	Sheltered	None	MCP - qPCR
Ectocarpus crouaniorum	NA	56, AF. Peters	EcPH11-36	20.01.11	Sheltered	None	MCP - qPCR
Ectocarpus crouaniorum	NA	56, AF. Peters	EcPH11-40	20.01.11	Sheltered	None	MCP - qPCR
Ectocarpus crouaniorum	NA	56, AF. Peters	EcPH11-s#2A-18	26.01.11	Sheltered	None	MCP - qPCR
Ectocarpus crouaniorum	NA	56, AF. Peters	EcPH11-s#2A-21	26.01.11	Sheltered	None	MCP - qPCR
Ectocarpus crouaniorum	NA	56, AF. Peters	EcPH11-s#2A-24	26.01.11	Sheltered	None	MCP - qPCR
Ectocarpus crouaniorum	NA	56, AF. Peters	EcPH11-s#2A-36	26.01.11	Sheltered	None	MCP - qPCR
Ectocarpus crouaniorum	NA	56, AF. Peters	EcPH11-s#2B-16	25.01.11	Sheltered	None	MCP - qPCR
Ectocarpus crouaniorum	NA	56, AF. Peters	EcPH11-s#2B-2	25.01.11	Sheltered	A	MCP + qPCR
Ectocarpus crouaniorum	NA	56, AF. Peters	EcPH11-s#2B-26	25.01.11	Sheltered	None	MCP - qPCR
Ectocarpus crouaniorum	NA	56, AF. Peters	EcPH11-s#2B-32	25.01.11	Sheltered	None	MCP - qPCR
Ectocarpus crouaniorum	NA	56, AF. Peters	EcPH11-s#2B-34	25.01.11	Sheltered	None	MCP - qPCR

Ectocarpus crouaniorum	NA	56, AF. Peters	EcPH11-s#2B-36	25.01.11	Sheltered	None	MCP - qPCR
Ectocarpus crouaniorum	NA	56, AF. Peters	EcPH11-s#2B-39	25.01.11	Sheltered	None	MCP - qPCR
Ectocarpus crouaniorum	NA	56, AF. Peters	EcPH11-s#2B-49	25.01.11	Sheltered	None	MCP - qPCR
Ectocarpus crouaniorum	NA	56, AF. Peters	EcPH11-s#5-44	21.01.11	Sheltered	None	MCP - qPCR
Ectocarpus crouaniorum	NA	56, AF. Peters	EcPH11-s#5-47	21.01.11	Sheltered	None	MCP - qPCR
Ectocarpus fasciculatus	NA	13, AF. Peters	Ec308	06.03.06	Exposed	B*	MCP + qPCR
Ectocarpus fasciculatus	NA	43, AF. Peters	RHOS08-15A	20.06.08	Exposed	A+B	MCP + qPCR
Ectocarpus fasciculatus	NA	14, AF. Peters	Ec310	01.03.09	Exposed	B*	MCP + qPCR
Ectocarpus fasciculatus	NA	40, AF. Peters	BUT08-14C	02.06.08	Exposed	B	MCP + qPCR
Ectocarpus fasciculatus	NA	40, AF. Peters	BUT08-16A	02.06.08	Exposed	A+B	MCP + qPCR
Ectocarpus fasciculatus	NA	43, AF. Peters	RHOS08-16A	20.06.08	Exposed	B*	MCP + qPCR
Ectocarpus fasciculatus	NA	47, AF. Peters	WEM08-2A	21.05.08	Exposed	A+B	MCP + qPCR
Ectocarpus fasciculatus	NA	52, AF. Peters	GOS3b	28.05.08	Exposed	B	MCP + qPCR
Ectocarpus fasciculatus	NA	56, AF. Peters	(Ec)PHH9	13.08.06	Exposed	A+B	MCP + qPCR
Ectocarpus fasciculatus	NA	56, AF. Peters	EcPH10-255	11.08.10	Exposed	B*	MCP + qPCR
Ectocarpus fasciculatus	NA	56, AF. Peters	EcPH10-273	12.08.10	Exposed	A+B	MCP + qPCR
Ectocarpus fasciculatus	NA	56, AF. Peters	EcPH10-274	12.08.10	Exposed	A+B	MCP + qPCR
Ectocarpus fasciculatus	NA	56, AF. Peters	EcPH10-279	12.08.10	Exposed	A+B	MCP + qPCR
Ectocarpus fasciculatus	NA	56, AF. Peters	EcPH10-285	12.08.10	Exposed	A+B	MCP + qPCR
Ectocarpus fasciculatus	NA	19, A. Mann	QY16	05.10.06	Exposed	Unknown	MCP + qPCR
Ectocarpus fasciculatus	NA	19, A. Mann	QY19	05.10.06	Exposed	A	MCP + qPCR
Ectocarpus fasciculatus	NA	19, A. Mann	QY21	05.10.06	Exposed	Unknown	MCP + qPCR
Ectocarpus fasciculatus	NA	19, A. Mann	QY22	05.10.06	Exposed	Unknown	MCP + qPCR
Ectocarpus fasciculatus	NA	19, A. Mann	QY23	05.10.06	Exposed	A	MCP + qPCR
Ectocarpus fasciculatus	NA	40, AF. Peters	BUT08-15B	02.06.08	Exposed	B	MCP + qPCR
Ectocarpus fasciculatus	NA	40, AF. Peters	BUT08-17B	02.06.08	Exposed	B	MCP + qPCR
Ectocarpus fasciculatus	NA	40, AF. Peters	BUT08-2B	01.06.08	Exposed	A	MCP + qPCR
Ectocarpus fasciculatus	NA	40, AF. Peters	BUT08-3C	01.06.08	Exposed	A	MCP + qPCR
Ectocarpus fasciculatus	NA	40, AF. Peters	BUT20A	02.06.08	Exposed	B	MCP + qPCR
Ectocarpus fasciculatus	NA	32, AF. Peters	WIC08-26C	12.06.08	Exposed	A	MCP + qPCR
Ectocarpus fasciculatus	NA	32, AF. Peters	WIC08-28A	12.06.08	Exposed	B	MCP + qPCR
Ectocarpus fasciculatus	NA	32, AF. Peters	WIC08-29C	12.06.08	Exposed	B	MCP + qPCR
Ectocarpus fasciculatus	NA	32, AF. Peters	WIC08-30C	12.06.08	Exposed	A	MCP + qPCR
Ectocarpus fasciculatus	NA	41, AF. Peters	GAL08-10C	17.06.08	Exposed	Unknown	MCP + qPCR
Ectocarpus fasciculatus	NA	41, AF. Peters	GAL08-12A	17.06.08	Exposed	B	MCP + qPCR
Ectocarpus fasciculatus	NA	41, AF. Peters	GAL08-18A	17.06.08	Exposed	B	MCP + qPCR
Ectocarpus fasciculatus	NA	41, AF. Peters	GAL08-19C	17.06.08	Exposed	B	MCP + qPCR
Ectocarpus fasciculatus	NA	41, AF. Peters	GAL08-4C	17.06.08	Exposed	B	MCP + qPCR
Ectocarpus fasciculatus	NA	42, AF. Peters	TRE08-14B	21.06.08	Exposed	B	MCP + qPCR
Ectocarpus fasciculatus	NA	43, AF. Peters	RHO14C	20.06.08	Exposed	Unknown	MCP + qPCR
Ectocarpus fasciculatus	NA	47, DC. Schroeder	W004	05.07.04	Exposed	A	MCP + qPCR
Ectocarpus fasciculatus	NA	47, AF. Peters	WEM08-1A	21.05.08	Exposed	A	MCP + qPCR
Ectocarpus fasciculatus	NA	47, AF. Peters	WEM4B	21.05.08	Exposed	A	MCP + qPCR
Ectocarpus fasciculatus	NA	56, AF. Peters	(Ec)PHL10	13.08.06	Exposed	Unknown	MCP + qPCR
Ectocarpus fasciculatus	NA	56, AF. Peters	(Ec)PHL12	08.09.06	Exposed	Unknown	MCP + qPCR
Ectocarpus fasciculatus	NA	56, AF. Peters	(Ec)PHL30	08.09.06	Exposed	Unknown	MCP + qPCR
Ectocarpus fasciculatus	NA	56, AF. Peters	(Ec)PHL6	13.08.06	Exposed	B	MCP + qPCR
Ectocarpus fasciculatus	NA	56, AF. Peters	Ec680	13.08.06	Exposed	B	MCP + qPCR
Ectocarpus fasciculatus	NA	56, AF. Peters	Ec684	08.09.06	Exposed	Unknown	MCP + qPCR
Ectocarpus fasciculatus	NA	56, AF. Peters	EcPH10-245	11.08.10	Exposed	Unknown	MCP + qPCR
Ectocarpus fasciculatus	NA	56, AF. Peters	EcPH10-264	11.08.10	Exposed	A	MCP + qPCR
Ectocarpus fasciculatus	NA	56, AF. Peters	EcPH10-267	11.08.10	Exposed	Unknown	MCP + qPCR
Ectocarpus fasciculatus	NA	56, AF. Peters	EcPH10-270	11.08.10	Exposed	A	MCP + qPCR
Ectocarpus fasciculatus	NA	56, AF. Peters	EcPH10-271	12.08.10	Exposed	Unknown	MCP + qPCR
Ectocarpus fasciculatus	NA	56, AF. Peters	EcPH10-275	12.08.10	Exposed	Unknown	MCP + qPCR
Ectocarpus fasciculatus	NA	56, AF. Peters	EcPH10-277	12.08.10	Exposed	B	MCP + qPCR
Ectocarpus fasciculatus	NA	56, AF. Peters	EcPH10-278	12.08.10	Exposed	B	MCP + qPCR
Ectocarpus fasciculatus	NA	56, AF. Peters	EcPH10-281	12.08.10	Exposed	B	MCP + qPCR
Ectocarpus fasciculatus	NA	56, AF. Peters	EcPH10-283	12.08.10	Exposed	A	MCP + qPCR
Ectocarpus fasciculatus	NA	56, AF. Peters	EcPH10-287	12.08.10	Exposed	Unknown	MCP + qPCR
Ectocarpus fasciculatus	NA	56, AF. Peters	EcPH10-288	12.08.10	Exposed	A	MCP + qPCR
Ectocarpus fasciculatus	NA	19, A. Mann	QY20	05.10.06	Exposed	None	MCP - qPCR
Ectocarpus fasciculatus	NA	40, AF. Peters	BUT7B	01.06.08	Exposed	None	MCP - qPCR
Ectocarpus fasciculatus	NA	34, AF. Peters	RAT1C4	10.06.08	Exposed	None	MCP - qPCR
Ectocarpus fasciculatus	NA	32, AF. Peters	3227A	12.06.08	Exposed	None	MCP - qPCR
Ectocarpus fasciculatus	NA	33, AF. Peters	LIA08-1C	15.06.08	Exposed	None	MCP - qPCR
Ectocarpus fasciculatus	NA	41, AF. Peters	GAL08-11C	17.06.08	Exposed	None	MCP - qPCR
Ectocarpus fasciculatus	NA	41, AF. Peters	GAL08-20C	17.06.08	Exposed	None	MCP - qPCR
Ectocarpus fasciculatus	NA	41, AF. Peters	GAL08-25A	17.06.08	Exposed	None	MCP - qPCR
Ectocarpus fasciculatus	NA	42, AF. Peters	TRE08-12C	21.06.08	Exposed	None	MCP - qPCR
Ectocarpus fasciculatus	NA	47, AF. Peters	WEM08-3A	21.05.08	Exposed	None	MCP - qPCR
Ectocarpus fasciculatus	NA	47, AF. Peters	WEM08-5B	21.05.08	Exposed	None	MCP - qPCR
Ectocarpus fasciculatus	NA	51, AF. Peters	TOR08-1C	29.06.08	Exposed	None	MCP - qPCR

Ectocarpus fasciculatus	NA	51, AF. Peters	TOR08-2A	29.06.08	Exposed	None	MCP - qPCR
Ectocarpus fasciculatus	NA	56, AF. Peters	(Ec)PHH2	13.08.06	Exposed	None	MCP - qPCR
Ectocarpus fasciculatus	NA	56, AF. Peters	(Ec)PHH7	13.08.06	Exposed	None	MCP - qPCR
Ectocarpus fasciculatus	NA	56, AF. Peters	(Ec)PHL1	12.08.06	Exposed	None	MCP - qPCR
Ectocarpus fasciculatus	NA	56, AF. Peters	(Ec)PHL2	13.08.06	Exposed	None	MCP - qPCR
Ectocarpus fasciculatus	NA	56, AF. Peters	(Ec)PHL26	08.09.06	Exposed	None	MCP - qPCR
Ectocarpus fasciculatus	NA	56, AF. Peters	Ec396	30.09.03	Exposed	None	MCP - qPCR
Ectocarpus fasciculatus	NA	56, AF. Peters	Ec578	29.09.05	Exposed	None	MCP - qPCR
Ectocarpus fasciculatus	NA	56, AF. Peters	Ec674	13.08.06	Exposed	None	MCP - qPCR
Ectocarpus fasciculatus	NA	56, AF. Peters	Ec683	08.09.06	Exposed	None	MCP - qPCR
Ectocarpus fasciculatus	NA	56, AF. Peters	EcPH10-227	11.08.07	Exposed	None	MCP - qPCR
Ectocarpus fasciculatus	NA	56, AF. Peters	EcPH10-227	11.08.07	Exposed	None	MCP - qPCR
Ectocarpus fasciculatus	NA	56, AF. Peters	EcPH10-228	11.08.07	Exposed	None	MCP - qPCR
Ectocarpus fasciculatus	NA	56, AF. Peters	EcPH10-228	11.08.07	Exposed	None	MCP - qPCR
Ectocarpus fasciculatus	NA	56, AF. Peters	EcPH10-234	11.08.10	Exposed	None	MCP - qPCR
Ectocarpus fasciculatus	NA	56, AF. Peters	EcPH10-234	11.08.10	Exposed	None	MCP - qPCR
Ectocarpus fasciculatus	NA	56, AF. Peters	EcPH10-240	11.08.10	Exposed	None	MCP - qPCR
Ectocarpus fasciculatus	NA	56, AF. Peters	EcPH10-240	11.08.10	Exposed	None	MCP - qPCR
Ectocarpus fasciculatus	NA	56, AF. Peters	EcPH10-245	11.08.10	Exposed	Unknown	MCP + qPCR
Ectocarpus fasciculatus	NA	56, AF. Peters	EcPH10-248	11.08.10	Exposed	None	MCP - qPCR
Ectocarpus fasciculatus	NA	56, AF. Peters	EcPH10-248	11.08.10	Exposed	None	MCP - qPCR
Ectocarpus fasciculatus	NA	56, AF. Peters	EcPH10-255	11.08.10	Exposed	B*	MCP + qPCR
Ectocarpus fasciculatus	NA	56, AF. Peters	EcPH10-260	11.08.10	Exposed	None	MCP - qPCR
Ectocarpus fasciculatus	NA	56, AF. Peters	EcPH10-260	11.08.10	Exposed	None	MCP - qPCR
Ectocarpus fasciculatus	NA	56, AF. Peters	EcPH10-262	11.08.10	Exposed	None	MCP - qPCR
Ectocarpus fasciculatus	NA	56, AF. Peters	EcPH10-262	11.08.10	Exposed	None	MCP - qPCR
Ectocarpus fasciculatus	NA	56, AF. Peters	EcPH10-264	11.08.10	Exposed	A	MCP + qPCR
Ectocarpus fasciculatus	NA	56, AF. Peters	EcPH10-267	11.08.10	Exposed	Unknown	MCP + qPCR
Ectocarpus fasciculatus	NA	56, AF. Peters	EcPH10-270	11.08.10	Exposed	A	MCP + qPCR
Ectocarpus fasciculatus	NA	56, AF. Peters	EcPH10-272	12.08.10	Exposed	None	MCP - qPCR
Ectocarpus fasciculatus	NA	56, AF. Peters	EcPH10-276	12.08.10	Exposed	None	MCP - qPCR
Ectocarpus fasciculatus	NA	56, AF. Peters	EcPH10-282	12.08.10	Exposed	None	MCP - qPCR
Ectocarpus fasciculatus	NA	56, AF. Peters	EcPH10-284	12.08.10	Exposed	None	MCP - qPCR
Ectocarpus fasciculatus	NA	56, AF. Peters	EcPH10-286	12.08.10	Exposed	None	MCP - qPCR
Ectocarpus fasciculatus	NA	56, AF. Peters	EcPH10-289	12.08.10	Exposed	None	MCP - qPCR
Ectocarpus fasciculatus	NA	56, AF. Peters	EcPH10-290	12.08.10	Exposed	None	MCP - qPCR
Ectocarpus fasciculatus	NA	56, AF. Peters	EcPH10-5	01.03.10	Exposed	None	MCP - qPCR
Ectocarpus fasciculatus	NA	17, D. Müller	CH(05-)11	10.01.04	NA	A+B	MCP + qPCR
Ectocarpus fasciculatus	NA	48, DC. Schroeder	M001	23.11.03	NA	A+B	MCP + qPCR
Ectocarpus fasciculatus	NA	49, DC. Schroeder	S001	04.06.04	NA	B*	MCP + qPCR
Ectocarpus fasciculatus	NA	49, DC. Schroeder	S002	04.06.04	NA	A+B	MCP + qPCR
Ectocarpus fasciculatus	NA	17, D. Müller	60(05-)12	10.01.04	NA	A	MCP + qPCR
Ectocarpus fasciculatus	NA	46, DC. Schroeder	P002	04.06.04	NA	A	MCP + qPCR
Ectocarpus fasciculatus	NA	46, DC. Schroeder	P005	24.02.04	NA	Unknown	MCP + qPCR
Ectocarpus fasciculatus	NA	46, DC. Schroeder	P006	23.07.04	NA	Unknown	MCP + qPCR
Ectocarpus fasciculatus	NA	45, DC. Schroeder	Y003	30.07.04	NA	None	MCP - qPCR
Ectocarpus fasciculatus	NA	46, DC. Schroeder	P001	04.06.04	NA	None	MCP - qPCR
Ectocarpus fasciculatus	NA	49, AF. Peters	SAL08-2C	29.06.08	Sheltered	B*	MCP + qPCR
Ectocarpus fasciculatus	NA	56, AF. Peters	EcPH10-178	15.07.10	Sheltered	B*	MCP + qPCR
Ectocarpus fasciculatus	NA	59,	EcQB10-2	31.07.10	Sheltered	A+B	MCP + qPCR
Ectocarpus fasciculatus	NA	36, AF. Peters	SAM08-8C	07.06.08	Sheltered	A+B	MCP + qPCR
Ectocarpus fasciculatus	NA	46, AF. Peters	QAB08-4B	24.05.08	Sheltered	A+B	MCP + qPCR
Ectocarpus fasciculatus	NA	49, AF. Peters	SAL08-1C	29.06.08	Sheltered	A+B	MCP + qPCR
Ectocarpus fasciculatus	NA	54, AF. Peters	HAS08-2B	30.05.08	Sheltered	A+B	MCP + qPCR
Ectocarpus fasciculatus	NA	56, AF. Peters	(Ec)PHZ5	24.08.06	Sheltered	A+B	MCP + qPCR
Ectocarpus fasciculatus	NA	59,	EcQB10-11	31.07.10	Sheltered	A+B	MCP + qPCR
Ectocarpus fasciculatus	NA	59,	EcQB10-15	31.07.10	Sheltered	A+B	MCP + qPCR
Ectocarpus fasciculatus	NA	59,	EcQB10-18	31.07.10	Sheltered	A+B	MCP + qPCR
Ectocarpus fasciculatus	NA	14, AF. Peters	Ec165	01.03.06	Sheltered	A	MCP + qPCR
Ectocarpus fasciculatus	NA	36, AF. Peters	Ob07-2	07.11.07	Sheltered	Unknown	MCP + qPCR
Ectocarpus fasciculatus	NA	50, AF. Peters	TOR08-15B	29.06.08	Sheltered	Unknown	MCP + qPCR
Ectocarpus fasciculatus	NA	54, AF. Peters	HAS08-4C	30.05.08	Sheltered	A	MCP + qPCR
Ectocarpus fasciculatus	NA	56, AF. Peters	(Ec)PHZ17	24.08.06	Sheltered	A	MCP + qPCR
Ectocarpus fasciculatus	NA	56, AF. Peters	(Ec)PHZ35	30.08.07	Sheltered	A	MCP + qPCR
Ectocarpus fasciculatus	NA	56, AF. Peters	(Ec)PHZ6	24.08.06	Sheltered	A	MCP + qPCR
Ectocarpus fasciculatus	NA	56, AF. Peters	(Ec)PHZ8	24.08.06	Sheltered	A	MCP + qPCR
Ectocarpus fasciculatus	NA	56, AF. Peters	EcPH10-186	15.07.10	Sheltered	B	MCP + qPCR
Ectocarpus fasciculatus	NA	59,	EcQB10-10	31.07.10	Sheltered	A	MCP + qPCR
Ectocarpus fasciculatus	NA	59,	EcQB10-12	31.07.10	Sheltered	A	MCP + qPCR
Ectocarpus fasciculatus	NA	59,	EcQB10-13	31.07.10	Sheltered	A	MCP + qPCR
Ectocarpus fasciculatus	NA	59,	EcQB10-5	31.07.10	Sheltered	A	MCP + qPCR
Ectocarpus fasciculatus	NA	59,	EcQB10-6	31.07.10	Sheltered	A	MCP + qPCR
Ectocarpus fasciculatus	NA	59,	EcQB10-9	31.07.10	Sheltered	A	MCP + qPCR
Ectocarpus fasciculatus	NA	36, AF. Peters	Ob07-12C	07.11.07	Sheltered	None	MCP - qPCR
Ectocarpus fasciculatus	NA	36, AF. Peters	Ob07-13C	07.11.07	Sheltered	None	MCP - qPCR
Ectocarpus fasciculatus	NA	36, AF. Peters	Ob07-7	07.11.07	Sheltered	None	MCP - qPCR
Ectocarpus fasciculatus	NA	36, AF. Peters	SAM08-1B	07.06.08	Sheltered	None	MCP - qPCR
Ectocarpus fasciculatus	NA	36, AF. Peters	SAM08-6C	07.06.08	Sheltered	None	MCP - qPCR

Ectocarpus fasciculatus	NA	36, AF. Peters	SAM2	07.06.08	Sheltered	None	MCP - qPCR
Ectocarpus fasciculatus	NA	36, AF. Peters	SAM7A	07.06.08	Sheltered	None	MCP - qPCR
Ectocarpus fasciculatus	NA	38, AF. Peters	SAM08-13C	08.06.08	Sheltered	None	MCP - qPCR
Ectocarpus fasciculatus	NA	39, AF. Peters	POR08-3C	17.06.08	Sheltered	None	MCP - qPCR
Ectocarpus fasciculatus	NA	39, AF. Peters	POR08-4C	17.06.08	Sheltered	None	MCP - qPCR
Ectocarpus fasciculatus	NA	43, AF. Peters	RHOS08-10C	20.06.08	Sheltered	None	MCP - qPCR
Ectocarpus fasciculatus	NA	43, AF. Peters	RHOS08-11B	20.06.08	Sheltered	None	MCP - qPCR
Ectocarpus fasciculatus	NA	43, AF. Peters	RHOS08-13B	20.06.08	Sheltered	None	MCP - qPCR
Ectocarpus fasciculatus	NA	43, AF. Peters	RHOS08-7C	20.06.08	Sheltered	None	MCP - qPCR
Ectocarpus fasciculatus	NA	43, AF. Peters	RHOS08-8B	20.06.08	Sheltered	None	MCP - qPCR
Ectocarpus fasciculatus	NA	43, AF. Peters	RHOS08-9B	20.06.08	Sheltered	None	MCP - qPCR
Ectocarpus fasciculatus	NA	44, AF. Peters	REP10-2	14.06.10	Sheltered	None	MCP - qPCR
Ectocarpus fasciculatus	NA	44, AF. Peters	REP10-3	14.06.10	Sheltered	None	MCP - qPCR
Ectocarpus fasciculatus	NA	44, AF. Peters	REP10-4	14.06.10	Sheltered	None	MCP - qPCR
Ectocarpus fasciculatus	NA	44, AF. Peters	REP10-5	14.06.10	Sheltered	None	MCP - qPCR
Ectocarpus fasciculatus	NA	50, AF. Peters	TOR08-17C	29.06.08	Sheltered	None	MCP - qPCR
Ectocarpus fasciculatus	NA	56, AF. Peters	(Ec)PHZ1	24.08.06	Sheltered	None	MCP - qPCR
Ectocarpus fasciculatus	NA	56, AF. Peters	(Ec)PHZ29	24.08.06	Sheltered	None	MCP - qPCR
Ectocarpus fasciculatus	NA	56, AF. Peters	(Ec)PHZ36A	26.09.07	Sheltered	None	MCP - qPCR
Ectocarpus fasciculatus	NA	56, AF. Peters	(Ec)PHZ7	24.08.06	Sheltered	None	MCP - qPCR
Ectocarpus fasciculatus	NA	56, AF. Peters	Ec736	21.08.09	Sheltered	None	MCP - qPCR
Ectocarpus fasciculatus	NA	56, AF. Peters	EcPH10-175	15.07.10	Sheltered	None	MCP - qPCR
Ectocarpus fasciculatus	NA	56, AF. Peters	EcPH10-176	15.07.10	Sheltered	None	MCP - qPCR
Ectocarpus fasciculatus	NA	56, AF. Peters	EcPH10-179	15.07.10	Sheltered	None	MCP - qPCR
Ectocarpus fasciculatus	NA	56, AF. Peters	EcPH10-18	14.05.10	Sheltered	None	MCP - qPCR
Ectocarpus fasciculatus	NA	56, AF. Peters	EcPH10-181	15.07.10	Sheltered	None	MCP - qPCR
Ectocarpus fasciculatus	NA	56, AF. Peters	EcPH10-183	15.07.10	Sheltered	None	MCP - qPCR
Ectocarpus fasciculatus	NA	56, AF. Peters	EcPH10-184	15.07.10	Sheltered	None	MCP - qPCR
Ectocarpus fasciculatus	NA	56, AF. Peters	EcPH10-185	15.07.10	Sheltered	None	MCP - qPCR
Ectocarpus fasciculatus	NA	56, AF. Peters	EcPH10-187	15.07.10	Sheltered	None	MCP - qPCR
Ectocarpus fasciculatus	NA	56, AF. Peters	EcPH10-196	15.07.10	Sheltered	None	MCP - qPCR
Ectocarpus fasciculatus	NA	56, AF. Peters	EcPH10-197	15.07.10	Sheltered	None	MCP - qPCR
Ectocarpus fasciculatus	NA	56, AF. Peters	EcPH10-198	15.07.10	Sheltered	None	MCP - qPCR
Ectocarpus fasciculatus	NA	56, AF. Peters	EcPH10-209	15.07.10	Sheltered	None	MCP - qPCR
Ectocarpus fasciculatus	NA	56, AF. Peters	EcPH10-210	15.07.10	Sheltered	None	MCP - qPCR
Ectocarpus fasciculatus	NA	56, AF. Peters	EcPH10-211	15.07.10	Sheltered	None	MCP - qPCR
Ectocarpus fasciculatus	NA	56, AF. Peters	EcPH10-213	15.07.10	Sheltered	None	MCP - qPCR
Ectocarpus fasciculatus	NA	56, AF. Peters	EcPH10-214	15.07.10	Sheltered	None	MCP - qPCR
Ectocarpus fasciculatus	NA	56, AF. Peters	EcPH10-216	15.07.10	Sheltered	None	MCP - qPCR
Ectocarpus fasciculatus	NA	56, AF. Peters	EcPH10-217	15.07.10	Sheltered	None	MCP - qPCR
Ectocarpus fasciculatus	NA	56, AF. Peters	EcPH10-219	15.07.10	Sheltered	None	MCP - qPCR
Ectocarpus fasciculatus	NA	56, AF. Peters	EcPH11-120	02.08.11	Sheltered	None	MCP - qPCR
Ectocarpus fasciculatus	NA	56, AF. Peters	EcPH11-s#2A-10	26.01.11	Sheltered	None	MCP - qPCR
Ectocarpus fasciculatus	NA	56, AF. Peters	EcPH11-s#2A-15	26.01.11	Sheltered	None	MCP - qPCR
Ectocarpus fasciculatus	NA	56, AF. Peters	EcPH11-s#2A-3	26.01.11	Sheltered	None	MCP - qPCR
Ectocarpus fasciculatus	NA	56, AF. Peters	EcPH11-s#2A-30	26.01.11	Sheltered	None	MCP - qPCR
Ectocarpus fasciculatus	NA	56, AF. Peters	EcPH11-s#2A-51	26.01.11	Sheltered	None	MCP - qPCR
Ectocarpus fasciculatus	NA	56, AF. Peters	EcPH11-s#5-2	21.01.11	Sheltered	None	MCP - qPCR
Ectocarpus fasciculatus	NA	56, AF. Peters	EcPH11-s#5-23	21.01.11	Sheltered	None	MCP - qPCR
Ectocarpus fasciculatus	NA	56, AF. Peters	EcPH11-s#5-29	21.01.11	Sheltered	None	MCP - qPCR
Ectocarpus fasciculatus	NA	56, AF. Peters	EcPH11-s#5-45	21.01.11	Sheltered	None	MCP - qPCR
Ectocarpus fasciculatus	NA	55, AF. Peters	Ec328	27.05.06	Sheltered	None	MCP - qPCR
Ectocarpus fasciculatus	NA	59,	EcQB10-14	31.07.10	Sheltered	None	MCP - qPCR
Ectocarpus fasciculatus	NA	59,	EcQB10-17	31.07.10	Sheltered	None	MCP - qPCR
Ectocarpus fasciculatus	NA	59,	EcQB10-27	31.07.10	Sheltered	None	MCP - qPCR
Ectocarpus fasciculatus	NA	59,	EcQB10-29	31.07.10	Sheltered	None	MCP - qPCR
Ectocarpus fasciculatus	NA	59,	EcQB10-3	31.07.10	Sheltered	None	MCP - qPCR
Ectocarpus fasciculatus	NA	59,	EcQB10-31	31.07.10	Sheltered	None	MCP - qPCR
Ectocarpus fasciculatus	NA	59,	EcQB10-33	31.07.10	Sheltered	None	MCP - qPCR
Ectocarpus fasciculatus	NA	59,	EcQB10-34	31.07.10	Sheltered	None	MCP - qPCR
Ectocarpus fasciculatus	NA	59,	EcQB10-4	31.07.10	Sheltered	None	MCP - qPCR
Ectocarpus fasciculatus	NA	59,	EcQB10-7	31.07.10	Sheltered	None	MCP - qPCR
Ectocarpus siliculosus	NA	15, AF. Peters	Ec156	01.03.06	Exposed	A+B	MCP + qPCR
Ectocarpus siliculosus	NA	44, AF. Peters	REP10-59	14.06.10	Exposed	A+B	MCP + qPCR
Ectocarpus siliculosus	NA	44, AF. Peters	REP10-60	14.06.10	Exposed	A+B	MCP + qPCR
Ectocarpus siliculosus	NA	44, AF. Peters	REP10-61	14.06.10	Exposed	A+B	MCP + qPCR
Ectocarpus siliculosus	NA	12, AF. Peters	Ec286	08.03.06	Exposed	A	MCP + qPCR
Ectocarpus siliculosus	NA	12, AF. Peters	Ec287	08.03.06	Exposed	Unknown	MCP + qPCR
Ectocarpus siliculosus	NA	12, AF. Peters	Ec288	08.03.06	Exposed	A	MCP + qPCR
Ectocarpus siliculosus	NA	14, AF. Peters	Ec161	01.03.06	Exposed	A	MCP + qPCR
Ectocarpus siliculosus	NA	15, AF. Peters	Ec157	01.03.06	Exposed	Unknown	MCP + qPCR
Ectocarpus siliculosus	NA	15, AF. Peters	Ec159	02.03.06	Exposed	A	MCP + qPCR
Ectocarpus siliculosus	NA	19, A. Mann	QY24	05.10.06	Exposed	A	MCP + qPCR
Ectocarpus siliculosus	NA	33, AF. Peters	LIA08-4A	15.06.08	Exposed	A	MCP + qPCR
Ectocarpus siliculosus	NA	44, AF. Peters	REP10-56	14.06.10	Exposed	A	MCP + qPCR
Ectocarpus siliculosus	NA	44, AF. Peters	REP10-58	14.06.10	Exposed	A	MCP + qPCR
Ectocarpus siliculosus	NA	44, AF. Peters	REP10-59	14.06.10	Exposed	A+B	MCP + qPCR
Ectocarpus siliculosus	NA	44, AF. Peters	REP10-62	14.06.10	Exposed	A	MCP + qPCR

Ectocarpus siliculosus	NA	44, AF. Peters	REP10-64	14.06.10	Exposed	A	MCP + qPCR
Ectocarpus siliculosus	NA	44, AF. Peters	REP10-65	14.06.10	Exposed	A	MCP + qPCR
Ectocarpus siliculosus	NA	44, AF. Peters	REP10-67	14.06.10	Exposed	A	MCP + qPCR
Ectocarpus siliculosus	NA	44, AF. Peters	REP10-68	14.06.10	Exposed	A	MCP + qPCR
Ectocarpus siliculosus	NA	44, AF. Peters	REP10-69	14.06.10	Exposed	A	MCP + qPCR
Ectocarpus siliculosus	NA	44, AF. Peters	REP10-70	14.06.10	Exposed	A	MCP + qPCR
Ectocarpus siliculosus	NA	44, AF. Peters	REP10-75	14.06.10	Exposed	A	MCP + qPCR
Ectocarpus siliculosus	NA	44, AF. Peters	REP10-77	14.06.10	Exposed	A	MCP + qPCR
Ectocarpus siliculosus	NA	44, AF. Peters	REP10-78	14.06.10	Exposed	A	MCP + qPCR
Ectocarpus siliculosus	NA	44, AF. Peters	REP10-79	14.06.10	Exposed	A	MCP + qPCR
Ectocarpus siliculosus	NA	14, AF. Peters	Ec721	01.03.06	Exposed	None	MCP - qPCR
Ectocarpus siliculosus	NA	32, AF. Peters	WIC08-14C	12.06.08	Exposed	None	MCP - qPCR
Ectocarpus siliculosus	NA	44, AF. Peters	REP10-80	14.06.10	Exposed	None	MCP - qPCR
Ectocarpus siliculosus	NA	47, AF. Peters	W009	04.12.04	Exposed	None	MCP - qPCR
Ectocarpus siliculosus	NA	52, AF. Peters	GOS2b	28.05.08	Exposed	None	MCP - qPCR
Ectocarpus siliculosus	NA	46, AF. Peters	EcQAB10-1	13.06.10	Sheltered	A+B	MCP + qPCR
Ectocarpus siliculosus	NA	46, AF. Peters	EcQAB10-3	13.06.10	Sheltered	A+B	MCP + qPCR
Ectocarpus siliculosus	NA	50, AF. Peters	TOR08-10A	29.06.08	Sheltered	A+B	MCP + qPCR
Ectocarpus siliculosus	NA	50, AF. Peters	TOR08-14C	29.06.08	Sheltered	A+B	MCP + qPCR
Ectocarpus siliculosus	NA	52, AF. Peters	GOS7b	28.05.08	Sheltered	A+B	MCP + qPCR
Ectocarpus siliculosus	NA	53, AF. Peters	LH08-1B	28.05.08	Sheltered	A+B	MCP + qPCR
Ectocarpus siliculosus	NA	53, AF. Peters	LH10b	28.05.08	Sheltered	A+B	MCP + qPCR
Ectocarpus siliculosus	NA	54, AF. Peters	HAS08-17B	30.05.08	Sheltered	A+B	MCP + qPCR
Ectocarpus siliculosus	NA	54, AF. Peters	HAS08-21A	30.05.08	Sheltered	B*	MCP + qPCR
Ectocarpus siliculosus	NA	54, AF. Peters	HAS08-22A	30.05.08	Sheltered	B*	MCP + qPCR
Ectocarpus siliculosus	NA	54, AF. Peters	HAS08-5A	30.05.08	Sheltered	B*	MCP + qPCR
Ectocarpus siliculosus	NA	56, AF. Peters	Ec669	12.08.06	Sheltered	B*	MCP + qPCR
Ectocarpus siliculosus	NA	56, AF. Peters	EcPH10-171	04.07.10	Sheltered	A+B	MCP + qPCR
Ectocarpus siliculosus	NA	56, AF. Peters	EcPH10-9	28.04.10	Sheltered	B*	MCP + qPCR
Ectocarpus siliculosus	NA	56, AF. Peters	EcPH11-107	06.06.11	Sheltered	A+B	MCP + qPCR
Ectocarpus siliculosus	NA	56, AF. Peters	EcPH11-s#2A-16	26.01.11	Sheltered	A+B	MCP + qPCR
Ectocarpus siliculosus	NA	56, AF. Peters	EcPH11-s#2A-37	26.01.11	Sheltered	A+B	MCP + qPCR
Ectocarpus siliculosus	NA	56, AF. Peters	EcPH11-s#2A-41	26.01.11	Sheltered	A+B	MCP + qPCR
Ectocarpus siliculosus	NA	56, AF. Peters	EcPH11-s#2B-8	25.01.11	Sheltered	B*	MCP + qPCR
Ectocarpus siliculosus	NA	55, AF. Peters	EcTH10-18	28.05.10	Sheltered	B*	MCP + qPCR
Ectocarpus siliculosus	NA	55, AF. Peters	EcTH10-205	13.08.10	Sheltered	A+B	MCP + qPCR
Ectocarpus siliculosus	NA	55, AF. Peters	EcTH10-8	28.05.10	Sheltered	B*	MCP + qPCR
Ectocarpus siliculosus	NA	12, AF. Peters	Ec278	08.03.06	Sheltered	A*	MCP + qPCR
Ectocarpus siliculosus	NA	40, AF. Peters	BUT08-12B	02.06.08	Sheltered	B*	MCP + qPCR
Ectocarpus siliculosus	NA	31, AF. Peters	SKY08-13C	14.06.08	Sheltered	A+B	MCP + qPCR
Ectocarpus siliculosus	NA	36, AF. Peters	SAM08-9C	07.06.08	Sheltered	A+B	MCP + qPCR
Ectocarpus siliculosus	NA	36, AF. Peters	SAM4	07.06.08	Sheltered	A+B	MCP + qPCR
Ectocarpus siliculosus	NA	44, AF. Peters	REP10-13	14.06.10	Sheltered	A+B	MCP + qPCR
Ectocarpus siliculosus	NA	46, AF. Peters	EcQAB10-15	13.06.10	Sheltered	A+B	MCP + qPCR
Ectocarpus siliculosus	NA	46, AF. Peters	EcQAB10-16	13.06.10	Sheltered	A+B	MCP + qPCR
Ectocarpus siliculosus	NA	46, AF. Peters	EcQAB10-19	13.06.10	Sheltered	A	MCP + qPCR
Ectocarpus siliculosus	NA	46, AF. Peters	EcQAB10-27	13.06.10	Sheltered	A+B	MCP + qPCR
Ectocarpus siliculosus	NA	46, AF. Peters	EcQAB10-40	13.06.10	Sheltered	A+B	MCP + qPCR
Ectocarpus siliculosus	NA	46, AF. Peters	EcQAB10-46	13.06.10	Sheltered	A+B	MCP + qPCR
Ectocarpus siliculosus	NA	50, AF. Peters	TOR08-9C	29.06.08	Sheltered	A+B	MCP + qPCR
Ectocarpus siliculosus	NA	52, AF. Peters	GOS08-14B	28.05.08	Sheltered	A+B	MCP + qPCR
Ectocarpus siliculosus	NA	52, AF. Peters	GOS08-8C	28.05.08	Sheltered	A+B	MCP + qPCR
Ectocarpus siliculosus	NA	52, AF. Peters	GOS08-9A	28.05.08	Sheltered	B*	MCP + qPCR
Ectocarpus siliculosus	NA	53, AF. Peters	LH12c	28.05.08	Sheltered	B*	MCP + qPCR
Ectocarpus siliculosus	NA	53, AF. Peters	LH2b	28.05.08	Sheltered	A+B	MCP + qPCR
Ectocarpus siliculosus	NA	53, AF. Peters	LH9c	28.05.08	Sheltered	B*	MCP + qPCR
Ectocarpus siliculosus	NA	54, AF. Peters	HAS08-11B	30.05.08	Sheltered	A+B	MCP + qPCR
Ectocarpus siliculosus	NA	54, AF. Peters	HAS08-12B	30.05.08	Sheltered	A+B	MCP + qPCR
Ectocarpus siliculosus	NA	54, AF. Peters	HAS08-13C	30.05.08	Sheltered	A+B	MCP + qPCR
Ectocarpus siliculosus	NA	54, AF. Peters	HAS08-14B	30.05.08	Sheltered	A+B	MCP + qPCR
Ectocarpus siliculosus	NA	54, AF. Peters	HAS08-15A	30.05.08	Sheltered	A+B	MCP + qPCR
Ectocarpus siliculosus	NA	54, AF. Peters	HAS08-16A	30.05.08	Sheltered	A+B	MCP + qPCR
Ectocarpus siliculosus	NA	54, AF. Peters	HAS08-18B	30.05.08	Sheltered	A+B	MCP + qPCR
Ectocarpus siliculosus	NA	54, AF. Peters	HAS08-19B	30.05.08	Sheltered	A+B	MCP + qPCR
Ectocarpus siliculosus	NA	54, AF. Peters	HAS08-20A	30.05.08	Sheltered	A+B	MCP + qPCR
Ectocarpus siliculosus	NA	54, AF. Peters	HAS08-6A	30.05.08	Sheltered	A+B	MCP + qPCR
Ectocarpus siliculosus	NA	54, AF. Peters	HAS08-7A	30.05.08	Sheltered	A+B	MCP + qPCR
Ectocarpus siliculosus	NA	54, AF. Peters	HAS08-8B	30.05.08	Sheltered	A+B	MCP + qPCR
Ectocarpus siliculosus	NA	54, AF. Peters	HAS08-9B	30.05.08	Sheltered	A+B	MCP + qPCR
Ectocarpus siliculosus	NA	54, AF. Peters	HAS12B	30.05.08	Sheltered	A+B	MCP + qPCR
Ectocarpus siliculosus	NA	56, AF. Peters	(Ec)PHS2	26.11.07	Sheltered	A+B	MCP + qPCR
Ectocarpus siliculosus	NA	56, AF. Peters	Ec487	16.08.04	Sheltered	A+B	MCP + qPCR
Ectocarpus siliculosus	NA	56, AF. Peters	Ec673	27.09.07	Sheltered	B*	MCP + qPCR
Ectocarpus siliculosus	NA	56, AF. Peters	Ec730	21.08.09	Sheltered	A+B	MCP + qPCR
Ectocarpus siliculosus	NA	56, AF. Peters	Ec731	21.08.09	Sheltered	A	MCP + qPCR
Ectocarpus siliculosus	NA	56, AF. Peters	Ec732	21.08.09	Sheltered	A+B	MCP + qPCR
Ectocarpus siliculosus	NA	56, AF. Peters	EcPH10-127	04.07.10	Sheltered	A+B	MCP + qPCR
Ectocarpus siliculosus	NA	56, AF. Peters	EcPH10-128	04.07.10	Sheltered	B	MCP + qPCR

Ectocarpus siliculosus	NA	56, AF. Peters	EcPH10-129	04.07.10	Sheltered	A+B	MCP + qPCR
Ectocarpus siliculosus	NA	56, AF. Peters	EcPH10-132	04.07.10	Sheltered	A+B	MCP + qPCR
Ectocarpus siliculosus	NA	56, AF. Peters	EcPH10-134	04.07.10	Sheltered	A+B	MCP + qPCR
Ectocarpus siliculosus	NA	56, AF. Peters	EcPH10-139	04.07.10	Sheltered	A+B	MCP + qPCR
Ectocarpus siliculosus	NA	56, AF. Peters	EcPH10-173	04.07.10	Sheltered	A+B	MCP + qPCR
Ectocarpus siliculosus	NA	56, AF. Peters	EcPH10-292	12.08.10	Sheltered	A+B	MCP + qPCR
Ectocarpus siliculosus	NA	56, AF. Peters	EcPH11-100	06.06.11	Sheltered	A+B	MCP + qPCR
Ectocarpus siliculosus	NA	56, AF. Peters	EcPH11-108	06.06.11	Sheltered	A+B	MCP + qPCR
Ectocarpus siliculosus	NA	56, AF. Peters	EcPH11-115	03.07.11	Sheltered	B*	MCP + qPCR
Ectocarpus siliculosus	NA	56, AF. Peters	EcPH11-116	03.07.11	Sheltered	A+B	MCP + qPCR
Ectocarpus siliculosus	NA	56, AF. Peters	EcPH11-117	03.07.11	Sheltered	B*	MCP + qPCR
Ectocarpus siliculosus	NA	56, AF. Peters	EcPH11-118	03.07.11	Sheltered	B*	MCP + qPCR
Ectocarpus siliculosus	NA	56, AF. Peters	EcPH11-s#2A-4	26.01.11	Sheltered	A+B	MCP + qPCR
Ectocarpus siliculosus	NA	56, AF. Peters	EcPH11-s#2A-40	26.01.11	Sheltered	A+B	MCP + qPCR
Ectocarpus siliculosus	NA	56, AF. Peters	EcPH11-s#2A-47	26.01.11	Sheltered	A+B	MCP + qPCR
Ectocarpus siliculosus	NA	56, AF. Peters	EcPH11-s#2A-48	26.01.11	Sheltered	A+B	MCP + qPCR
Ectocarpus siliculosus	NA	56, AF. Peters	EcPH11-s#2A-50	26.01.11	Sheltered	A+B	MCP + qPCR
Ectocarpus siliculosus	NA	56, AF. Peters	EcPH11-s#2A-52	26.01.11	Sheltered	B*	MCP + qPCR
Ectocarpus siliculosus	NA	56, AF. Peters	EcPH11-s#2B-15	25.01.11	Sheltered	B*	MCP + qPCR
Ectocarpus siliculosus	NA	56, AF. Peters	EcPH11-s#2B-19	25.01.11	Sheltered	A+B	MCP + qPCR
Ectocarpus siliculosus	NA	56, AF. Peters	EcPH11-s#2B-41	25.01.11	Sheltered	B*	MCP + qPCR
Ectocarpus siliculosus	NA	56, AF. Peters	EcPH11-s#5-17	21.01.11	Sheltered	A+B	MCP + qPCR
Ectocarpus siliculosus	NA	56, AF. Peters	EcPH11-s#5-25	21.01.11	Sheltered	A+B	MCP + qPCR
Ectocarpus siliculosus	NA	56, AF. Peters	EcPH11-s#5-30	21.01.11	Sheltered	A*	MCP + qPCR
Ectocarpus siliculosus	NA	56, AF. Peters	EcPH11-s#5-33	21.01.11	Sheltered	A*	MCP + qPCR
Ectocarpus siliculosus	NA	55, AF. Peters	Ec539	24.05.05	Sheltered	A+B	MCP + qPCR
Ectocarpus siliculosus	NA	55, AF. Peters	EcTH10-10	28.05.10	Sheltered	B*	MCP + qPCR
Ectocarpus siliculosus	NA	55, AF. Peters	EcTH10-17	28.05.10	Sheltered	B*	MCP + qPCR
Ectocarpus siliculosus	NA	55, AF. Peters	EcTH10-176	13.08.10	Sheltered	A+B	MCP + qPCR
Ectocarpus siliculosus	NA	59,	EcQB10-21	31.07.10	Sheltered	A+B	MCP + qPCR
Ectocarpus siliculosus	NA	59,	EcQB10-22	31.07.10	Sheltered	A+B	MCP + qPCR
Ectocarpus siliculosus	NA	59,	EcQB10-23	31.07.10	Sheltered	A+B	MCP + qPCR
Ectocarpus siliculosus	NA	NA	(Ec)BIOCEAN	28.03.08	Sheltered	A+B	MCP + qPCR
Ectocarpus siliculosus	NA	12, AF. Peters	Ec201	08.03.06	Sheltered	Unknown	MCP + qPCR
Ectocarpus siliculosus	NA	12, AF. Peters	Ec202	08.03.06	Sheltered	Unknown	MCP + qPCR
Ectocarpus siliculosus	NA	12, AF. Peters	Ec246	08.03.06	Sheltered	A	MCP + qPCR
Ectocarpus siliculosus	NA	12, AF. Peters	Ec266	08.03.06	Sheltered	Unknown	MCP + qPCR
Ectocarpus siliculosus	NA	12, AF. Peters	Ec267	08.03.06	Sheltered	A	MCP + qPCR
Ectocarpus siliculosus	NA	12, AF. Peters	Ec269	08.03.06	Sheltered	A	MCP + qPCR
Ectocarpus siliculosus	NA	12, AF. Peters	Ec270	08.03.06	Sheltered	Unknown	MCP + qPCR
Ectocarpus siliculosus	NA	12, AF. Peters	Ec274	08.03.06	Sheltered	Unknown	MCP + qPCR
Ectocarpus siliculosus	NA	12, AF. Peters	Ec283	08.03.06	Sheltered	A	MCP + qPCR
Ectocarpus siliculosus	NA	12, AF. Peters	Ec284	08.03.06	Sheltered	A	MCP + qPCR
Ectocarpus siliculosus	NA	12, AF. Peters	Ec285	08.03.06	Sheltered	A	MCP + qPCR
Ectocarpus siliculosus	NA	12, AF. Peters	Ec289	08.03.06	Sheltered	Unknown	MCP + qPCR
Ectocarpus siliculosus	NA	12, AF. Peters	Ec290	08.03.06	Sheltered	A	MCP + qPCR
Ectocarpus siliculosus	NA	12, AF. Peters	Ec291	08.03.06	Sheltered	A	MCP + qPCR
Ectocarpus siliculosus	NA	13, AF. Peters	Ec294	06.03.06	Sheltered	A	MCP + qPCR
Ectocarpus siliculosus	NA	13, AF. Peters	Ec295	06.03.06	Sheltered	A	MCP + qPCR
Ectocarpus siliculosus	NA	13, AF. Peters	Ec296	06.03.06	Sheltered	A	MCP + qPCR
Ectocarpus siliculosus	NA	13, AF. Peters	Ec297	06.03.06	Sheltered	A	MCP + qPCR
Ectocarpus siliculosus	NA	13, AF. Peters	Ec298	06.03.06	Sheltered	A	MCP + qPCR
Ectocarpus siliculosus	NA	13, AF. Peters	EC298	06.03.06	Sheltered	A	MCP + qPCR
Ectocarpus siliculosus	NA	13, AF. Peters	Ec299	06.03.06	Sheltered	A	MCP + qPCR
Ectocarpus siliculosus	NA	13, AF. Peters	Ec300	06.03.06	Sheltered	A	MCP + qPCR
Ectocarpus siliculosus	NA	13, AF. Peters	Ec302	06.03.06	Sheltered	A	MCP + qPCR
Ectocarpus siliculosus	NA	13, AF. Peters	Ec304	06.03.06	Sheltered	A	MCP + qPCR
Ectocarpus siliculosus	NA	13, AF. Peters	Ec305	06.03.06	Sheltered	A	MCP + qPCR
Ectocarpus siliculosus	NA	13, AF. Peters	Ec306	06.03.06	Sheltered	A	MCP + qPCR
Ectocarpus siliculosus	NA	13, AF. Peters	Ec307	06.03.06	Sheltered	A	MCP + qPCR
Ectocarpus siliculosus	NA	16, AF. Peters	Ec147	28.02.06	Sheltered	A	MCP + qPCR
Ectocarpus siliculosus	NA	16, AF. Peters	Ec150	28.02.06	Sheltered	B	MCP + qPCR
Ectocarpus siliculosus	NA	16, AF. Peters	Ec151	28.02.06	Sheltered	B	MCP + qPCR
Ectocarpus siliculosus	NA	16, AF. Peters	Ec153	28.02.06	Sheltered	B	MCP + qPCR
Ectocarpus siliculosus	NA	16, AF. Peters	Ec154	28.02.06	Sheltered	B	MCP + qPCR
Ectocarpus siliculosus	NA	16, AF. Peters	Ec155	28.02.06	Sheltered	B	MCP + qPCR
Ectocarpus siliculosus	NA	36, AF. Peters	Ob07-10A	07.11.07	Sheltered	A	MCP + qPCR
Ectocarpus siliculosus	NA	36, AF. Peters	Ob07-15A	07.11.07	Sheltered	A	MCP + qPCR
Ectocarpus siliculosus	NA	36, AF. Peters	Ob07-4	07.11.07	Sheltered	A	MCP + qPCR
Ectocarpus siliculosus	NA	36, AF. Peters	SAM08-5A	07.06.08	Sheltered	A	MCP + qPCR
Ectocarpus siliculosus	NA	43, AF. Peters	RHOS08-12C	20.06.08	Sheltered	A	MCP + qPCR
Ectocarpus siliculosus	NA	44, AF. Peters	REP10-18	14.06.10	Sheltered	A	MCP + qPCR
Ectocarpus siliculosus	NA	44, AF. Peters	REP10-22	14.06.10	Sheltered	A	MCP + qPCR
Ectocarpus siliculosus	NA	44, AF. Peters	REP10-23	14.06.10	Sheltered	A	MCP + qPCR
Ectocarpus siliculosus	NA	44, AF. Peters	REP10-26	14.06.10	Sheltered	B	MCP + qPCR
Ectocarpus siliculosus	NA	44, AF. Peters	REP10-28	14.06.10	Sheltered	A	MCP + qPCR
Ectocarpus siliculosus	NA	44, AF. Peters	REP10-30	14.06.10	Sheltered	A	MCP + qPCR
Ectocarpus siliculosus	NA	44, AF. Peters	REP10-32	14.06.10	Sheltered	A	MCP + qPCR

Ectocarpus siliculosus	NA	44, AF. Peters	REP10-35	14.06.10	Sheltered	Unknown	MCP + qPCR
Ectocarpus siliculosus	NA	44, AF. Peters	REP10-36	14.06.10	Sheltered	A	MCP + qPCR
Ectocarpus siliculosus	NA	44, AF. Peters	REP10-38	14.06.10	Sheltered	A	MCP + qPCR
Ectocarpus siliculosus	NA	44, AF. Peters	REP10-42	14.06.10	Sheltered	A	MCP + qPCR
Ectocarpus siliculosus	NA	44, AF. Peters	REP10-45	14.06.10	Sheltered	A	MCP + qPCR
Ectocarpus siliculosus	NA	44, AF. Peters	REP10-47	14.06.10	Sheltered	A	MCP + qPCR
Ectocarpus siliculosus	NA	44, AF. Peters	REP10-48	14.06.10	Sheltered	A	MCP + qPCR
Ectocarpus siliculosus	NA	44, AF. Peters	REP10-50	14.06.10	Sheltered	A	MCP + qPCR
Ectocarpus siliculosus	NA	44, AF. Peters	REP10-51	14.06.10	Sheltered	A	MCP + qPCR
Ectocarpus siliculosus	NA	44, AF. Peters	REP10-52	14.06.10	Sheltered	A	MCP + qPCR
Ectocarpus siliculosus	NA	44, AF. Peters	REP10-54	14.06.10	Sheltered	A	MCP + qPCR
Ectocarpus siliculosus	NA	46, AF. Peters	EcQAB10-10	13.06.10	Sheltered	A	MCP + qPCR
Ectocarpus siliculosus	NA	46, AF. Peters	EcQAB10-11	13.06.10	Sheltered	A	MCP + qPCR
Ectocarpus siliculosus	NA	46, AF. Peters	EcQAB10-13	13.06.10	Sheltered	A	MCP + qPCR
Ectocarpus siliculosus	NA	46, AF. Peters	EcQAB10-14	13.06.10	Sheltered	A	MCP + qPCR
Ectocarpus siliculosus	NA	46, AF. Peters	EcQAB10-17	13.06.10	Sheltered	Unknown	MCP + qPCR
Ectocarpus siliculosus	NA	46, AF. Peters	EcQAB10-18	13.06.10	Sheltered	A	MCP + qPCR
Ectocarpus siliculosus	NA	46, AF. Peters	EcQAB10-2	13.06.10	Sheltered	A	MCP + qPCR
Ectocarpus siliculosus	NA	46, AF. Peters	EcQAB10-20	13.06.10	Sheltered	A	MCP + qPCR
Ectocarpus siliculosus	NA	46, AF. Peters	EcQAB10-24	13.06.10	Sheltered	A	MCP + qPCR
Ectocarpus siliculosus	NA	46, AF. Peters	EcQAB10-25	13.06.10	Sheltered	A	MCP + qPCR
Ectocarpus siliculosus	NA	46, AF. Peters	EcQAB10-28	13.06.10	Sheltered	A	MCP + qPCR
Ectocarpus siliculosus	NA	46, AF. Peters	EcQAB10-29	13.06.10	Sheltered	A	MCP + qPCR
Ectocarpus siliculosus	NA	46, AF. Peters	EcQAB10-30	13.06.10	Sheltered	A	MCP + qPCR
Ectocarpus siliculosus	NA	46, AF. Peters	EcQAB10-32	13.06.10	Sheltered	A	MCP + qPCR
Ectocarpus siliculosus	NA	46, AF. Peters	EcQAB10-33	13.06.10	Sheltered	A	MCP + qPCR
Ectocarpus siliculosus	NA	46, AF. Peters	EcQAB10-34	13.06.10	Sheltered	A	MCP + qPCR
Ectocarpus siliculosus	NA	46, AF. Peters	EcQAB10-35	13.06.10	Sheltered	A	MCP + qPCR
Ectocarpus siliculosus	NA	46, AF. Peters	EcQAB10-38	13.06.10	Sheltered	A	MCP + qPCR
Ectocarpus siliculosus	NA	46, AF. Peters	EcQAB10-39	13.06.10	Sheltered	A	MCP + qPCR
Ectocarpus siliculosus	NA	46, AF. Peters	EcQAB10-43	13.06.10	Sheltered	A	MCP + qPCR
Ectocarpus siliculosus	NA	46, AF. Peters	EcQAB10-48	13.06.10	Sheltered	A	MCP + qPCR
Ectocarpus siliculosus	NA	46, AF. Peters	EcQAB10-5	13.06.10	Sheltered	A	MCP + qPCR
Ectocarpus siliculosus	NA	46, AF. Peters	EcQAB10-6	13.06.10	Sheltered	A	MCP + qPCR
Ectocarpus siliculosus	NA	46, AF. Peters	EcQAB10-7	13.06.10	Sheltered	A	MCP + qPCR
Ectocarpus siliculosus	NA	46, AF. Peters	EcQAB10-8	13.06.10	Sheltered	A	MCP + qPCR
Ectocarpus siliculosus	NA	46, AF. Peters	EcQAB10-9	13.06.10	Sheltered	A	MCP + qPCR
Ectocarpus siliculosus	NA	46, AF. Peters	QAB08-1C	24.05.08	Sheltered	A	MCP + qPCR
Ectocarpus siliculosus	NA	46, AF. Peters	QAB08-2A	24.05.08	Sheltered	A	MCP + qPCR
Ectocarpus siliculosus	NA	46, AF. Peters	QAB08-3A	24.05.08	Sheltered	A	MCP + qPCR
Ectocarpus siliculosus	NA	50, AF. Peters	TOR08-13B	29.06.08	Sheltered	A	MCP + qPCR
Ectocarpus siliculosus	NA	52, AF. Peters	GOS08-11B	28.05.08	Sheltered	A	MCP + qPCR
Ectocarpus siliculosus	NA	52, AF. Peters	GOS08-12B	28.05.08	Sheltered	A	MCP + qPCR
Ectocarpus siliculosus	NA	52, AF. Peters	GOS08-13B	28.05.08	Sheltered	A	MCP + qPCR
Ectocarpus siliculosus	NA	52, AF. Peters	GOS08-17B	28.05.08	Sheltered	A	MCP + qPCR
Ectocarpus siliculosus	NA	52, AF. Peters	GOS5c	28.05.08	Sheltered	A	MCP + qPCR
Ectocarpus siliculosus	NA	52, AF. Peters	GOS6c	28.05.08	Sheltered	A	MCP + qPCR
Ectocarpus siliculosus	NA	53, AF. Peters	LH11c	28.05.08	Sheltered	Unknown	MCP + qPCR
Ectocarpus siliculosus	NA	53, AF. Peters	LH5c	28.05.08	Sheltered	A	MCP + qPCR
Ectocarpus siliculosus	NA	56, AF. Peters	(Ec)PHL21	08.09.06	Sheltered	A	MCP + qPCR
Ectocarpus siliculosus	NA	56, AF. Peters	(Ec)PHS1	12.08.06	Sheltered	A	MCP + qPCR
Ectocarpus siliculosus	NA	56, AF. Peters	(Ec)PHU(07)2	27.09.07	Sheltered	A	MCP + qPCR
Ectocarpus siliculosus	NA	56, AF. Peters	Ec670	12.08.06	Sheltered	A	MCP + qPCR
Ectocarpus siliculosus	NA	56, AF. Peters	Ec671	26.09.07	Sheltered	Unknown	MCP + qPCR
Ectocarpus siliculosus	NA	56, AF. Peters	EcPH10-11	14.05.10	Sheltered	B	MCP + qPCR
Ectocarpus siliculosus	NA	56, AF. Peters	EcPH10-130	04.07.10	Sheltered	A	MCP + qPCR
Ectocarpus siliculosus	NA	56, AF. Peters	EcPH10-131	04.07.10	Sheltered	A	MCP + qPCR
Ectocarpus siliculosus	NA	56, AF. Peters	EcPH10-142	04.07.10	Sheltered	A	MCP + qPCR
Ectocarpus siliculosus	NA	56, AF. Peters	EcPH10-15	14.05.10	Sheltered	A	MCP + qPCR
Ectocarpus siliculosus	NA	56, AF. Peters	EcPH10-151	04.07.10	Sheltered	Unknown	MCP + qPCR
Ectocarpus siliculosus	NA	56, AF. Peters	EcPH10-152	04.07.10	Sheltered	Unknown	MCP + qPCR
Ectocarpus siliculosus	NA	56, AF. Peters	EcPH10-157	04.07.10	Sheltered	A	MCP + qPCR
Ectocarpus siliculosus	NA	56, AF. Peters	EcPH10-160	04.07.10	Sheltered	A	MCP + qPCR
Ectocarpus siliculosus	NA	56, AF. Peters	EcPH10-162	04.07.10	Sheltered	Unknown	MCP + qPCR
Ectocarpus siliculosus	NA	56, AF. Peters	EcPH10-165	04.07.10	Sheltered	A	MCP + qPCR
Ectocarpus siliculosus	NA	56, AF. Peters	EcPH10-166	04.07.10	Sheltered	Unknown	MCP + qPCR
Ectocarpus siliculosus	NA	56, AF. Peters	EcPH10-167	04.07.10	Sheltered	Unknown	MCP + qPCR
Ectocarpus siliculosus	NA	56, AF. Peters	EcPH10-168	04.07.10	Sheltered	A	MCP + qPCR
Ectocarpus siliculosus	NA	56, AF. Peters	EcPH10-170	04.07.10	Sheltered	A	MCP + qPCR
Ectocarpus siliculosus	NA	56, AF. Peters	EcPH10-294	12.08.10	Sheltered	Unknown	MCP + qPCR
Ectocarpus siliculosus	NA	56, AF. Peters	EcPH11-109	06.06.11	Sheltered	Unknown	MCP + qPCR
Ectocarpus siliculosus	NA	56, AF. Peters	EcPH11-124	02.08.11	Sheltered	A	MCP + qPCR
Ectocarpus siliculosus	NA	56, AF. Peters	EcPH11-8	20.01.11	Sheltered	B	MCP + qPCR
Ectocarpus siliculosus	NA	56, AF. Peters	EcPH11-97	06.06.11	Sheltered	A	MCP + qPCR
Ectocarpus siliculosus	NA	56, AF. Peters	EcPH11-98	06.06.11	Sheltered	A	MCP + qPCR
Ectocarpus siliculosus	NA	56, AF. Peters	EcPH11-99	06.06.11	Sheltered	A	MCP + qPCR
Ectocarpus siliculosus	NA	56, AF. Peters	EcPH11-s#2A-17	26.01.11	Sheltered	Unknown	MCP + qPCR
Ectocarpus siliculosus	NA	56, AF. Peters	EcPH11-s#2A-32	26.01.11	Sheltered	A	MCP + qPCR

Ectocarpus siliculosus	NA	56, AF. Peters	EcPH11-s#2A-35	26.01.11	Sheltered	A	MCP + qPCR
Ectocarpus siliculosus	NA	56, AF. Peters	EcPH11-s#2A-39	26.01.11	Sheltered	Unknown	MCP + qPCR
Ectocarpus siliculosus	NA	56, AF. Peters	EcPH11-s#2A-49	26.01.11	Sheltered	A	MCP + qPCR
Ectocarpus siliculosus	NA	56, AF. Peters	EcPH11-s#2A-6	26.01.11	Sheltered	A	MCP + qPCR
Ectocarpus siliculosus	NA	56, AF. Peters	EcPH11-s#2A-9	26.01.11	Sheltered	A	MCP + qPCR
Ectocarpus siliculosus	NA	56, AF. Peters	EcPH11-s#2B-12	25.01.11	Sheltered	A	MCP + qPCR
Ectocarpus siliculosus	NA	56, AF. Peters	EcPH11-s#2B-38	25.01.11	Sheltered	A	MCP + qPCR
Ectocarpus siliculosus	NA	56, AF. Peters	EcPH11-s#2B-40	25.01.11	Sheltered	Unknown	MCP + qPCR
Ectocarpus siliculosus	NA	56, AF. Peters	EcPH11-s#2B-43	25.01.11	Sheltered	A	MCP + qPCR
Ectocarpus siliculosus	NA	56, AF. Peters	EcPH11-s#2B-45	25.01.11	Sheltered	A	MCP + qPCR
Ectocarpus siliculosus	NA	56, AF. Peters	EcPH11-s#2B-46	25.01.11	Sheltered	A	MCP + qPCR
Ectocarpus siliculosus	NA	56, AF. Peters	EcPH11-s#2B-48	25.01.11	Sheltered	A	MCP + qPCR
Ectocarpus siliculosus	NA	56, AF. Peters	EcPH11-s#2B-5	25.01.11	Sheltered	A	MCP + qPCR
Ectocarpus siliculosus	NA	56, AF. Peters	EcPH11-s#2B-6	25.01.11	Sheltered	A	MCP + qPCR
Ectocarpus siliculosus	NA	56, AF. Peters	EcPH11-s#5-1	21.01.11	Sheltered	Unknown	MCP + qPCR
Ectocarpus siliculosus	NA	56, AF. Peters	EcPH11-s#5-13	21.01.11	Sheltered	Unknown	MCP + qPCR
Ectocarpus siliculosus	NA	56, AF. Peters	EcPH11-s#5-15	21.01.11	Sheltered	A	MCP + qPCR
Ectocarpus siliculosus	NA	56, AF. Peters	EcPH11-s#5-18	21.01.11	Sheltered	A	MCP + qPCR
Ectocarpus siliculosus	NA	56, AF. Peters	EcPH11-s#5-19	21.01.11	Sheltered	A	MCP + qPCR
Ectocarpus siliculosus	NA	56, AF. Peters	EcPH11-s#5-20	21.01.11	Sheltered	A	MCP + qPCR
Ectocarpus siliculosus	NA	56, AF. Peters	EcPH11-s#5-28	21.01.11	Sheltered	Unknown	MCP + qPCR
Ectocarpus siliculosus	NA	56, AF. Peters	EcPH11-s#5-31	21.01.11	Sheltered	A	MCP + qPCR
Ectocarpus siliculosus	NA	56, AF. Peters	EcPH11-s#5-32	21.01.11	Sheltered	Unknown	MCP + qPCR
Ectocarpus siliculosus	NA	56, AF. Peters	EcPH11-s#5-34	21.01.11	Sheltered	Unknown	MCP + qPCR
Ectocarpus siliculosus	NA	56, AF. Peters	EcPH11-s#5-35	21.01.11	Sheltered	A	MCP + qPCR
Ectocarpus siliculosus	NA	56, AF. Peters	EcPH11-s#5-37	21.01.11	Sheltered	B	MCP + qPCR
Ectocarpus siliculosus	NA	56, AF. Peters	EcPH11-s#5-42	21.01.11	Sheltered	Unknown	MCP + qPCR
Ectocarpus siliculosus	NA	56, AF. Peters	EcPH11-s#5-43	21.01.11	Sheltered	Unknown	MCP + qPCR
Ectocarpus siliculosus	NA	56, AF. Peters	EcPH11-s#5-49	21.01.11	Sheltered	B	MCP + qPCR
Ectocarpus siliculosus	NA	56, AF. Peters	EcPH11-s#5-5	21.01.11	Sheltered	A	MCP + qPCR
Ectocarpus siliculosus	NA	56, AF. Peters	EcPH11-s#5-6	21.01.11	Sheltered	A	MCP + qPCR
Ectocarpus siliculosus	NA	56, AF. Peters	EcPH11-s#5-8	21.01.11	Sheltered	B	MCP + qPCR
Ectocarpus siliculosus	NA	55, AF. Peters	Ec531	24.05.05	Sheltered	A	MCP + qPCR
Ectocarpus siliculosus	NA	55, AF. Peters	Ec533	24.05.05	Sheltered	Unknown	MCP + qPCR
Ectocarpus siliculosus	NA	55, AF. Peters	Ec540	24.05.05	Sheltered	A	MCP + qPCR
Ectocarpus siliculosus	NA	55, AF. Peters	Ec543	22.08.05	Sheltered	A	MCP + qPCR
Ectocarpus siliculosus	NA	55, AF. Peters	EcTH10-172	13.08.10	Sheltered	Unknown	MCP + qPCR
Ectocarpus siliculosus	NA	55, AF. Peters	EcTH10-175	13.08.10	Sheltered	A	MCP + qPCR
Ectocarpus siliculosus	NA	55, AF. Peters	EcTH10-197	13.08.10	Sheltered	A	MCP + qPCR
Ectocarpus siliculosus	NA	55, AF. Peters	EcTH10-198	13.08.10	Sheltered	A	MCP + qPCR
Ectocarpus siliculosus	NA	55, AF. Peters	EcTH10-199	13.08.10	Sheltered	A	MCP + qPCR
Ectocarpus siliculosus	NA	55, AF. Peters	EcTH10-201	13.08.10	Sheltered	A	MCP + qPCR
Ectocarpus siliculosus	NA	55, AF. Peters	EcTH10-202	13.08.10	Sheltered	A	MCP + qPCR
Ectocarpus siliculosus	NA	55, AF. Peters	EcTH10-9	28.05.10	Sheltered	A	MCP + qPCR
Ectocarpus siliculosus	NA	59,	EcQB10-1	31.07.10	Sheltered	A	MCP + qPCR
Ectocarpus siliculosus	NA	59,	EcQB10-16	31.07.10	Sheltered	A	MCP + qPCR
Ectocarpus siliculosus	NA	59,	EcQB10-26	31.07.10	Sheltered	Unknown	MCP + qPCR
Ectocarpus siliculosus	NA	59,	EcQB10-8	31.07.10	Sheltered	A	MCP + qPCR
Ectocarpus siliculosus	NA	61, AF. Peters	ET08-2A	15.04.08	Sheltered	A	MCP + qPCR
Ectocarpus siliculosus	NA	61, AF. Peters	ET08-3A	15.04.08	Sheltered	A	MCP + qPCR
Ectocarpus siliculosus	NA	61, AF. Peters	ET08-4B	15.04.08	Sheltered	A	MCP + qPCR
Ectocarpus siliculosus	NA	61, AF. Peters	ET08-5B	15.04.08	Sheltered	A	MCP + qPCR
Ectocarpus siliculosus	NA	11, AF. Peters	Ec311	07.03.06	Sheltered	None	MCP - qPCR
Ectocarpus siliculosus	NA	11, AF. Peters	Ec312	07.03.06	Sheltered	None	MCP - qPCR
Ectocarpus siliculosus	NA	11, AF. Peters	Ec314	07.03.06	Sheltered	None	MCP - qPCR
Ectocarpus siliculosus	NA	12, AF. Peters	Ec247	08.03.06	Sheltered	None	MCP - qPCR
Ectocarpus siliculosus	NA	12, AF. Peters	Ec275	08.03.06	Sheltered	None	MCP - qPCR
Ectocarpus siliculosus	NA	12, AF. Peters	Ec276	08.03.06	Sheltered	None	MCP - qPCR
Ectocarpus siliculosus	NA	12, AF. Peters	Ec277	08.03.06	Sheltered	None	MCP - qPCR
Ectocarpus siliculosus	NA	12, AF. Peters	Ec279	08.03.06	Sheltered	None	MCP - qPCR
Ectocarpus siliculosus	NA	12, AF. Peters	Ec280	08.03.06	Sheltered	None	MCP - qPCR
Ectocarpus siliculosus	NA	12, AF. Peters	Ec281	08.03.06	Sheltered	None	MCP - qPCR
Ectocarpus siliculosus	NA	12, AF. Peters	Ec282	08.03.06	Sheltered	None	MCP - qPCR
Ectocarpus siliculosus	NA	12, AF. Peters	Ec292	08.03.06	Sheltered	None	MCP - qPCR
Ectocarpus siliculosus	NA	12, AF. Peters	Ec293	08.03.06	Sheltered	None	MCP - qPCR
Ectocarpus siliculosus	NA	12, AF. Peters	Ec315	07.03.06	Sheltered	None	MCP - qPCR
Ectocarpus siliculosus	NA	12, AF. Peters	Ec316	07.03.06	Sheltered	None	MCP - qPCR
Ectocarpus siliculosus	NA	13, AF. Peters	Ec301	06.03.06	Sheltered	None	MCP - qPCR
Ectocarpus siliculosus	NA	13, AF. Peters	Ec303	06.03.06	Sheltered	None	MCP - qPCR
Ectocarpus siliculosus	NA	44, AF. Peters	REP10-1	14.06.10	Sheltered	None	MCP - qPCR
Ectocarpus siliculosus	NA	44, AF. Peters	REP10-10	14.06.10	Sheltered	None	MCP - qPCR
Ectocarpus siliculosus	NA	44, AF. Peters	REP10-11	14.06.10	Sheltered	None	MCP - qPCR
Ectocarpus siliculosus	NA	44, AF. Peters	REP10-12	14.06.10	Sheltered	None	MCP - qPCR
Ectocarpus siliculosus	NA	44, AF. Peters	REP10-14	14.06.10	Sheltered	None	MCP - qPCR
Ectocarpus siliculosus	NA	44, AF. Peters	REP10-16	14.06.10	Sheltered	None	MCP - qPCR
Ectocarpus siliculosus	NA	44, AF. Peters	REP10-17	14.06.10	Sheltered	None	MCP - qPCR
Ectocarpus siliculosus	NA	44, AF. Peters	REP10-19	14.06.10	Sheltered	None	MCP - qPCR
Ectocarpus siliculosus	NA	44, AF. Peters	REP10-21	14.06.10	Sheltered	None	MCP - qPCR

Ectocarpus siliculosus	NA	44, AF. Peters	REP10-25	14.06.10	Sheltered	None	MCP - qPCR
Ectocarpus siliculosus	NA	44, AF. Peters	REP10-27	14.06.10	Sheltered	None	MCP - qPCR
Ectocarpus siliculosus	NA	44, AF. Peters	REP10-29	14.06.10	Sheltered	None	MCP - qPCR
Ectocarpus siliculosus	NA	44, AF. Peters	REP10-33	14.06.10	Sheltered	None	MCP - qPCR
Ectocarpus siliculosus	NA	44, AF. Peters	REP10-34	14.06.10	Sheltered	None	MCP - qPCR
Ectocarpus siliculosus	NA	44, AF. Peters	REP10-39	14.06.10	Sheltered	None	MCP - qPCR
Ectocarpus siliculosus	NA	44, AF. Peters	REP10-40	14.06.10	Sheltered	None	MCP - qPCR
Ectocarpus siliculosus	NA	44, AF. Peters	REP10-43	14.06.10	Sheltered	None	MCP - qPCR
Ectocarpus siliculosus	NA	44, AF. Peters	REP10-44	14.06.10	Sheltered	None	MCP - qPCR
Ectocarpus siliculosus	NA	44, AF. Peters	REP10-46	14.06.10	Sheltered	None	MCP - qPCR
Ectocarpus siliculosus	NA	44, AF. Peters	REP10-53	14.06.10	Sheltered	None	MCP - qPCR
Ectocarpus siliculosus	NA	44, AF. Peters	REP10-6	14.06.10	Sheltered	None	MCP - qPCR
Ectocarpus siliculosus	NA	44, AF. Peters	REP10-7	14.06.10	Sheltered	None	MCP - qPCR
Ectocarpus siliculosus	NA	46, AF. Peters	EcQAB10-21	13.06.10	Sheltered	None	MCP - qPCR
Ectocarpus siliculosus	NA	46, AF. Peters	EcQAB10-22	13.06.10	Sheltered	None	MCP - qPCR
Ectocarpus siliculosus	NA	46, AF. Peters	EcQAB10-23	13.06.10	Sheltered	None	MCP - qPCR
Ectocarpus siliculosus	NA	46, AF. Peters	EcQAB10-36	13.06.10	Sheltered	None	MCP - qPCR
Ectocarpus siliculosus	NA	46, AF. Peters	EcQAB10-37	13.06.10	Sheltered	None	MCP - qPCR
Ectocarpus siliculosus	NA	46, AF. Peters	EcQAB10-41	13.06.10	Sheltered	None	MCP - qPCR
Ectocarpus siliculosus	NA	46, AF. Peters	EcQAB10-42	13.06.10	Sheltered	None	MCP - qPCR
Ectocarpus siliculosus	NA	46, AF. Peters	EcQAB10-44	13.06.10	Sheltered	None	MCP - qPCR
Ectocarpus siliculosus	NA	46, AF. Peters	EcQAB10-45	13.06.10	Sheltered	None	MCP - qPCR
Ectocarpus siliculosus	NA	46, AF. Peters	EcQAB10-47	13.06.10	Sheltered	None	MCP - qPCR
Ectocarpus siliculosus	NA	52, AF. Peters	GOS08-16B	28.05.08	Sheltered	None	MCP - qPCR
Ectocarpus siliculosus	NA	53, AF. Peters	LH3C	28.05.08	Sheltered	None	MCP - qPCR
Ectocarpus siliculosus	NA	53, AF. Peters	LH4A	28.05.08	Sheltered	None	MCP - qPCR
Ectocarpus siliculosus	NA	56, AF. Peters	Ec331	05.06.07	Sheltered	None	MCP - qPCR
Ectocarpus siliculosus	NA	56, AF. Peters	Ec393	01.07.03	Sheltered	None	MCP - qPCR
Ectocarpus siliculosus	NA	56, AF. Peters	Ec488	16.08.04	Sheltered	None	MCP - qPCR
Ectocarpus siliculosus	NA	56, AF. Peters	Ec489	29.08.04	Sheltered	None	MCP - qPCR
Ectocarpus siliculosus	NA	56, AF. Peters	Ec697	16.04.07	Sheltered	None	MCP - qPCR
Ectocarpus siliculosus	NA	56, AF. Peters	EcPH10-12	14.05.10	Sheltered	None	MCP - qPCR
Ectocarpus siliculosus	NA	56, AF. Peters	EcPH10-133	04.07.10	Sheltered	None	MCP - qPCR
Ectocarpus siliculosus	NA	56, AF. Peters	EcPH10-135	04.07.10	Sheltered	None	MCP - qPCR
Ectocarpus siliculosus	NA	56, AF. Peters	EcPH10-137	04.07.10	Sheltered	None	MCP - qPCR
Ectocarpus siliculosus	NA	56, AF. Peters	EcPH10-140	04.07.10	Sheltered	None	MCP - qPCR
Ectocarpus siliculosus	NA	56, AF. Peters	EcPH10-141	04.07.10	Sheltered	None	MCP - qPCR
Ectocarpus siliculosus	NA	56, AF. Peters	EcPH10-143	04.07.10	Sheltered	None	MCP - qPCR
Ectocarpus siliculosus	NA	56, AF. Peters	EcPH10-144	04.07.10	Sheltered	None	MCP - qPCR
Ectocarpus siliculosus	NA	56, AF. Peters	EcPH10-145	04.07.10	Sheltered	None	MCP - qPCR
Ectocarpus siliculosus	NA	56, AF. Peters	EcPH10-146	04.07.10	Sheltered	None	MCP - qPCR
Ectocarpus siliculosus	NA	56, AF. Peters	EcPH10-147	04.07.10	Sheltered	None	MCP - qPCR
Ectocarpus siliculosus	NA	56, AF. Peters	EcPH10-148	04.07.10	Sheltered	None	MCP - qPCR
Ectocarpus siliculosus	NA	56, AF. Peters	EcPH10-149	04.07.10	Sheltered	None	MCP - qPCR
Ectocarpus siliculosus	NA	56, AF. Peters	EcPH10-153	04.07.10	Sheltered	None	MCP - qPCR
Ectocarpus siliculosus	NA	56, AF. Peters	EcPH10-154	04.07.10	Sheltered	None	MCP - qPCR
Ectocarpus siliculosus	NA	56, AF. Peters	EcPH10-156	04.07.10	Sheltered	None	MCP - qPCR
Ectocarpus siliculosus	NA	56, AF. Peters	EcPH10-158	04.07.10	Sheltered	None	MCP - qPCR
Ectocarpus siliculosus	NA	56, AF. Peters	EcPH10-159	04.07.10	Sheltered	None	MCP - qPCR
Ectocarpus siliculosus	NA	56, AF. Peters	EcPH10-16	14.05.06	Sheltered	None	MCP - qPCR
Ectocarpus siliculosus	NA	56, AF. Peters	EcPH10-161	04.07.10	Sheltered	None	MCP - qPCR
Ectocarpus siliculosus	NA	56, AF. Peters	EcPH10-164	04.07.10	Sheltered	None	MCP - qPCR
Ectocarpus siliculosus	NA	56, AF. Peters	EcPH10-17	14.05.10	Sheltered	None	MCP - qPCR
Ectocarpus siliculosus	NA	56, AF. Peters	EcPH10-172	04.07.10	Sheltered	None	MCP - qPCR
Ectocarpus siliculosus	NA	56, AF. Peters	EcPH10-174	04.07.10	Sheltered	None	MCP - qPCR
Ectocarpus siliculosus	NA	56, AF. Peters	EcPH10-20	14.05.10	Sheltered	None	MCP - qPCR
Ectocarpus siliculosus	NA	56, AF. Peters	EcPH10-21	14.05.10	Sheltered	None	MCP - qPCR
Ectocarpus siliculosus	NA	56, AF. Peters	EcPH10-291	12.08.10	Sheltered	None	MCP - qPCR
Ectocarpus siliculosus	NA	56, AF. Peters	EcPH10-293	12.08.10	Sheltered	None	MCP - qPCR
Ectocarpus siliculosus	NA	56, AF. Peters	EcPH10-296	12.08.10	Sheltered	None	MCP - qPCR
Ectocarpus siliculosus	NA	56, AF. Peters	EcPH10-38	27.05.10	Sheltered	None	MCP - qPCR
Ectocarpus siliculosus	NA	56, AF. Peters	EcPH10-40	27.05.10	Sheltered	None	MCP - qPCR
Ectocarpus siliculosus	NA	56, AF. Peters	EcPH10-41	27.05.10	Sheltered	None	MCP - qPCR
Ectocarpus siliculosus	NA	56, AF. Peters	EcPH10-43	27.05.10	Sheltered	None	MCP - qPCR
Ectocarpus siliculosus	NA	56, AF. Peters	EcPH10-44	27.05.10	Sheltered	None	MCP - qPCR
Ectocarpus siliculosus	NA	56, AF. Peters	EcPH11-102	06.06.11	Sheltered	None	MCP - qPCR
Ectocarpus siliculosus	NA	56, AF. Peters	EcPH11-103	06.06.11	Sheltered	None	MCP - qPCR
Ectocarpus siliculosus	NA	56, AF. Peters	EcPH11-104	06.06.11	Sheltered	None	MCP - qPCR
Ectocarpus siliculosus	NA	56, AF. Peters	EcPH11-105	06.06.11	Sheltered	None	MCP - qPCR
Ectocarpus siliculosus	NA	56, AF. Peters	EcPH11-110	06.06.11	Sheltered	None	MCP - qPCR
Ectocarpus siliculosus	NA	56, AF. Peters	EcPH11-121	02.08.11	Sheltered	None	MCP - qPCR
Ectocarpus siliculosus	NA	56, AF. Peters	EcPH11-s#2A-12	26.01.11	Sheltered	None	MCP - qPCR
Ectocarpus siliculosus	NA	56, AF. Peters	EcPH11-s#2A-20	26.01.11	Sheltered	None	MCP - qPCR
Ectocarpus siliculosus	NA	56, AF. Peters	EcPH11-s#2A-22	26.01.11	Sheltered	None	MCP - qPCR
Ectocarpus siliculosus	NA	56, AF. Peters	EcPH11-s#2A-28	26.01.11	Sheltered	None	MCP - qPCR
Ectocarpus siliculosus	NA	56, AF. Peters	EcPH11-s#2A-38	26.01.11	Sheltered	None	MCP - qPCR
Ectocarpus siliculosus	NA	56, AF. Peters	EcPH11-s#2A-43	26.01.11	Sheltered	None	MCP - qPCR
Ectocarpus siliculosus	NA	56, AF. Peters	EcPH11-s#2A-45	26.01.11	Sheltered	None	MCP - qPCR

Ectocarpus siliculosus	NA	56, AF. Peters	EcPH11-s#2B-13	25.01.11	Sheltered	None	MCP - qPCR
Ectocarpus siliculosus	NA	56, AF. Peters	EcPH11-s#2B-14	25.01.11	Sheltered	None	MCP - qPCR
Ectocarpus siliculosus	NA	56, AF. Peters	EcPH11-s#2B-18	25.01.11	Sheltered	None	MCP - qPCR
Ectocarpus siliculosus	NA	56, AF. Peters	EcPH11-s#2B-51	25.01.11	Sheltered	None	MCP - qPCR
Ectocarpus siliculosus	NA	56, AF. Peters	EcPH11-s#5-10	21.01.11	Sheltered	None	MCP - qPCR
Ectocarpus siliculosus	NA	56, AF. Peters	EcPH11-s#5-11	21.01.11	Sheltered	None	MCP - qPCR
Ectocarpus siliculosus	NA	56, AF. Peters	EcPH11-s#5-12	21.01.11	Sheltered	None	MCP - qPCR
Ectocarpus siliculosus	NA	56, AF. Peters	EcPH11-s#5-14	21.01.11	Sheltered	None	MCP - qPCR
Ectocarpus siliculosus	NA	56, AF. Peters	EcPH11-s#5-21	21.01.11	Sheltered	None	MCP - qPCR
Ectocarpus siliculosus	NA	56, AF. Peters	EcPH11-s#5-22	21.01.11	Sheltered	None	MCP - qPCR
Ectocarpus siliculosus	NA	56, AF. Peters	EcPH11-s#5-36	21.01.11	Sheltered	None	MCP - qPCR
Ectocarpus siliculosus	NA	56, AF. Peters	EcPH11-s#5-39	21.01.11	Sheltered	None	MCP - qPCR
Ectocarpus siliculosus	NA	56, AF. Peters	EcPH11-s#5-4	21.01.11	Sheltered	None	MCP - qPCR
Ectocarpus siliculosus	NA	56, AF. Peters	EcPH11-s#5-40	21.01.11	Sheltered	None	MCP - qPCR
Ectocarpus siliculosus	NA	56, AF. Peters	EcPH11-s#5-48	21.01.11	Sheltered	None	MCP - qPCR
Ectocarpus siliculosus	NA	56, AF. Peters	EcPH11-s#5-50	21.01.11	Sheltered	None	MCP - qPCR
Ectocarpus siliculosus	NA	56, AF. Peters	EcPH11-s#5-51	21.01.11	Sheltered	None	MCP - qPCR
Ectocarpus siliculosus	NA	56, AF. Peters	EcPH11-s#5-9	21.01.11	Sheltered	None	MCP - qPCR
Ectocarpus siliculosus	NA	55, AF. Peters	Ec327	27.05.06	Sheltered	None	MCP - qPCR
Ectocarpus siliculosus	NA	55, AF. Peters	Ec530	24.05.05	Sheltered	None	MCP - qPCR
Ectocarpus siliculosus	NA	55, AF. Peters	Ec534	24.05.05	Sheltered	None	MCP - qPCR
Ectocarpus siliculosus	NA	55, AF. Peters	Ec535	24.05.05	Sheltered	None	MCP - qPCR
Ectocarpus siliculosus	NA	55, AF. Peters	Ec536	24.05.05	Sheltered	None	MCP - qPCR
Ectocarpus siliculosus	NA	55, AF. Peters	Ec538	24.05.05	Sheltered	None	MCP - qPCR
Ectocarpus siliculosus	NA	55, AF. Peters	Ec542	24.05.05	Sheltered	None	MCP - qPCR
Ectocarpus siliculosus	NA	55, AF. Peters	Ec544	22.08.05	Sheltered	None	MCP - qPCR
Ectocarpus siliculosus	NA	55, AF. Peters	Ec546	22.08.05	Sheltered	None	MCP - qPCR
Ectocarpus siliculosus	NA	55, AF. Peters	EcTH10-1	28.05.10	Sheltered	None	MCP - qPCR
Ectocarpus siliculosus	NA	55, AF. Peters	EcTH10-11	28.05.10	Sheltered	None	MCP - qPCR
Ectocarpus siliculosus	NA	55, AF. Peters	EcTH10-12	28.05.10	Sheltered	None	MCP - qPCR
Ectocarpus siliculosus	NA	55, AF. Peters	EcTH10-13	28.05.10	Sheltered	None	MCP - qPCR
Ectocarpus siliculosus	NA	55, AF. Peters	EcTH10-14	28.05.10	Sheltered	None	MCP - qPCR
Ectocarpus siliculosus	NA	55, AF. Peters	EcTH10-15	28.05.10	Sheltered	None	MCP - qPCR
Ectocarpus siliculosus	NA	55, AF. Peters	EcTH10-16	28.05.10	Sheltered	None	MCP - qPCR
Ectocarpus siliculosus	NA	55, AF. Peters	EcTH10-170	13.08.10	Sheltered	None	MCP - qPCR
Ectocarpus siliculosus	NA	55, AF. Peters	EcTH10-171	13.08.10	Sheltered	None	MCP - qPCR
Ectocarpus siliculosus	NA	55, AF. Peters	EcTH10-173	13.08.10	Sheltered	None	MCP - qPCR
Ectocarpus siliculosus	NA	55, AF. Peters	EcTH10-174	13.08.10	Sheltered	None	MCP - qPCR
Ectocarpus siliculosus	NA	55, AF. Peters	EcTH10-177	13.08.10	Sheltered	None	MCP - qPCR
Ectocarpus siliculosus	NA	55, AF. Peters	EcTH10-178	13.08.10	Sheltered	None	MCP - qPCR
Ectocarpus siliculosus	NA	55, AF. Peters	EcTH10-2	28.05.10	Sheltered	None	MCP - qPCR
Ectocarpus siliculosus	NA	55, AF. Peters	EcTH10-200	13.08.10	Sheltered	None	MCP - qPCR
Ectocarpus siliculosus	NA	55, AF. Peters	EcTH10-203	13.08.10	Sheltered	None	MCP - qPCR
Ectocarpus siliculosus	NA	55, AF. Peters	EcTH10-204	13.08.10	Sheltered	None	MCP - qPCR
Ectocarpus siliculosus	NA	55, AF. Peters	EcTH10-206	13.08.10	Sheltered	None	MCP - qPCR
Ectocarpus siliculosus	NA	55, AF. Peters	EcTH10-3	28.05.10	Sheltered	None	MCP - qPCR
Ectocarpus siliculosus	NA	55, AF. Peters	EcTH10-4	28.05.10	Sheltered	None	MCP - qPCR
Ectocarpus siliculosus	NA	55, AF. Peters	EcTH10-5	28.05.10	Sheltered	None	MCP - qPCR
Ectocarpus siliculosus	NA	55, AF. Peters	EcTH10-6	28.05.10	Sheltered	None	MCP - qPCR
Ectocarpus siliculosus	NA	55, AF. Peters	EcTH10-7	28.05.10	Sheltered	None	MCP - qPCR
Ectocarpus siliculosus	NA	60, AF. Peters	Ec547	26.04.05	Sheltered	None	MCP - qPCR
Ectocarpus siliculosus	NA	60, AF. Peters	Ec548	26.04.05	Sheltered	None	MCP - qPCR
Ectocarpus siliculosus	NA	60, AF. Peters	Ec549	26.04.05	Sheltered	None	MCP - qPCR
Ectocarpus siliculosus	NA	60, AF. Peters	Ec550	26.04.05	Sheltered	None	MCP - qPCR
Ectocarpus siliculosus	NA	60, AF. Peters	Ec551	26.04.05	Sheltered	None	MCP - qPCR
Ectocarpus siliculosus	NA	60, D. Müller	Ec552	10.04.05	Sheltered	None	MCP - qPCR
Ectocarpus siliculosus	NA	59,	EcQB10-19	31.07.10	Sheltered	None	MCP - qPCR
Ectocarpus siliculosus	NA	59,	EcQB10-20	31.07.10	Sheltered	None	MCP - qPCR
Ectocarpus siliculosus	NA	59,	EcQB10-24	31.07.10	Sheltered	None	MCP - qPCR
Ectocarpus siliculosus	NA	59,	EcQB10-25	31.07.10	Sheltered	None	MCP - qPCR
Ectocarpus sp.	NA	40, AF. Peters	BUT08-8C	01.06.08	Exposed	A*	MCP + qPCR
Ectocarpus sp.	NA	34, AF. Peters	RAT08-6A	11.06.08	Exposed	A	MCP + qPCR
Ectocarpus sp.	NA	46, AF. Peters	QAB08-5D	24.05.08	Sheltered	B*	MCP + qPCR
Ectocarpus sp.	NA	53, AF. Peters	LH7c	28.05.08	Sheltered	A+B	MCP + qPCR
Ectocarpus sp.	NA	36, AF. Peters	SAM08-11C	07.06.08	Sheltered	B	MCP + qPCR
Ectocarpus sp.	NA	36, AF. Peters	SAM08-3B	07.06.08	Sheltered	B	MCP + qPCR
Ectocarpus sp.	NA	36, AF. Peters	SAM11C4	07.06.08	Sheltered	B	MCP + qPCR
Ectocarpus sp.	NA	46, AF. Peters	EcQAB10-26	13.06.10	Sheltered	B	MCP + qPCR
Ectocarpus sp.	NA	53, AF. Peters	LH14a	28.05.08	Sheltered	A	MCP + qPCR
Ectocarpus sp.	NA	36, AF. Peters	SAM08-10C	07.06.08	Sheltered	None	MCP - qPCR
Ectocarpus sp.	NA	39, AF. Peters	POR08-1B	17.06.08	Sheltered	None	MCP - qPCR
Ectocarpus sp.	NA	39, AF. Peters	POR08-2B	17.06.08	Sheltered	None	MCP - qPCR
Ectocarpus sp.	NA	46, AF. Peters	EcQAB10-31	13.06.10	Sheltered	None	MCP - qPCR
Kuckuckia sp.	NA	14, AF. Peters	Ec160	01.03.06	Sheltered	Unknown	MCP + qPCR
Laminaria digitata	G	56, AF. Peters	LdigPH10-1	11.8.10	Sheltered	Unknown	MCP -
Laminaria digitata	G	56, AF. Peters	LdigPH10-2	11.8.10	Sheltered	Unknown	MCP -
Laminaria digitata	G	56, AF. Peters	LdigPH10-3	11.8.10	Sheltered	Unknown	MCP -
Laminaria digitata	G	56, AF. Peters	LdigPH10-4	11.8.10	Sheltered	Unknown	MCP -

Laminaria digitata	G	56, AF. Peters	LdigPH10-5	11.8.10	Sheltered	Unknown	MCP -
Laminaria digitata	G	56, AF. Peters	LdigPH10-6	11.8.10	Sheltered	Unknown	MCP +
Laminaria digitata	G	56, AF. Peters	LdigPH10-7	11.8.10	Sheltered	Unknown	MCP -
Laminaria digitata	G	56, AF. Peters	LdigPH10-8	11.8.10	Sheltered	Unknown	MCP -
Laminaria digitata	G	56, AF. Peters	LdigPH10-9	11.8.10	Sheltered	Unknown	MCP -
Laminaria digitata	G	56, AF. Peters	LdigPH10-10	11.8.10	Sheltered	Unknown	MCP +
Laminaria digitata	G	56, AF. Peters	LdigPH10-11	11.8.10	Sheltered	Unknown	MCP +
Laminaria digitata	G	56, AF. Peters	LdigPH10-12	11.8.10	Sheltered	Unknown	MCP -
Laminaria digitata	G	56, AF. Peters	LdigPH10-13	11.8.10	Sheltered	Unknown	MCP -
Laminaria digitata	G	56, AF. Peters	LdigPH10-14	11.8.10	Sheltered	Unknown	MCP -
Laminaria digitata	G	56, AF. Peters	LdigPH10-15	11.8.10	Sheltered	Unknown	MCP -
Laminaria digitata	G	56, AF. Peters	LdigPH10-16	11.8.10	Sheltered	Unknown	MCP -
Laminaria digitata	G	56, AF. Peters	LdigPH10-17	11.8.10	Sheltered	Unknown	MCP -
Laminaria digitata	G	56, AF. Peters	LdigPH10-18	11.8.10	Sheltered	Unknown	MCP +<br>CKP296732<br>KP296730
Laminaria digitata	G	56, AF. Peters	LdigPH10-19	11.8.10	Sheltered	Unknown	MCP -
Laminaria digitata	G	56, AF. Peters	LdigPH10-20	11.8.10	Sheltered	Unknown	MCP -
Laminaria digitata	G	56, AF. Peters	LdigPH10-21	11.8.10	Sheltered	Unknown	MCP +
Laminaria digitata	G	56, AF. Peters	LdigPH10-22	11.8.10	Sheltered	Unknown	MCP +
Laminaria digitata	G	56, AF. Peters	LdigPH10-23	11.8.10	Sheltered	Unknown	MCP -
Laminaria digitata	G	56, AF. Peters	LdigPH10-24	11.8.10	Sheltered	Unknown	MCP +
Laminaria digitata	G	56, AF. Peters	LdigPH10-25	11.8.10	Sheltered	Unknown	MCP +
Laminaria digitata	G	56, AF. Peters	LdigPH10-26	11.8.10	Sheltered	Unknown	MCP -
Laminaria digitata	G	56, AF. Peters	LdigPH10-27	11.8.10	Sheltered	Unknown	MCP -
Laminaria digitata	G	56, AF. Peters	LdigPH10-28	11.8.10	Sheltered	Unknown	MCP -
Laminaria digitata	G	56, AF. Peters	LdigPH10-29	11.8.10	Sheltered	Unknown	MCP -
Laminaria digitata	G	56, AF. Peters	LdigPH10-30	11.8.10	Sheltered	Unknown	MCP +<br>CKY316507
Laminaria digitata	G	56, AF. Peters	LdigPH10-31	11.8.10	Sheltered	Unknown	MCP +
Laminaria digitata	G	56, AF. Peters	LdigPH10-32	11.8.10	Sheltered	Unknown	MCP -
Laminaria digitata	G	56, AF. Peters	LdigPH10-33	11.8.10	Sheltered	Unknown	MCP +
Laminaria digitata	G	56, AF. Peters	LdigPH10-34	11.8.10	Sheltered	Unknown	MCP -
Laminaria digitata	G	56, AF. Peters	LdigPH10-35	11.8.10	Sheltered	Unknown	MCP -
Laminaria digitata	G	56, AF. Peters	LdigPH10-36	11.8.10	Sheltered	Unknown	MCP -
Laminaria digitata	G	56, AF. Peters	LdigPH10-37	11.8.10	Sheltered	Unknown	MCP -
Laminaria digitata	G	56, AF. Peters	LdigPH10-38	11.8.10	Sheltered	Unknown	MCP -
Laminaria digitata	G	56, AF. Peters	LdigPH10-39	11.8.10	Sheltered	Unknown	MCP +
Laminaria digitata	G	56, AF. Peters	LdigPH10-40	11.8.10	Sheltered	Unknown	MCP +
Laminaria digitata	G	56, AF. Peters	LdigPH10-41	11.8.10	Sheltered	Unknown	MCP -
Laminaria digitata	G	56, AF. Peters	LdigPH10-42	11.8.10	Sheltered	Unknown	MCP -
Laminaria digitata	G	56, AF. Peters	LdigPH10-43	11.8.10	Sheltered	Unknown	MCP -
Laminaria digitata	G	56, AF. Peters	LdigPH10-44	11.8.10	Sheltered	Unknown	MCP -
Laminaria digitata	G	56, AF. Peters	LdigPH10-45	11.8.10	Sheltered	Unknown	MCP -
Laminaria digitata	G	56, AF. Peters	LdigPH10-46	11.8.10	Sheltered	Unknown	MCP -
Laminaria digitata	G	56, AF. Peters	LdigPH10-47	11.8.10	Sheltered	Unknown	MCP -
Laminaria digitata	G	56, AF. Peters	LdigPH10-48	11.8.10	Sheltered	Unknown	MCP -
Laminaria hyperborea	G	56, AF. Peters	LhypPH10-1	07.11.10	Sheltered	Unknown	MCP -
Laminaria hyperborea	G	56, AF. Peters	LhypPH10-3	07.11.10	Sheltered	Unknown	CKP296733
Laminaria hyperborea	G	56, AF. Peters	LhypPH10-4	07.11.10	Sheltered	Unknown	MCP -
Laminaria hyperborea	G	56, AF. Peters	LhypPH10-6	07.11.10	Sheltered	Unknown	MCP -
Laminaria hyperborea	G	56, AF. Peters	LhypPH10-7	07.11.10	Sheltered	Unknown	MCP +
Laminaria hyperborea	G	56, AF. Peters	LhypPH10-8	07.11.10	Sheltered	Unknown	MCP +
Laminaria hyperborea	G	56, AF. Peters	LhypPH10-9	07.11.10	Sheltered	Unknown	MCP -
Laminaria hyperborea	G	56, AF. Peters	LhypPH10-11	07.11.10	Sheltered	Unknown	MCP -
Laminaria hyperborea	G	56, AF. Peters	LhypPH11-1	2011	Sheltered	Unknown	MCP -
Laminaria hyperborea	G	56, AF. Peters	LhypPH11-2	2011	Sheltered	Unknown	MCP -
Saccharina latissima	G	56, AF. Peters	SlatPH10-1	07.11.10	Sheltered	Unknown	MCP -
Saccharina latissima	G	56, AF. Peters	SlatPH10-2	07.11.10	Sheltered	Unknown	MCP +
Saccharina latissima	G	56, AF. Peters	SlatPH10-3	07.11.10	Sheltered	Unknown	MCP -
Saccharina latissima	G	56, AF. Peters	SlatPH10-4	07.11.10	Sheltered	Unknown	MCP -
Saccharina latissima	G	56, AF. Peters	SlatPH10-5	07.11.10	Sheltered	Unknown	MCP -
Saccharina latissima	G	56, AF. Peters	SlatPH10-6	07.11.10	Sheltered	Unknown	MCP -
Saccharina latissima	G	56, AF. Peters	SlatPH10-7	07.11.10	Sheltered	Unknown	CKP296731<br>KP296734
Saccharina latissima	G	56, AF. Peters	SlatPH10-8	07.11.10	Sheltered	Unknown	MCP -
Saccharina latissima	G	56, AF. Peters	SlatPH10-9	07.11.10	Sheltered	Unknown	MCP -
Saccharina latissima	G	56, AF. Peters	SlatPH10-10	07.11.10	Sheltered	Unknown	MCP +
Saccharina latissima	G	56, AF. Peters	SlatPH10-11	07.11.10	Sheltered	Unknown	MCP -
Saccharina latissima	G	56, AF. Peters	SlatPH10-12	07.11.10	Sheltered	Unknown	MCP -
Saccharina latissima	G	56, AF. Peters	SlatPH10-13	07.11.10	Sheltered	Unknown	MCP -
Saccharina latissima	G	56, AF. Peters	SlatPH10-14	07.11.10	Sheltered	Unknown	MCP +
Saccharina latissima	G	56, AF. Peters	SlatPH10-15	07.11.10	Sheltered	Unknown	MCP -
Saccharina latissima	G	56, AF. Peters	SlatPH10-16	07.11.10	Sheltered	Unknown	MCP -
Saccharina latissima	G	56, AF. Peters	SlatPH10-17	07.11.10	Sheltered	Unknown	MCP -
Saccharina latissima	G	56, AF. Peters	SlatPH10-18	07.11.10	Sheltered	Unknown	MCP -
Saccharina latissima	G	56, AF. Peters	SlatPH10-19	07.11.10	Sheltered	Unknown	MCP -
Saccharina latissima	G	56, AF. Peters	SlatPH10-20	07.11.10	Sheltered	Unknown	MCP +

Saccharina latissima	G	56, AF. Peters	SlatPH10-21	07.11.10	Sheltered	Unknown	MCP -
Saccharina latissima	G	56, AF. Peters	SlatPH10-22	07.11.10	Sheltered	Unknown	MCP -
Saccharina latissima	G	56, AF. Peters	SlatPH10-23	07.11.10	Sheltered	Unknown	MCP -
Saccharina latissima	G	56, AF. Peters	SlatPH10-24	07.11.10	Sheltered	Unknown	MCP -
Ectocarpus sp.	NA	24, Müller et al	NA	1995-1997	NA	-	Gp-1<br>260 +<br>45 -
Ectocarpus sp.	NA	74, Müller et al	NA	1995-1997	NA	-	Gp-1<br>230 +<br>35 -
Ectocarpus siliculosus	NA	10, Sengco et al	Sam120	1988	NA	-	Gp-1 +
Ectocarpus siliculosus	NA	25, Sengco et al	PuySp	1985	NA	-	Gp-1 +
Ectocarpus siliculosus	NA	25, Sengco et al	PuyZ9	1985	NA	-	Gp-1 +
Ectocarpus siliculosus	NA	66, Sengco et al	Cph40a	1979	NA	-	Gp-1 +
Ectocarpus siliculosus	NA	66, Sengco et al	Cph40-1	1979	NA	-	Gp-1 +
Ectocarpus siliculosus	NA	71, Sengco et al	NapEA	1965	NA	-	Gp-1 +
Ectocarpus siliculosus	NA	87, Sengco et al	SAF28	1993	NA	-	Gp-1 -
Ectocarpus siliculosus	NA	2, Sengco et al	NFL31-K1	1977	NA	-	Gp-1 -
Ectocarpus siliculosus	NA	57, Sengco et al	Ros70Z-35	1970	NA	-	Gp-1 +
Ectocarpus siliculosus	NA	18, Sengco et al	Coq6a	1991	NA	-	Gp-1 -
Ectocarpus siliculosus	NA	23, Sengco et al	CH19a	1984	NA	-	Gp-1 +
Ectocarpus siliculosus	NA	100, Sengco et al	Jap16-Z10	1989	NA	-	Gp-1 -
Ectocarpus siliculosus	NA	104, Sengco et al	Vic11a2	1988	NA	-	Gp-1 -
Ectocarpus siliculosus	NA	104, Sengco et al	Vic12-1	1988	NA	-	Gp-1 -
Ectocarpus siliculosus	NA	3, Sengco et al	Pen2a	1975	NA	-	Gp-1 +
Ectocarpus siliculosus	NA	3, Sengco et al	Pen4a	1975	NA	-	Gp-1 -
Ectocarpus siliculosus	NA	3, Sengco et al	Pen5a	1975	NA	-	Gp-1 -
Ectocarpus siliculosus	NA	3, Sengco et al	Pen9d	1975	NA	-	Gp-1 -
Ectocarpus siliculosus	NA	3, Sengco et al	Pen10b	1975	NA	-	Gp-1 +
Ectocarpus siliculosus	NA	3, Sengco et al	Pen15a	1975	NA	-	Gp-1 -
Ectocarpus siliculosus	NA	5, Sengco et al	Bft23a	1978	NA	-	Gp-1 +
Ectocarpus siliculosus	NA	4, Sengco et al	Wil11a	1978	NA	-	Gp-1 +
Ectocarpus siliculosus	NA	6, Sengco et al	Tam2b	1978	NA	-	Gp-1 -
Ectocarpus siliculosus	NA	7, Sengco et al	Par10n	1978	NA	-	Gp-1 -
Ectocarpus siliculosus	NA	7, Sengco et al	Par27a	1978	NA	-	Gp-1 -
Ectocarpus siliculosus	NA	106, Sengco et al	NZ4a3	NA	NA	-	Gp-1 -
Ectocarpus siliculosus	NA	106, Sengco et al	NZ15-d2	NA	NA	-	Gp-1 +
Ectocarpus siliculosus	NA	104, Sengco et al	Vic1a8	1977	NA	-	Gp-1 +
Ectocarpus siliculosus	NA	104, Sengco et al	Vic7b	1977	NA	-	Gp-1 -
Ectocarpus siliculosus	NA	63, Sengco et al	Tro2b	1978	NA	-	Gp-1 +
Ectocarpus siliculosus	NA	64,	Ber42	1977	NA	-	Gp-1 +
Ectocarpus siliculosus	NA	64,	Ber21	1977	NA	-	Gp-1 -
Ectocarpus siliculosus	NA	62,	VF01	1971	NA	-	Gp-1 +
Ectocarpus siliculosus	NA	71, Sengco et al	NapR-B1	1959	NA	-	Gp-1 +
Ectocarpus siliculosus	NA	71, Sengco et al	NapD-A2	1965	NA	-	Gp-1 +
Ectocarpus siliculosus	NA	71, Sengco et al	Nap70	1975	NA	-	Gp-1 -
Ectocarpus siliculosus	NA	71, Sengco et al	Nap84	1975	NA	-	Gp-1 -
Ectocarpus siliculosus	NA	1, Sengco et al	GroG30	1973	NA	-	Gp-1 -
Ectocarpus siliculosus	NA	Irish Sea, Sengco et al	MorC	1970	NA	-	Gp-1 +
Ectocarpus siliculosus	NA	Irish Sea, Sengco et al	BHI689	1989	NA	-	Gp-1 -
Ectocarpus siliculosus	NA	English Channel	1983,LH2	1983	NA	-	Gp-1 +
Ectocarpus siliculosus	NA	67,	Syl1a	1978	NA	-	Gp-1 -
Ectocarpus siliculosus	NA	68,	Hel34-1	1977	NA	-	Gp-1 -
Ectocarpus siliculosus	NA	57, Sengco et al	Ros23-7	1993	NA	-	Gp-1 +
Ectocarpus siliculosus	NA	65, Sengco et al	Grh6c	1979	NA	-	Gp-1 +
Ectocarpus siliculosus	NA	65, Sengco et al	Kal1c	1979	NA	-	Gp-1 +
Ectocarpus siliculosus	NA	65, Sengco et al	Ska21b	1979	NA	-	Gp-1 +
Ectocarpus siliculosus	NA	70, Sengco et al	Sp1	1960	NA	-	Gp-1 -
Ectocarpus siliculosus	NA	73, Sengco et al	CI17-2	1991	NA	-	Gp-1 -
Ectocarpus siliculosus	NA	73, Sengco et al	CI44-1	1991	NA	-	Gp-1 -
Ectocarpus siliculosus	NA	73, Sengco et al	CI104-1	1991	NA	-	Gp-1 -
Ectocarpus siliculosus	NA	81, Sengco et al	SAf91-1	1991	NA	-	Gp-1 -
Ectocarpus siliculosus	NA	87, Sengco et al	SAf25-1	1993	NA	-	Gp-1 +
Ectocarpus siliculosus	NA	87, Sengco et al	SAf26-1	1993	NA	-	Gp-1 -
Ectocarpus siliculosus	NA	87, Sengco et al	SAf31-1	1993	NA	-	Gp-1 +
Ectocarpus siliculosus	NA	87, Sengco et al	SAf32-1	1993	NA	-	Gp-1 +
Ectocarpus siliculosus	NA	87, Sengco et al	SAf33-1	1993	NA	-	Gp-1 -
Ectocarpus siliculosus	NA	90, Sengco et al	SAf49-1	NA	NA	-	Gp-1 -
Ectocarpus siliculosus	NA	9, Sengco et al	SF19a	1977	NA	-	Gp-1 +
Ectocarpus siliculosus	NA	8, Sengco et al	SBML3a	1977	NA	-	Gp-1 +
Ectocarpus siliculosus	NA	8, Sengco et al	SBML4a	1977	NA	-	Gp-1 +
Ectocarpus siliculosus	NA	8, Sengco et al	SBML7-1	1977	NA	-	Gp-1 -
Ectocarpus siliculosus	NA	21, Sengco et al	RC32-4	1991	NA	-	Gp-1 -
Ectocarpus siliculosus	NA	21, Sengco et al	RC33-1	1991	NA	-	Gp-1 -
Ectocarpus siliculosus	NA	21, Sengco et al	RC61-5	1991	NA	-	Gp-1 -
Ectocarpus siliculosus	NA	27, Sengco et al	SAm76-1	1989	NA	-	Gp-1 +

Ectocarpus siliculosus	NA	28, Sengco et al	SAm81-1	1989	NA	-	Gp-1 -
Ectocarpus siliculosus	NA	28, Sengco et al	SAm86-a1	1989	NA	-	Gp-1 +
Ectocarpus siliculosus	NA	28, Sengco et al	SAm111	1989	NA	-	Gp-1 +
Ectocarpus siliculosus	NA	29, Sengco et al	SAm99-1	1989	NA	-	Gp-1 -
Ectocarpus siliculosus	NA	29, Sengco et al	SAm100-1	1989	NA	-	Gp-1 +
Ectocarpus siliculosus	NA	29, Sengco et al	SAm109-1	1989	NA	-	Gp-1 -
Ectocarpus siliculosus	NA	30, Sengco et al	SAm117-1	1989	NA	-	Gp-1 -
Ectocarpus siliculosus	NA	30, Sengco et al	SAm117-4	1989	NA	-	Gp-1 +
Ectocarpus siliculosus	NA	100, Sengco et al	Jap19	1989	NA	-	Gp-1 -
Ectocarpus siliculosus	NA	94 P., Sengco et al	Jap91-5	1991	NA	-	Gp-1 -
Ectocarpus siliculosus	NA	94 P., Sengco et al	Jap91-6	1991	NA	-	Gp-1 -
Ectocarpus siliculosus	NA	94 P., Sengco et al	Jap91-7	1991	NA	-	Gp-1 -
Ectocarpus siliculosus	NA	94 P., Sengco et al	Jap91-8	1991	NA	-	Gp-1 -
Ectocarpus siliculosus	NA	92 P., Sengco et al	Jap91-12	1991	NA	-	Gp-1 -
Ectocarpus siliculosus	NA	92 P., Sengco et al	Jap91-13	1991	NA	-	Gp-1 +
Ectocarpus siliculosus	NA	95, Sengco et al	Jap55-1	1993	NA	-	Gp-1 -
Ectocarpus siliculosus	NA	104, Sengco et al	Vic88-19-5	1988	NA	-	Gp-1 +
Ectocarpus siliculosus	NA	104, Sengco et al	Vic88-20-1	1988	NA	-	Gp-1 +
Ectocarpus fasciculatus	NA	22, Sengco et al	CH92Nie	1992	NA	-	Gp-1 +
Ectocarpus fasciculatus	NA	57, Sengco et al	RUZ7	1970	NA	-	Gp-1 -
Ectocarpus fasciculatus	NA	57, Sengco et al	Ros90-2	1990	NA	-	Gp-1 -
Ectocarpus fasciculatus	NA	68, Sengco et al	HelIII	1960	NA	-	Gp-1 -
Ectocarpus fasciculatus	NA	68, Sengco et al	Hel3-2	1977	NA	-	Gp-1 -
Ectocarpus fasciculatus	NA	68, Sengco et al	Hel31-1	1977	NA	-	Gp-1 -
Ectocarpus siliculosus	NA	69, Sengco et al	Wer	NA	NA	-	Gp-1 +
Ectocarpus siliculosus	NA	57, Sengco et al	Ros93-7-1	1993	NA	-	Gp-1 +
Ectocarpus siliculosus	NA	57, Sengco et al	Ros93-29-3	1993	NA	-	Gp-1 -
Ectocarpus siliculosus	NA	57, Sengco et al	Ros93-32-1	1993	NA	-	Gp-1 +
Ectocarpus siliculosus	NA	73, Sengco et al	CI135-1	1993	NA	-	Gp-1 -
Ectocarpus fasciculatus	NA	73, Sengco et al	CI146-1	1993	NA	-	Gp-1 +
Ectocarpus fasciculatus	NA	4, Sengco et al	Wil1a	1978	NA	-	Gp-1 +
Ectocarpus fasciculatus	NA	81, Sengco et al	SAf10-1	1993	NA	-	Gp-1 -
Ectocarpus fasciculatus	NA	26, Sengco et al	Esta	1988	NA	-	Gp-1 -
Ectocarpus fasciculatus	NA	21, Sengco et al	SC42-3	1991	NA	-	Gp-1 -

Table S2. Summary of phaeoviral infections detected with PCR in kelp sporophytes, kelp gametophytes, and Ectocarpales. See Table S1 for site names key and full sample details. Includes data from this study and previous studies (Mceown et al. 2017; Müller et al. 2000; Sengco et al. 1996).

Host species	Subgroup A	Subgroup B	Subgroup C	Unknown subgroup	Subgroup not tested	Not infected	Total hosts sampled	Total hosts infected	Sites
Ecklonia cava	0	0	0	0	0	2	2	0	96, 98
Ecklonia kurome	0	0	0	0	0	5	5	0	93, 97
Ecklonia maxima	0	0	0	4	0	12	16	4	77, 79, 80, 82-84
Ecklonia radiata	0	0	0	5	0	15	20	5	76, 84-86, 88, 89, 101-103, 105
Ecklonia stolonifera	0	0	0	0	0	1	1	0	99
Ectocarpus crouaniorum	163	52	0	23	0	49	235	186	31-35, 37, 40-43, 47, 50-56, 58, 62
Ectocarpus fasciculatus	49	46	0	28	3	121	219	98	4, 13, 14, 17, 19, 21, 22, 26, 32-34, 36, 38-52, 54-57, 59, 68, 73, 81
Ectocarpus siliculosus	232	107	0	56	42	216	555	339	1-19, 21, 23, 25, 27-33, 36, 40, 43, 44, 46, 47, 50, 52-57, 59-71, 73, 81, 87, 90, 92, 94, 95, 100, 104, 106
Ectocarpus sp.	4	6	0	2	490	84	579	502	24, 34, 36, 39, 40, 46, 53, 74
Kuckuckia sp.	0	0	0	1	0	0	1	0	14
Laminaria digitata	0	0	14	11	0	38	63	25	46, 56
Laminaria hyperborea	0	0	5	0	0	9	14	5	46, 56
Laminaria ochroleuca	0	0	0	0	0	16	16	0	46, 72
Laminaria pallida	0	0	0	0	0	16	16	0	75, 77-80, 82, 83
Lessonia spicata	0	0	0	0	0	5	5	0	20
Macrocystis pyrifera	0	0	1	0	0	4	5	1	20
Saccharina japonica	0	0	0	0	0	5	5	0	91
Saccharina latissima	0	0	7	4	0	28	39	11	46, 56
Undaria pinnatifida	0	0	0	5	0	0	5	5	91

Table S3. GenBank accession numbers of capsid protein sequences from other *Phycodnaviridae* and *Mimiviridae*.

Name	GenBank Accession
Acanthamoeba polyphaga mimivirus	YP_003986929
Acanthamoeba turfacea chlorella virus 1	ABT16414
Acanthamoeba turfacea chlorella virus MN0810	AGE55620
Acanthamoeba turfacea chlorella virus NTS-1	AGE57625
Bathycoccus species virus 1	YP_004061587
Bathycoccus species virus 1	ADQ91330
Cafeteria roenbergensis virus	YP_003969975
Chryochromulina ericina virus 01B	A7U6E7
Ectocarpus fasciculatus 1	HG003334
Ectocarpus siliculosus 1	HG003333
Ectocarpus siliculosus provirus	CBN80416
Ectocarpus siliculosus virus 1	NP_077601
Emiliania huxleyi virus 86	YP_293839.2
Feldmannia irregularis 1	HG003338
Feldmannia irregularis 2	HG003339
Feldmannia irregularis virus 1	AAR26925
Feldmannia simplex 1	HG003340
Feldmannia simplex 2	HG003341
Feldmannia simplex 3	HG003342
Feldmannia simplex 8	HG003337
Feldmannia species virus 158	YP_002154681
Fowlpox virus	AAZ14082
Heterosigma akashiwo virus 01	AOM63514
Megavirus chilensis	YP_004894515
Megavirus courdo11	AFX92519
Micromonas pusilla virus SP1	AET84889
Moumouvirus goulette	AGF85360
Myriotrichia clavaeformis 2	HG003343
Organic lake phycodnavirus 1	ADX05938
Organic lake phycodnavirus 2	ADX06358
Ostreococcus tauri virus 1	YP_003212988
Ostreococcus tauri virus 2	YP_004063587
Ostreococcus tauri virus 5	YP_001648266
Paramecium bursaria Chlorella virus 1	AAA88828
Paramecium bursaria Chlorella virus AR158	ABU44077
Paramecium bursaria Chlorella virus CVK2	BAA35143
Paramecium bursaria Chlorella virus NY2A	ABT14984
Phaeocystis globosa virus	AET73005
Pylaiella littoralis 1	HG003336
Saccharina japonica 1921	JXRI01001921 (Whole genome shotgun, WGS)
Saccharina japonica 145	JXRI01000145 (Whole genome shotgun, WGS)
Saccharina japonica 271	JXRI01000271 (Whole genome shotgun, WGS)
Yellowstone lake phycodnavirus 1	YP_009174754
Yellowstone lake phycodnavirus 2	YP_009174565
Yellowstone lake phycodnavirus 3	YP_009174294

© 2018 by the authors. Licensee MDPI, Basel, Switzerland. This article is an open access article distributed under the terms and conditions of the Creative Commons Attribution (CC BY) license (http://creativecommons.org/licenses/by/4.0/).
